# The PAD-US-AR dataset: Measuring accessible and recreational parks in the contiguous United States

**DOI:** 10.1038/s41597-022-01857-7

**Published:** 2022-12-16

**Authors:** Matthew H. E. M. Browning, Alessandro Rigolon, Scott Ogletree, Ruoyu Wang, Jochem O. Klompmaker, Christopher Bailey, Ryan Gagnon, Peter James

**Affiliations:** 1 Department of Parks, Recreation and Tourism Management, Clemson University, Clemson, South Carolina 29634 USA; 2 NatureQuant LLC, 907 E. 20th Ave., Eugene, OR 97405 USA; 3 Department of City and Metropolitan Planning, The University of Utah, 375 South 1530 East, Salt Lake City, Utah 84112 USA; 4 OPENspace Research Centre, School of Architecture and Landscape Architecture, University of Edinburgh, 74 Lauriston Place, Edinburgh, EH3 9DF UK; 5 Centre for Public Health, Block A, Royal Victoria Hospital, Queen’s University Belfast, Belfast, Northern Ireland United Kingdom; 6 Department of Environmental Health, Harvard T. H. Chan School of Public Health, 655 Huntington Avenue, Boston, Massachusetts 02115 USA; 7 Channing Division of Network Medicine, Department of Medicine, Brigham and Women’s Hospital, 181 Longwood Avenue, Boston, Massachusetts 02115 USA; 8 Department of Population Medicine, Harvard Medical School and Harvard Pilgrim Health Care Institute, 401 Park Drive, Boston, Massachusetts 02215 USA

**Keywords:** Epidemiology, Psychology and behaviour

## Abstract

Most spatial epidemiological studies of nature-health relationships use generalized greenspace measures. For instance, coarse-resolution spatial data containing normalized difference vegetative index (NDVI) values are prominent despite criticisms, such as the inability to restrain exposure estimates to public and private land. Non-threatening natural landscapes can improve health by building capacities for health-promoting behaviors. Recreational and accessible parks may best activate such behaviors. We curated the Parks and Protected Areas Database of the U.S. (PAD-US) to identify parks that are accessible for outdoor recreation. Our title adds “AR” to “PAD-US” where A = Accessible and R = Recreational. We validated the PAD-US-AR by comparisons with greenspace datasets and sociodemographics, which demonstrated its uniqueness from other commonly employed metrics of nature exposure. The PAD-US-AR presents reliable estimates of parks in the contiguous U.S. that are accessible for outdoor recreation. It has strong associations with home prices, shares of female residents, and shares of older residents. This dataset can accompany other nature exposure metrics in environmental epidemiology and allied research fields.

## Abstract (summary)

**Table:** 

Measurement(s)	park
Technology Type(s)	Geographic Information System
Sample Characteristic - Environment	County • Tract
Sample Characteristic - Location	United States

## Background & Summary

Exposure science has historically measured the toxic elements that negatively impact human health^1^. However, nature-rich environments that are perceived as non-threatening can positively influence human health through multiple pathways, including mitigation of harmful exposures (i.e., traffic emissions, heat, and noise), restoring attention and reducing stress, and promoting healthy behaviors (i.e., physical activity, sleep, and social interaction)^2,3^.

Research on the health benefits of nature has grown since the 1990s^4,5^. Hundreds of health outcomes/endpoints have been studied, and at least 40 systematic reviews and meta-analyses have been conducted^6,7^. Collectively, these studies suggest plant-rich environments (“greenspaces”) are associated with lower rates of all-cause and stroke-specific mortality, cardiovascular disease, poor mental health, low birth weight, lower levels of physical activity, and poor sleep quality^6^. Liquid-water environments (“bluespaces”) are associated with lower rates of all-cause mortality, obesity, low levels of physical activity, and poor mental health^8,9^. Finally, solid-water environments (i.e., polar regions) and rock/mineral-dominated landscapes may have emotional and mental benefits and serve as medical treatments for respiratory conditions and allergies, although evidence for these landscapes being therapeutic is minimal^10^.

Despite the growing interest in nature and health, ongoing research would benefit from more sophisticated and precise exposure estimates^11–13^. One simple and imprecise exposure measure of leafy green vegetative cover is the normalized difference vegetation index (NDVI) from moderate resolution (i.e., 30m^2^ or 250m^2^) satellite imagery^2,14,15^. The calculation of NDVI involves determining the ratio between near-infrared and red bands of light^16^. NDVI measures hold some value but are limited in several respects. In defense of NDVI, values have been ground-truthed by environmental psychologists and found to correspond to ratings of “greenness“^17^. Values can also be easily obtained from Google Earth Engine (GEE) at different global spatial and temporal scales. Values are assigned to cells laid out in a grid that overlap land cover types and ownership lines, allowing accurate availability estimates (i.e., magnitude of greenness around the home, work, school, and activity spaces) when available at fine spatial resolutions and coupled with parcel-level ownership data^15,18^. Finally, many vegetation types can activate health-promoting pathways linking nature exposure with health^2^. In critique of NDVI, values cannot indicate the type of, quality of, access to, and experience with vegetation or other forms of nature, such as liquid water, solid water (i.e., ice, snow), or rocks and minerals (i.e., deserts)^2,10,14^. These limitations should not be surprising; after all, the calculation of NDVI is restricted to plants and emerged from agricultural science to estimate crop productivity and expected yield rather than environmental epidemiology^16^. Also limiting NDVI is its inability to identify design characteristics that activate instorative effects of nature-based recreation, such as physical activity along greenways and social interaction at picnic shelters^19,20^. NDVI values are affected by complex interactions between other environmental factors with less relevance to nature exposure, such as season, slope, and precipitation^21,22^ in addition to sensor type and the spatial unit size^23,24^.

Another measure of green vegetation is remotely sensed tree canopy cover. Versions of these data at coarse or moderate resolutions can be easily retrieved (i.e., from the Multi-Resolution Land Characteristics [MRLC] National Land Cover Database [NLCD], see www.mrlc.gov). Higher-resolution data are becoming available from agencies, academic institutions, and commercial providers (i.e., www.earthdefine.com/treemap/, https://insights.sustainability.google/labs/treecanopy) through object-based image analysis and related processes^25–27^. These data can measure this specific type of greenery by classifying vegetation over a certain height (e.g., >2 m) as a tree. Canopy cover is an appropriate nature exposure metric given its opportunities for health promotion through shade, reductions in urban heat island effects, and psychological restoration^28,29^. However, like NDVI, tree canopy cover data do not provide information on public access and recreational opportunities. Such information must be available at high resolution and coupled with parcel-level data or spatial algorithms that differentiate visibility along public rights-of-ways (i.e., sideways in front yards)^30,31^ to identify where trees might be available to the public for recreational opportunities under canopies.

Other advances in the calculation of nature exposure have been made. For instance, machine learning algorithms have been increasingly applied to 360-degree images along streets (e.g., Google Street View [GSV] or Baidu) or photographs looking out windows to calculate the percentage of visible greenery^32–36^. Still, most nature exposure metrics remain limited to greenery or open water cover rather than quantification of recreational facilities (i.e., trails and lightning) that also promote health^37^. The need for alternative datasets remains.

Nationwide data on the location of accessible natural areas managed for outdoor recreation (i.e., parks and protected areas) would be particularly useful. While the composition and facilities in parks vary, many are managed explicitly for the mechanisms explaining the health benefits of nature, including social interaction and physical activity^38,39^^,cf.^^40^. For instance, natural landscapes in rural areas may be used for resource extraction or conservation with few opportunities for recreation^41^. Meanwhile, greenery in urban areas may be intended primarily for ecosystem services such as stormwater runoff, cooling, and noise/air pollution mitigation^42^. Parks across the urban-rural spectrum are important to consider alongside other nature exposure estimates.

Researchers are beginning to use some spatial nationwide datasets for measuring park cover in the U.S. (Table 1). USA Parks was developed by the Environmental Systems Research Institute (Esri) using proprietary data from that company and TomTom^43^. Open Street Map (OSM) includes crowdsourced data tagged by keys (topic/category) and values (features). These can be selected to identify possible public natural areas^44^. The accuracy and consistency of tags vary geographically and are often imprecise, making the identification of public natural areas difficult^45^. ParkServe contains data on local parks in nearly 14,000 cities, towns, and communities in the USA and was curated by the Trust for Public Land (TPL)^46^. Finally, the Parks and Protected Areas Database United States (PAD-US) is an initiative of the U.S. Geological Survey (USGS) with federal, state, and local partners^47^. It hopes to inventory all protected areas, including public lands, and voluntarily provide private protected areas.

**Table 1:** Description of park cover datasets for the contiguous U.S.

Name	Developers	Updated	Description	Source	License
USA Parks^43^	Environmental Systems Research Institute (Esri)	09–2021	“National and State parks and forests, along with County, Regional and Local parks within the United States… provides thousands of named parks and forests at many levels.”	Esri, TomTom	Esri Master License Agreement
OSM^44^	Open Street Map (OSM) Foundation	(continuous)	Park data are available by selecting relevant tags, consisting of a key and value separated by a colon. The key is a topic, category, or type of feature (i.e., areas used for leisure). The value provides detail for the key-specified feature (i.e., park vs. playground, both of which are used for leisure). Tags used in past research on park cover and greenspace measures vary but can include leisure = park, leisure = garden, landuse = grass^45^,^83^; playground and protected_area^84^; dog park and flower bed^85^; and allotment, cemetery, farmland/farmyard, forest/wood, greenfield, greenhouse, meadow, nature reserve, orchard, plant nursery, scrub, village green, and wetland^86^. Golf courses have been excluded from some greenspace analyses^68^.	Crowdsourced	Open Database License
ParkServe^46^	Trust for Public Land (TPL)	05–2022	“a comprehensive database of local parks in nearly 14,000 cities, towns, and communities… attempted to contact each city, town, and community with a request for their parks data. If no GIS data was provided, [TPL] created GIS data for the place based on available resources, such as park information from municipal websites, GIS data available from counties and states, and satellite imagery.”	Municipal, county, and state GIS datasets; Satellite imagery	Copyright held by the TPL; Data available for personal, non-commercial use
PAD-US V2.1^47^	United States Geological Survey (USGS)	09–2020	“Nation’s inventory of protected areas, including public land and voluntarily provided private protected areas… an ongoing project with several published versions of a spatial database including areas dedicated to the preservation of biological diversity, and other natural (including extraction), recreational, or cultural uses, managed for these purposes through legal or other effective means… its scope expanded in recent years to include all public and nonprofit lands and waters… strives to be a complete inventory of public land and other protected areas, compiling ‘best available’ data provided by managing agencies and organizations.”	Federal, state, and local agencies; National Conservation Easement Database; ParkServe	Public domain
PAD-US-AR V1^48^	The Authors and USGS Data	12–2020	A curated version of the PAD-US that identifies parks intended for recreation and accessible to the general public.	PAD-US V2.1	Creative Commons Attribution 4.0 International

Notes: Descriptions were retrieved on June 6, 2022.

These currently available park datasets are limited in identifying where accessible and recreational parks exist. Most lack metadata on whether each land parcel is open to the public. OSM provides some data on public access but without clear assignments. For example, our retrieval of polygons with the “leisure:park” tag returned 17 types of access from “community” and “discouraged” to “permissive,” “yes,” “restricted,” and “unknown.” Further, OSM data are crowdsourced and not validated by the agencies who manage these spaces. ParkServe also has public access metadata, but its coverage is focused on municipalities. Park cover in rural areas where many important recreational parks (i.e., National Parks) are located is limited in ParkServe.

In response to the value of park data and limitations with extant datasets, we present a new exposure indicator – the dataset for accessible and recreational parks in the contiguous United States (PAD-US-AR). We validate this dataset by comparing it to its source (the original PAD-US V2.1), other nature exposure metrics, including NDVI, tree canopy cover, alternative park datasets, and sociodemographic characteristics in counties and states.

## Methods

We curated the  PAD-US-AR^48^ dataset from the USGS Protected Areas Database of the U.S. V2.1 (PAD-US V2.1)^47^. The PAD-US is published by U.S. Geological Service in collaboration with Boise State University and through coordination with Federal, State, and non-governmental organizations that provide and verify the data. Its original release was in April 2009. Updates were made in 2010, 2011, 2012, 2016, 2018, 2020, and July 2022. Data on the completeness of the V2.0 dataset, which occurred before the V2.1 dataset used here, are available at https://www.protectedlands.net/frequently-asked-questions-about-pad-us/. In brief, 14 states had over 95% coverage of parks and protected areas, 26 states had 80–95% coverage, and the remaining 8 states in the contiguous U.S. had <80% coverage. Updated coverage statistics for V2.1 are currently unavailable.

The PAD-US is a regularly updated geographic information system (GIS) spatial dataset that compiles the best available data provided by U.S.-based land management agencies and organizations. It strives to be a complete inventory of public land and other protected areas in the U.S. Included areas are those preserved for biological diversity and other natural, recreation, historical, or cultural uses and managed for these purposes through legal or other effective means^47^. Some areas consist of small land parcels with building footprints that occupy most of the area. These are not readily identified with the PAD-US V2.1 metadata. The location designation field (Loc_Ds) offers some clues with values such as “cultural arts center” and “National Register of Historic Places.” The number of unique values (N = 1,675) in the designation, easement, and fee areas of the PAD-US V2.1 limits precise identifications and removal of such areas.

The PAD-US V2.1 release became available in September 2020 and included notable updates from previous versions. These included integration of the TPL ParkServe dataset, Census American Indian/Alaskan Native Areas, Ducks Unlimited protected areas, and federal land ownership updates, among others. The PAD-US V3.0 was released in early July 2022 and contained minor updates that we expected to influence the curation process of the PAD-US-AR very little. For a complete description of version updates, see https://www.usgs.gov/programs/gap-analysis-project/pad-us-data-history.

The PAD-US has been used for conservation mapping^49–55^ and noise research^56,57^. These studies have identified that Western U.S. National Monuments provided jobs and economic growth after establishment^52^, counties with greater coverage of protected areas with strict conservation status (i.e., Wilderness Areas and National Parks) are associated with higher average noise levels^56^, and anthropogenic noise is common in many U.S. parks and protected areas^57^. We are also aware of a few nature-health studies that have utilized the complete PAD-US dataset^58,59^. In studies by Tsai and colleagues, the authors identified park locations and ground-truthed results with Google Maps and county/municipal data to identify park entrances. The PAD-US was used to calculate descriptive sample characteristics or covariates in models with other measures of nature exposure (i.e., tree cover and greenery), so associations between health and the PAD-US were not reported.

The opportunities and lack of precedent for curations of the PAD-US prompted us to define which types of parks and protected areas in the dataset were both accessible and recreation-oriented. Based on discussions among three authors (M.B., A.R., S.O.) and three outdoor recreation specialists in the western United States, we reached a consensus on including the following categories:

1. Parks open for public access or restricted access (i.e., seasonally open, fees required, or permits required), including but not limited to lands managed by the National Park Service, U.S. Forest Service, Bureau of Land Management, U.S. Fish & Wildlife, Army Corps of Engineers, State Parks, State Departments of Conservation, State Departments of Natural Resources, State Departments of Land, State Fish and Wildlife Departments, State Forest Service, State Park and Recreation Departments, Tennessee Valley Authority, and city and county park and recreation departments.
2. Publicly accessible conservation easements.

We excluded the following designations (see the paragraphs below for rationales):

1. Department of Energy, Department of Defense, and Bureau of Reclamation lands
2. Marine areas managed as Marine Protected Areas by the National Oceanic and Atmospheric Administration, or Bureau of Ocean Energy Management, among others
3. Proclamation areas, which are boundaries of national lands used for administrative purposes that overlap with large areas of public lands that are not all available to the public
4. Fish hatcheries and other lands used for water rights with regulated hunting
5. National Park easements (i.e., lands paralleling but not including the Appalachian Trail and not used by the public)
6. Joint management areas (i.e., university research stations)
7. Non-governmental organization lands (aside from conservation easements)
8. State trust/land survey lands
9. American Indian Lands
10. Other areas with unknown access or closed public access (i.e., limited to coordinated programs and research)

**Fig. 1:**
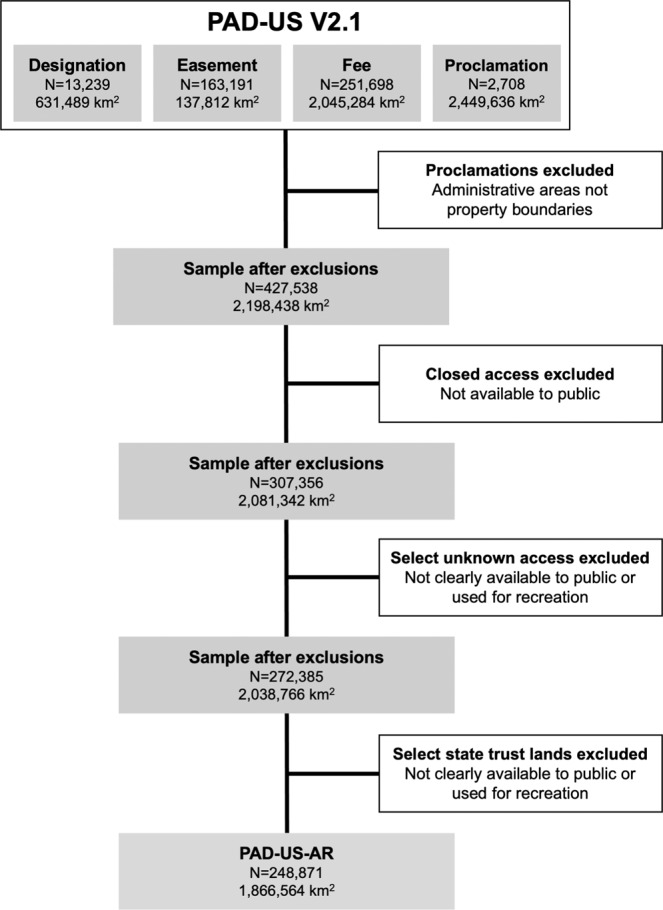
Data curation of the PAD-US-AR from the PAD-US V2.1.

Restricting the PAD-US to these categories was a sequential process starting with the four terrestrial PAD-US domains (Fig. 1). These domains included designations (policy-designated areas such as National Parks and State Parks), easements (conservation and open space easements provided by the National Conservation Easement Database^60^), fee lands (open space owned by Federal, State, or local agencies, nonprofits, or private individuals), and proclamations (boundaries of administrative areas). For further information on these domains, see http://www.protectedlands.net/pad-us-technical-how-tos/.

Our first step was to exclude all proclamation lands in the PAD-US. These administrative boundaries are not ownership lines but are instead the outer boundaries of areas used by land managers for planning regardless of internal ownership. They could but will not necessarily be publicly managed in the future. Some commercial mapping providers (i.e., Google Maps, Esri USA Parks) incorrectly use these boundaries to show protected areas and, in doing so, often show large areas of private lands as part of public lands.

Next, we excluded lands described as closed to public access in the PAD-US. Alternative classifications include open to public access, restricted, which denotes a permit may be needed, or unknown. We temporarily retained unknown access areas for further consideration since large areas of the intermountain west are designated as such. For example, the Great Salt Lake, UT, is the state’s largest water body and a recreation destination for boating, swimming, and sunbathing.

The subsequent step was refining lands labeled as unknown access in the PAD-US. Decisions were made based on the assigned land manager. City lands (Code = CITY) were included since many greenways were under this classification. County lands (CNTY), which described nearly 250 polygons run by the City of New York for parks and recreation in the city and upstate, were included. Similarly, regional agency land (REG) covered over 400 polygons concentrated in Chicago and Los Angeles suburbs used for parks and recreation; these lands were retained. State Department of Conservation (SDC) and State Department of Natural Resource (SDNR) lands were also retained. These included over 5,000 polygons across the country, including the Great Swamp Management Area, RI, an important area for birding and open to the public, and the Great Salt Lake. State Department of Land (SDOL) areas were retained, as they included approximately 30 polygons used by the public for hiking in Northwestern states. State Fish and Wildlife (SFW) lands included urban areas with trails along waterways and were retained. State Parks and Recreation (SPR) lands were retained and covered public recreational areas in Maine. Tennessee Valley Authority (TVA) and Army Corps of Engineers (USACE) areas covered large reservoirs with important water-based recreation resources and were retained. The presence of such waterbodies, which provide public recreation to millions of visitors annually^61^, required us to retain the entire census geographies despite evidence that removing areas covered by water can lead to more precise and realistic sociodemographic analyses^62^. Last, U.S. Forest Service (USFS) lands were retained as they included several recreational areas in Virginia.

All other areas with unknown public access in the PAD-US were deemed not accessible to the public or used for public recreation and therefore excluded. This conservative approach reduced the chances of misclassifying large tracts of land that were likely inaccessible. For example, Department of Defense (DOD) lands included ammunition plants, Department of Energy (DOE) lands included nuclear test sites, and National Oceanic and Atmospheric Administration (NOAA) lands included estuarine research reserves. Non-governmental organization (NGO) lands included nearly 17,500 polygons in the Rocky Mountains but covered too many conservation types to determine whether these were open to the public. American Indian Lands (TRIB) were on reservations and could not be assumed to be accessible and used by the public.

The final step in curating the PAD-US-AR dataset was determining how to approach the polygons in the Western and Midwestern states that were left over from the Public Land Survey System (designation = SRMA). Most of these lands follow a grid pattern and are not used for outdoor recreation. However, some state trust lands include important parks, such as DuPont State Forest, NC, a popular destination for mountain biking, hiking, swimming, and visiting waterfalls. Three of the authors conducted online searches of the uses of these lands using online resources (i.e., State Department of Natural Resource portals) for each state and selected which to include or exclude. The corresponding author also discussed these decisions with three outdoor recreation professionals living in the western U.S. Based on this examination, we removed state trust lands from Arizona, Colorado, Idaho, Louisiana, Mississippi, Montana, Nevada, New Mexico, North Dakota, Oklahoma, Oregon, South Dakota, Texas, Utah, Washington, and Wyoming.

To obtain census tract and county exposure estimates, we calculated the percentage of the PAD-US-AR covering each geographic unit. Tract-level estimates included a 0.5-mile buffer around each tract to acknowledge the opportunities for park access for residents living near the tract boundaries. Similar thresholds have been used in past research^63–65^ and are recommended as U.S. park access standards by several nonprofits (e.g., Trust for Public Land, www.10minutewalk.org). This threshold is primarily used in urban areas and may be most relevant to those areas where most people live and where tract sizes are smaller.

No buffering was conducted around counties. Counties are >300% larger than tracts, on average. In our dataset, the median county area was 1,614 km^2^, while the median tract area was 5 km^2^. Counties are also jurisdictions of local governments, whereas tracts do not represent any administrative boundaries. For these reasons, we avoided buffering counties, which often have parks and recreation departments managing parks within their borders.

## Data Records

PAD-US-AR^48^ data are released under the Creative Commons Attribution 4.0 International (CC BY 4.0) license and publicly available on an Open Science Framework (OSF) repository (10.17605/OSF.IO/PWDSG). Several files are available:

1. Geopackage and shapefile of the PAD-US-AR^48^ in a standard format (separate polygons for different parks) and dissolved format (a single polygonal layer)
2. Spreadsheets of PAD-US-AR^48^ cover in 2019 U.S. counties
3. Spreadsheets of PAD-US-AR^48^ cover in 2019 U.S. ZIP code tabulation areas
4. Spreadsheets of PAD-US-AR^48^ cover in 2019 U.S. tracts with 0.5-mile buffers around each tract

Geopackage and shapefiles include vector polygons with the original metadata from the PAD-US V2.1. For a complete listing of variables, please visit https://www.usgs.gov/programs/gap-analysis-project/pad-us-data-manual. In brief, the data include the name of the parcel; feature class (in the PAD-US-AR, the options are designation, easement, or fee); type and name of management agency (i.e., federal, state, American Indian Lands, or local government); designation (i.e., conversation easement vs. National Park); conservation protection level as designated by the International Union for the Conservation of Nature (IUCN); state name; and geographic size.

Spreadsheets include geographic identifiers (i.e., FIPS codes or GEOID) and percent park cover. These are provided as Microsoft Excel files (.xlsx) and text files (.txt) to maintain leading zeros in the geographic identifiers. Park cover ranges from 0 (no parks) to 100 (complete park cover). Tract estimates are provided for park cover within the boundaries of each tract and the 0.5-mile buffered tract boundaries.

## Technical Validation

The PAD-US-AR^48^ dataset presents park cover from nearly 250,000 spatial units and 1,900,000 km^2^ in area across the contiguous U.S (Table 2). Histograms of the data within counties and tracts and by census region are presented in Figure S2. Distributions were right skewed in all regions except Northeastern and Western counties. Northeastern counties showed a flat distribution until approximately 20% cover. Higher levels of cover were present in few counties. Western counties showed a roughly flat distribution until around 80% cover, after which the number of counties with higher cover levels was small.

**Table 2:** Number of units and cover of datasets for park cover in the contiguous U.S.

Name	Nationwide	Northeast	Midwest	South	West
USA Parks	61,030 (1,409,517 km^2^)	9,724 (52,082 km^2^)	17,075 (110,524 km^2^)	16,657 (172,960 km^2^)	17,665 (700,588 km^2^)
ParkServe	135,179 (574,333 km^2^)	29,238 (18,672 km^2^)	35,251 (52,267 km^2^)	37,647 (48,153 km^2^)	33,008 (453,441 km^2^)
OSM leisure tags	309,160 (764,141 km^2^)	64,442 (51,179 km^2^)	90,833 (77,872 km^2^)	72,046 (95,613 km^2^)	80,113 (522,610 km^2^)
OSM boundary tags	51,966 (1,195,726 km^2^)	17,174 (69,626 km^2^)	8,257 (80,742 km^2^)	11,670 (138,321 km^2^)	13,760 (890,270 km^2^)
PAD-US V2.1	430,836 (3,619,326 km^2^)	110,327 (117,498 km^2^)	118,496 (817,404 km^2^)	90,815 (438,958 km^2^)	110,650 (2,094,608 km^2^)
PAD-US-AR^48^	248,871 (1,866,564 km^2^)	63,530 (68,037 km^2^)	69,493 (161,952 km^2^)	54,456 (172,563 km^2^)	61,693 (1,464,012 km^2^)

Notes: Areal statistics were calculated by dissolving all polygons and determining cover rather than meta-data provided in the original data. Sum of the areas within regions may not total the nationwide statistics in polygons extending beyond the terrestrial scope of the U.S. and polygons overlapping regions. OSM tags included dog_park, garden, nature_reserve, and park for the leisure key and national_park and protected_area for the boundary key based on past research utilizing OSM for park cover^45,68,83–86^. PAD-US-AR values differ from Fig. 1 because those values were intended to show the number of units/aerial cover lost at each curation stage. In contrast, these values were designed to compare park datasets and report results after dissolving park polygons.

**Fig. 2:**
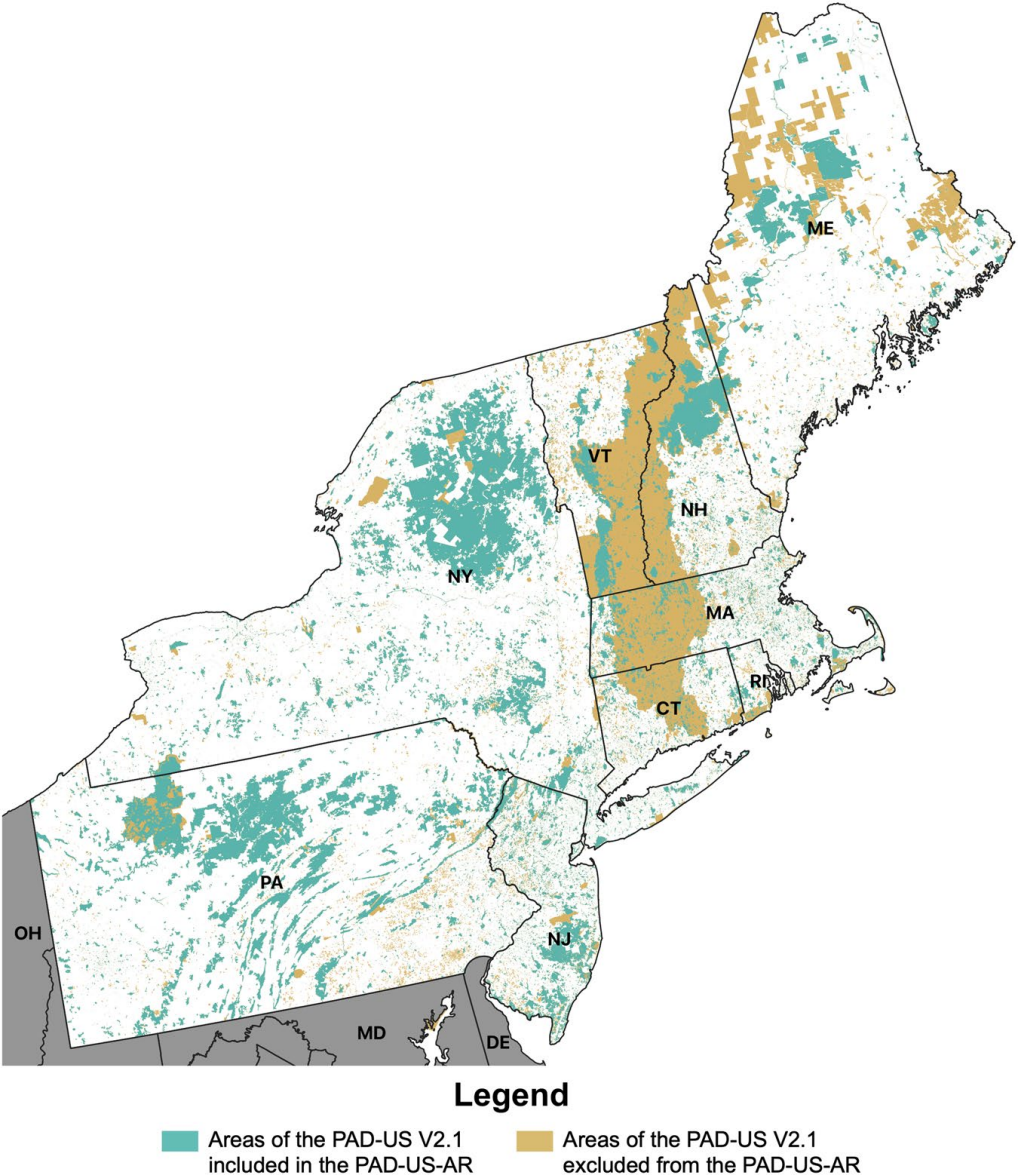
PAD-US-AR park cover compared with its source dataset (PAD-US V2.1) in Northeastern states (57.9% of the total park cover was retained).

**Fig. 3:**
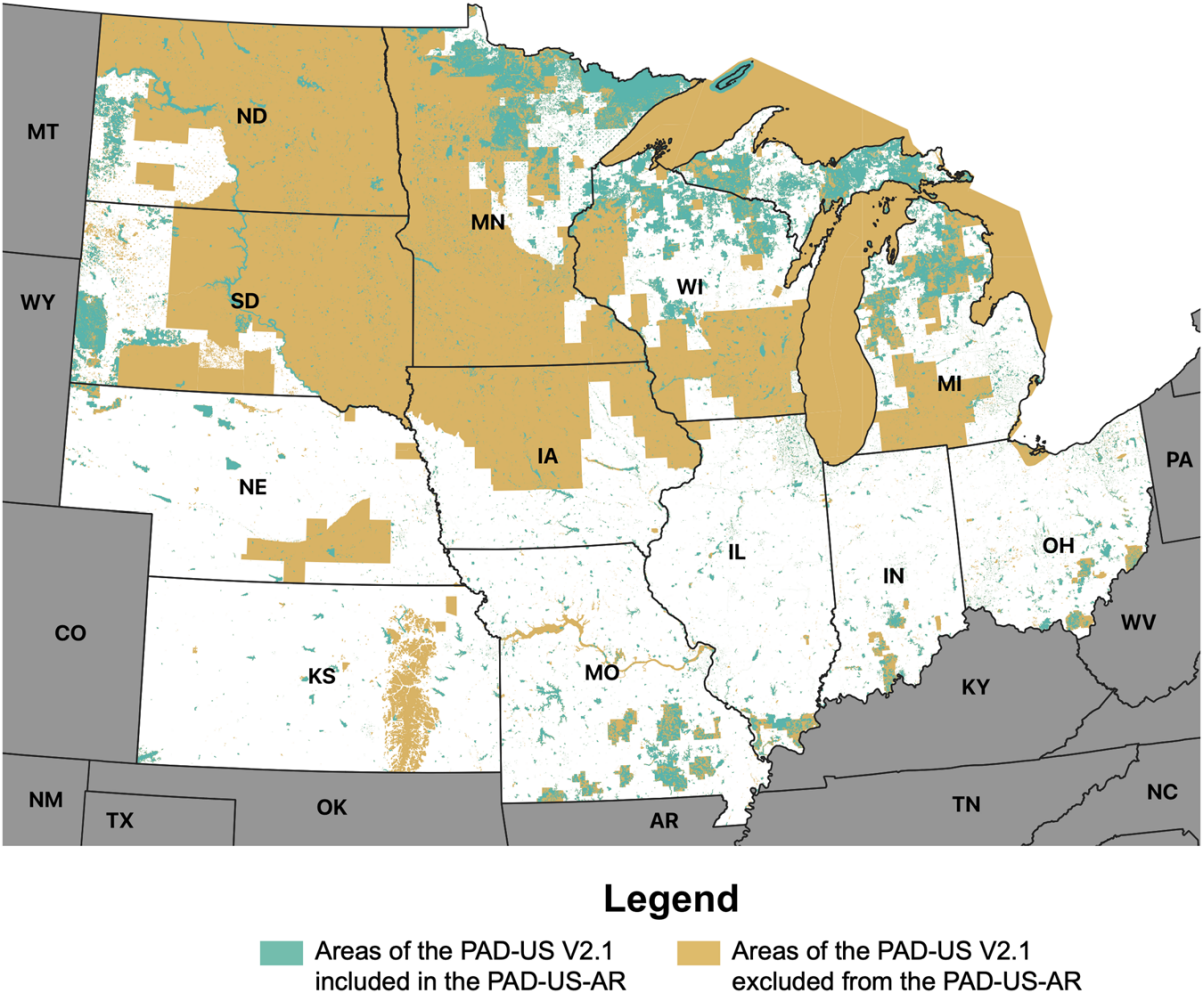
PAD-US-AR park cover compared with its source dataset (PAD-US V2.1) in Midwestern states (19.8% of the total park cover was retained).

**Fig. 4:**
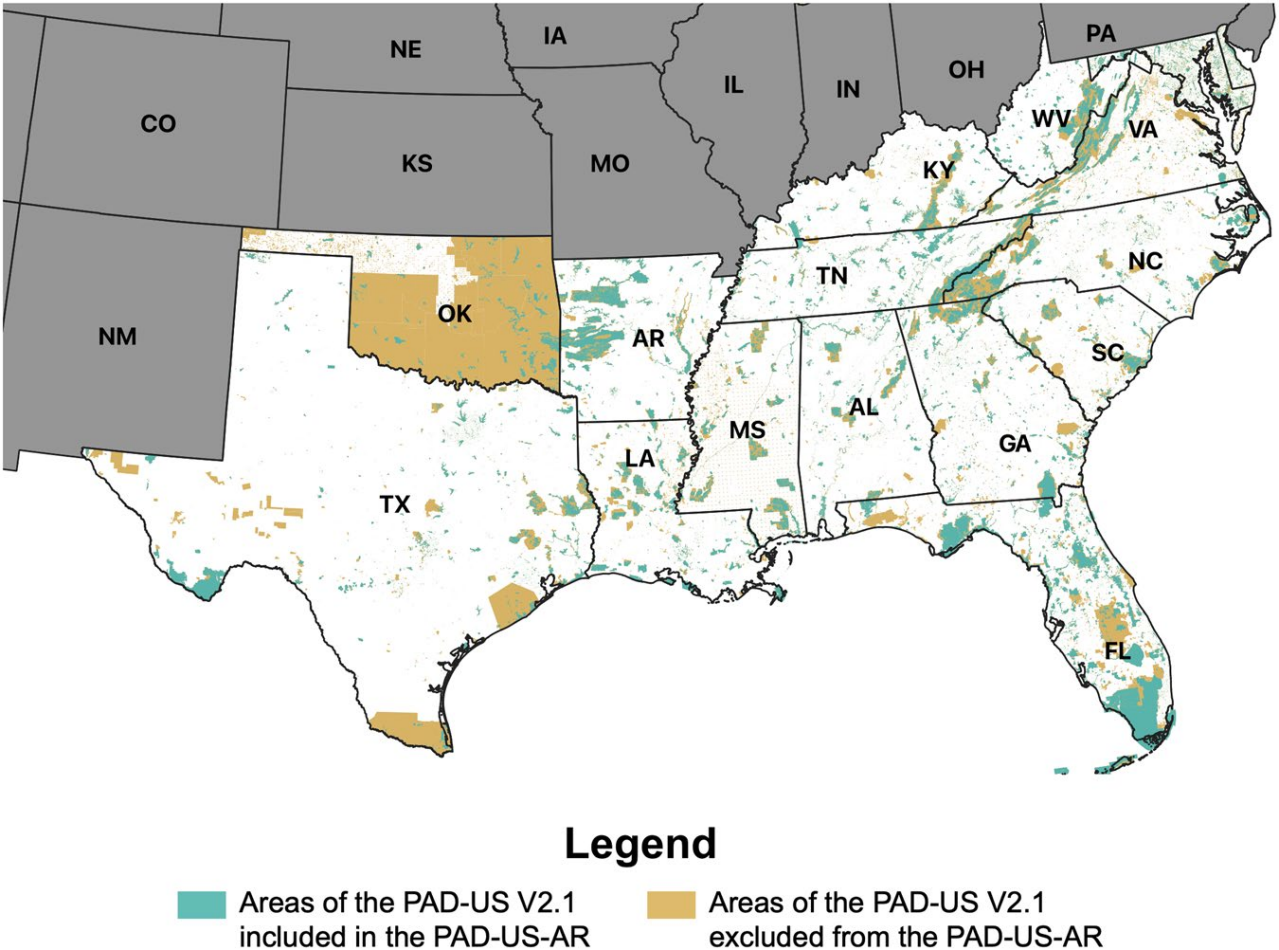
PAD-US-AR park cover compared with its source dataset (PAD-US V2.1) in Southern states (39.3% of the total park cover was retained).

**Fig. 5:**
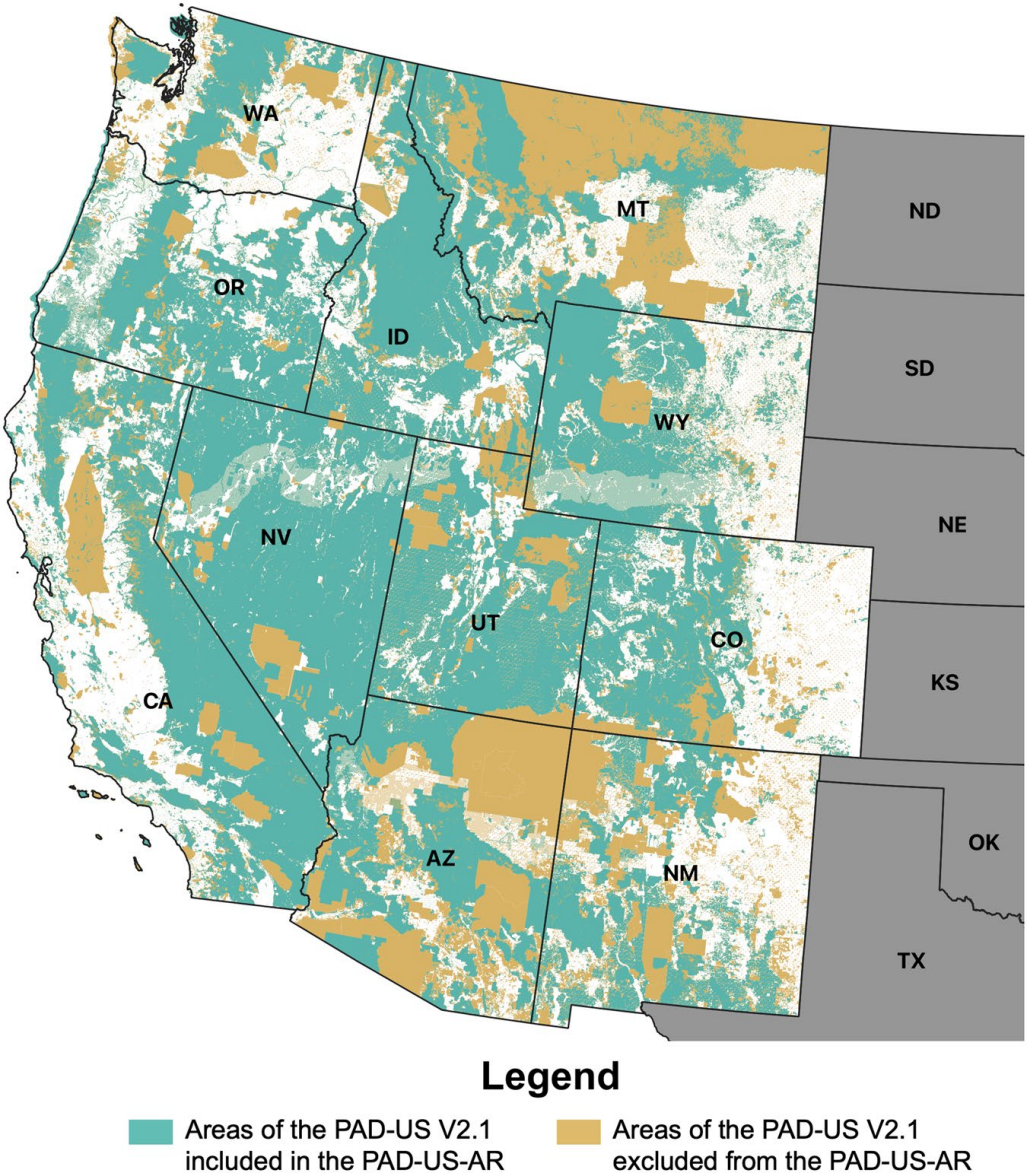
PAD-US-AR park cover compared with its source dataset (PAD-US V2.1) in Western states (69.9% of the total park cover was retained).

Comparisons with the source dataset (PAD-US V2.1) are available for each census region in Figs. 2–5. Large areas of Maine, southeast Pennsylvania, central/western Massachusetts, and northern New Hampshire were excluded from the PAD-US-AR because they were private conservation easements, watersheds with closed access as listed in the PAD-US V2.1, or otherwise unknown public access. Swaths of the Dakotas were removed as conservation easements used for wildlife management with uncertain public access. Lands in Oklahoma arranged on a gridwork were removed as state school lands typically leased out for agriculture and mineral resource purposes. A gridwork of land parcels in Montana, Wyoming, Colorado, Arizona, and New Mexico was removed as state trust lands managed for timber, surface, and mineral resource extraction. Similarly, larger parcels of state trust lands in Western Texas were excluded. Other large parcels of lands excluded were over 560,000 acres in central Idaho, 860,00 acres in southern Nevada, and nearly 200,000 acres in southern South Carolina managed by the Department of Energy; approximately 550,000 acres at Vermejo Park Ranch managed by Ted Turner Reserves, Inc., and 133,000 areas of the Stronghold District of Badlands National Park in western South Dakota owned by the Oglala Sioux Tribe under agreement by the National Park Service.

Next, we compare the PAD-US-AR dataset with other park datasets, nature exposure metrics, and sociodemographic characteristics. The value of comparing the PAD-US-AR with other park datasets is to determine whether the PAD-US-AR differs from already available datasets. Park dataset comparisons were made by tallying the number of geographic polygon units and calculating the total cover after dissolving all polygon units (to account for some polygons overlapping each other) in census regions.

The value of comparing the PAD-US-AR to nature exposure metrics is to determine whether park cover differs from other standard exposure estimates. We employed two measures of NDVI (annual averages and summertime highs) and tree canopy cover, which were derived from raster images and averaged across geographic units (tracts or counties). NDVI values were retrieved and processed in Google Earth Engine (GEE) using cumulative annuals or summertime highs (June-August) from 250 × 250 m 16-day MODIS images averaged over five years (2015–2020) after extracting cloud cover and water pixels. Tree canopy data were retrieved from the 2019 National Land Cover Database (NLCD) release^66^, which provides cover estimates ranging from 0 to 100% for each 30 × 30 m pixel in 2016. This release was the most recent available during data retrieval (September 2022). To identify whether the PAD-US-AR was unique from these other estimates of nature exposure, we examined bivariate correlations between each metric and the PAD-US-AR.

Last, we examined sociodemographic correlates of park cover measured through the PAD-US-AR to inform what confounding factors should be considered when modeling associations between park cover and human health. Sociodemographic characteristics were retrieved from 2015–2019 American Community Survey (ACS) estimates from the U.S. Census at the county and tract level^67^. We selected 14 variables (Table S2) based on existing literature examining correlates of greenspace, especially in studies focusing on socioeconomic and racial disparities in access to these spaces^68–72^. Attempts at incorporating median household income alongside other measures resulted in multicollinearity, so this variable was excluded from the primary analyses but considered in a sensitivity analysis. We examined the results of generalized linear mixed models (GLMMs) with gamma distributions and U.S. states as random effects to account for the non-normal distribution of the outcome variable and the hierarchical nature of the data (counties and tracts within states). Models were run with complete data for 100% of counties (N = 3,108) and 97.3% of tracts (N = 70,580) in the contiguous U.S. circa 2019.

Stratified analyses using more urbanized counties (≥50 people/km^2^) and tracts (≥1,000 people/km^2^) were conducted to compare results with past research and inform future scholarship with the PAD-US-AR. There is no consensus on differentiating more vs. less urban areas in nature-health research^19^. Between 1,000 and 1,999 people/km^2^ is a common cut point^19^. We attempted to apply that cut point to both units of analysis (tracts and counties), which split the number of tracts roughly in half (n = 32,929 as more urban). In contrast, this cut point resulted in too few counties to conduct sufficiently powered analyses (N = 45 as more urban). We attempted the 300 people/km^2^ cut point recommended by the European Union^73^ and used in a recent U.S. study on the association between park cover, park use, and mental health^74^. This continued to produce small sample sizes: N = 43 for the Northeast, 30 for the Midwest, 93 for the South, and 16 for the West. A cut point of 50 people/km^2^ produced reasonable sample sizes for most regions (N = 121 for the Northeast, 178 for the Midwest, 386 for the South, and 58 for the West). Applying this 50 people/km^2^ cut point to counties also produced maps that approximated the location of the Census classification of urbanized areas (https://www.census.gov/programs-surveys/geography/guidance/geo-areas/urban-rural.html; Figure S2). This urbanized area classification scheme has been used to create other datasets on environmental exposure estimates, such as urban heat island vulnerability^75^. GLMMs were used in these stratified analyses except in the Midwest, where standard linear regression models were run to avoid singularity resulting from few urban counties per state in the random effect term.

### Comparison of the PAD-US-AR percent park cover dataset to other park datasets

Descriptive statistics for each park dataset are provided in Table 2, and maps of park cover are provided in Figure S3. The PAD-US-AR^48^ covers 51.6% of the acreage in the PAD-US V2.1 dataset. The PAD-US-AR acreage is larger than the acreage of USA Parks and ParkServe but smaller than the OSM datasets when leisure and boundary tags are combined. Bureau of Land Management (BLM) lands are mainly absent from the USA Parks and ParkServe datasets but are partially included in the OSM datasets and prominent in the PAD-US-AR. This is particularly noticeable in Nevada, western Utah, and Wyoming. These areas include such popular recreation attractions as the Grand Staircase-Escalante National Monument, UT, and the Grand Canyon Parashant National Monument, AZ. These collectively encompass nearly 3,000,000 acres (around twice the size of Delaware), attract more than 150,000 visitors annually for hiking, backpacking, and camping, and have received thousands of 5-star reviews on Google Maps. This high number of reviews shows their popularity and visibility in the public sphere. Other notable areas include off-highway vehicle (OHV) trails, such as the Little Sahara OHV Area, UT, which offers driving/riding on a 700-foot drivable sand dune, 30,000 annual visitors, four campgrounds, and approximately 62,000 acres. Most popular mountain biking and OHV riding trails around Moab, UT (except for the Slick Rock Trail System) are also BLM lands excluded or with limited coverage in datasets other than the PAD-US and PAD-US-AR. These results demonstrate that the PAD-US-AR presents a selected sample of the PAD-US dataset with differing coverage from pre-existing park cover datasets.

### Comparison of the PAD-US-AR to other nature exposure measures

Descriptive statistics for park cover and  other nature exposure metrics are presented in Table 3. Maps of each metric are provided in Figure S4. Distributions of nature exposure metrics are available in Figures S5-S7.

**Table 3:** Descriptive statistics for the PAD-US-AR and other nature exposure metrics.

	Counties				Tracts			
	*N*	*Med*	*IQR*	*Range*	*N*	*Med*	*IQR*	*Range*
	**Nationwide**							
Public park cover	3108	0.04	0.12	0.97 (0–0.97)	70580	0.03	0.07	1 (0–1)
Tree canopy cover	3108	0.27	0.47	0.87 (0–0.87)	70580	0.13	0.30	0.93 (0–0.93)
NDVI annual average	3108	0.53	0.22	0.66 (0.12–0.78)	70527	0.46	0.21	0.82 (0.02–0.83)
NDVI summertime max	3108	0.82	0.19	0.79 (0.13–0.93)	70446	0.65	0.31	1.04 (−0.11–0.93)
	**Northeast**							
Public park cover	217	0.11	0.12	0.79 (0.01–0.79)	13022	0.05	0.08	0.88 (0–0.88)
Tree canopy cover	217	0.53	0.24	0.75 (0.03–0.77)	13022	0.24	0.37	0.83 (0–0.83)
NDVI annual average	217	0.58	0.05	0.43 (0.22–0.65)	12970	0.49	0.23	0.69 (0.02–0.71)
NDVI summertime max	217	0.86	0.07	0.57 (0.35–0.93)	12893	0.71	0.29	1.04 (−0.11–0.93)
	**Midwest**							
Public park cover	1055	0.02	0.05	0.7 (0–0.7)	16762	0.04	0.07	0.88 (0–0.88)
Tree canopy cover	1055	0.08	0.21	0.75 (0–0.75)	16762	0.10	0.16	0.78 (0–0.78)
NDVI annual average	1055	0.45	0.11	0.41 (0.25–0.66)	16762	0.44	0.11	0.65 (0.02–0.67)
NDVI summertime max	1055	0.84	0.10	0.72 (0.2–0.91)	16762	0.71	0.21	0.88 (0.04–0.92)
	**South**							
Public park cover	1422	0.03	0.09	0.7 (0–0.7)	25601	0.02	0.06	0.91 (0–0.91)
Tree canopy cover	1422	0.47	0.37	0.87 (0–0.87)	25601	0.26	0.36	0.93 (0–0.93)
NDVI annual average	1422	0.63	0.11	0.57 (0.17–0.74)	25600	0.55	0.17	0.69 (0.08–0.77)
NDVI summertime max	1422	0.83	0.11	0.7 (0.21–0.91)	25596	0.70	0.24	0.84 (0.09–0.93)
	**West**							
Public park cover	414	0.40	0.47	0.97 (0–0.97)	15195	0.04	0.10	1 (0–1)
Tree canopy cover	414	0.09	0.21	0.66 (0–0.66)	15195	0.02	0.06	0.74 (0–0.74)
NDVI annual average	414	0.31	0.15	0.66 (0.12–0.78)	15195	0.31	0.17	0.78 (0.05–0.83)
NDVI summertime max	414	0.46	0.24	0.74 (0.13–0.88)	15195	0.36	0.22	0.85 (0.05–0.91)

Note: Med. = median, IQR = interquartile range; differences in tract-level sample sizes across exposure metrics resulted from Google Earth Engine not providing complete data from MODIS imagery across study years and regions.

**Fig. 6:**
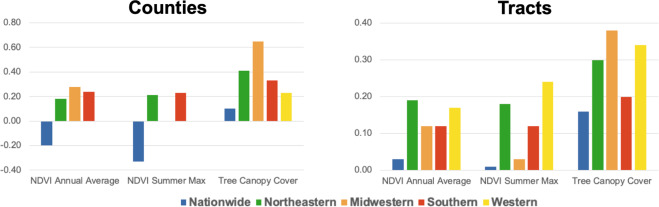
Correlations between park cover and nature exposure metrics across the contiguous U.S. within counties and tracts. Notes: Pearson correlation coefficients.

Associations between the PAD-US-AR^48^ and NDVI varied across geographies and seasons (Fig. 6). Park cover was negatively associated with NDVI at the county level. Pearson correlation coefficients (r[95% confidence interval]) were as follows: r_annual_ = −0.21[−0.24, −0.17]; r_summer_ = −0.33[−0.36, −0.30]. Park cover was not correlated with NDVI at the tract level (r_annual_ = 0.03[0.02, 0.04]; r_summer_ = 0.01[0.00, 0.02]). Associations between the PAD-US-AR and NDVI within census regions were consistently positive, except in Western counties (r_annual_ = −0.12[−0.21, −0.02]; r_summer_ = −0.03 [−0.12, 0.07]) or with NDVI summertime maximums in Midwestern counties (r = −0.02[−0.08, 0.04]). Such results are likely due to climatic and land use differences, such as arid climates in the West and high concentrations of agricultural land that only produces chlorophyll in the summer in the Midwest. Meanwhile, associations between park cover and NDVI annual averages in Midwestern counties were the strongest observed among any pairing (r = 0.28[0.22, 0.33]). This may be explained by parkland in the upper Midwest having higher concentrations of vegetation that produce chlorophyll year-round (i.e., evergreen trees, herbaceous wetland cover) than in the South and fewer urban parks with less greenery than in the Northeast. Associations at the tract level ranged from 0.03[0.02, 0.05] for NDVI summertime maximums in Midwestern tracts, where agricultural lands may only be green in the summer, to 0.23[0.22, 0.25] for NDVI summertime maximums in Western tracts.

Park cover was positively associated with tree canopy cover in every pairing. The strongest correlations were among Midwestern counties (r = 0.65[0.61, 0.68]), and the weakest correlations  were in nationwide county-level models (r = 0.10[0.07, 0.14]). The consistent correlation between canopy cover and parks may be explained by people’s innate preference for open-growth trees with large amounts of canopy cover^29,76–78^ and historical guidelines to retain such trees in park design^79^.

These findings demonstrate that the PAD-US-AR^48^ presents a unique exposure estimate from metrics of nature exposure. Plant-rich landscapes, or “greenspaces,” do not capture all aspects of open recreational spaces and nature-rich landscapes^10^. Correlations between nature exposure metrics vary in size and direction based on the unit of analysis (counties vs. tracts) and geography (regions of the country and nationwide analyses).

### Comparison of the PAD-US-AR to sociodemographic characteristics

**Fig. 7:**
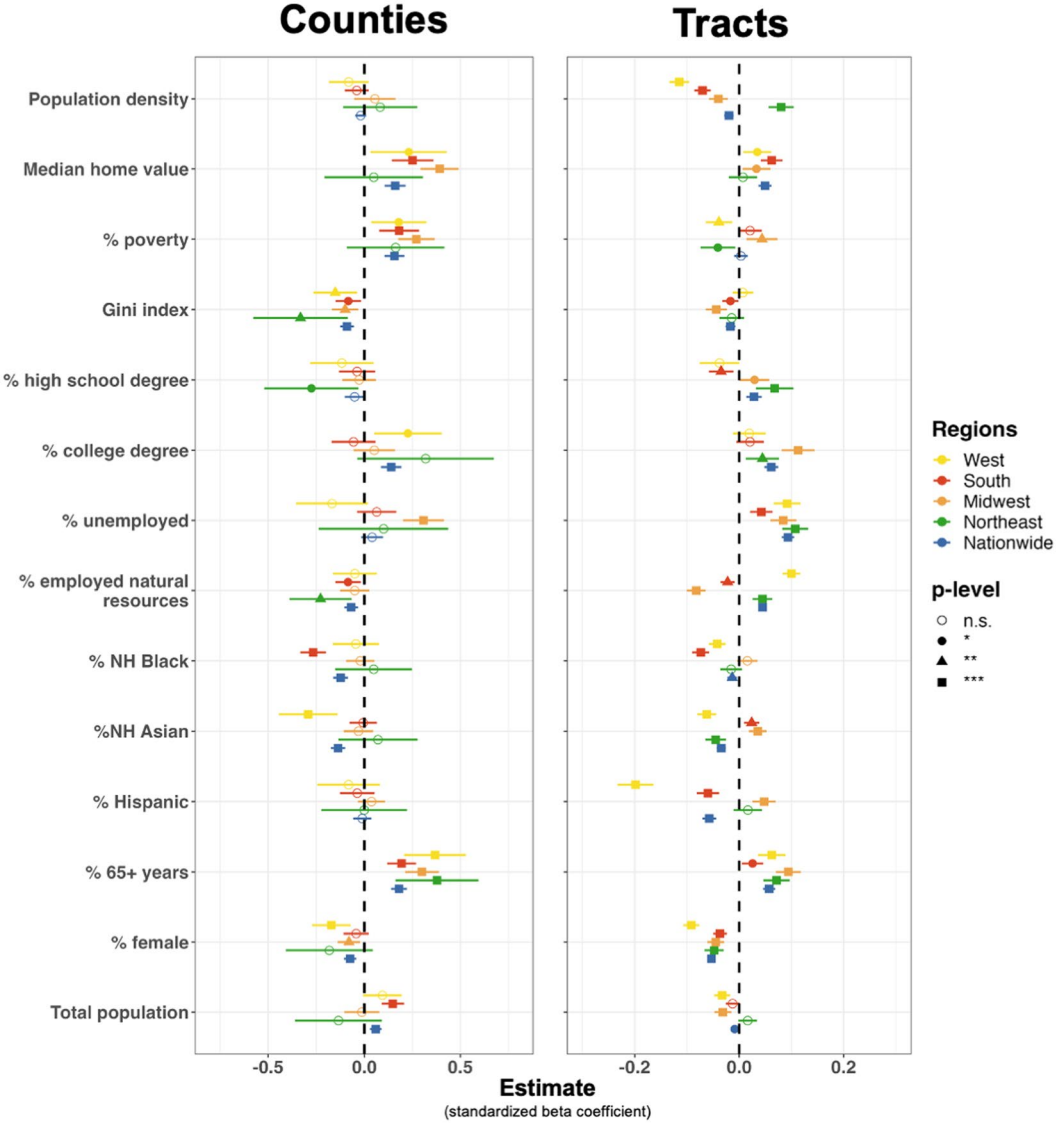
Regressing sociodemographic characteristics on the PAD-US-AR park cover dataset within counties and tracts. Notes: GLMMs with gamma distributions and U.S. state random effects, except for Midwestern counties, which report standard linear regression results. Standardized betas and 95% confidence intervals are shown. Differing symbols represent statistical significance (p-value): an empty circle is shown for *p* > 0.05, a filled-in circle for *p* < 0.05; a triangle for *p* < 0.01; a square for *p* < 0.001. X-axes are on different scales. Sensitivity models with median household income substituted for other socioeconomic variables are provided in Figure S9.

A listing of the sociodemographic characteristics considered in analyses is provided in Table S1. Descriptive statistics for each variable are presented in Tables S2–S6. Maps of the distribution of these variables are provided in Figure S8. Multivariate associations between the PAD-US-AR^48^ and sociodemographic characteristics are shown in Fig. 7 and Table S7. These results were derived from GLMMs with gamma distributions and U.S. states as random effects to account for the non-normal distribution of the outcome variable with minimal multicollinearity (Table S8).

Park cover was more strongly associated with sociodemographic characteristics at the county level than at the tract level. Around 30% of the variance in countywide park cover was explained in U.S. regions after accounting for state random effects (conditional R^2^_Northeast_ = 0.29, R^2^_Midwest_ = 0.31, R^2^_South_ = 0.23, R^2^_West_ = 0.38). Variance explained within counties across the country was over 60% (R^2^_Nationwide_ = 0.63). Variance explained at the tract level was closer to 10%–20% (R^2^_Nationwide_ = 0.19, R^2^_Northeast_ = 0.09, R^2^_Midwest_ = 0.08, R^2^_South_ = 0.12, R^2^_West_ = 0.18).

Three sociodemographic characteristics showed fairly consistent associations with park cover. On average, areas with greater shares of older adults (≥65 yrs) had more park cover. Areas with higher median home values also had more park cover, except in the Northeast. Last, areas with greater shares of female residents had less park cover on average, except in Northeastern and Southern counties. Two other sociodemographic characteristics showed consistent associations within either county or tract samples. First, counties with lower Gini index values (lower inequality) had more park cover on average. Secondly, tracts with higher unemployment rates had more park cover on average.

Associations between the PAD-US-AR and other sociodemographic characteristics varied by region. Park cover in Northeastern counties was concentrated in areas with lower rates of income inequality, high school graduation, and natural resource employment. Park cover in Midwestern counties was greater in areas with higher poverty rates, income inequality, and unemployment. Park cover in Southern counties was higher in areas with greater population densities or higher rates of poverty and lower rates of income inequality, natural resource employment, or non-Hispanic Black residents. Western counties showed greater park cover in areas with more poverty, higher shares of college degree holders, less income inequality, and lower shares of non-Hispanic Asian residents. Within tracts, park cover was higher in densely populated Northeastern areas but lower in densely populated areas throughout the rest of the country. Tract-level park cover was higher in areas with greater shares of residents employed in natural resource professions in the West and Northeast, while the opposite was found in the South and Midwest; in these areas, park cover was lower in areas where greater shares of people worked in natural resources professions. Park cover was higher in Midwestern and Southern tracts with greater shares of non-Hispanic Asians, whereas park cover was lower in Western and Northeastern tracts with greater shares of non-Hispanic Asians. In summary, park cover was associated with many sociodemographic characteristics, but the strength and direction varied by geography and unit of analysis.

Multivariate associations between the PAD-US-AR and sociodemographic characteristics in urban areas are presented in Table S10. In most cases, median home value continued to show strong positive associations with park cover. One exception was observed in Midwestern tracts, where median home value was negatively associated with park cover. Percent female no longer predicted park cover except in Southern tracts. Shares of older adults also predicted park cover in only a few urban cases; significant positive associations were observed only in nationwide and Northeastern tracts. Percent Non-Hispanic Asian residents emerged as a predictor in several models, but the direction of the associations differed. Nationwide models showed negative associations, while Midwestern counties and tracts and Southern tracts showed positive associations. County-level models of urban areas continued to predict the variance explained by park cover better than tract-level models of urban areas. Alternative models substituting median household income for other socio-economic indicators found mixed relationships between this variable and park cover (Table S9, Figure S9).

## Usage Notes

We present a new potential indicator of outdoor nature exposure for the contiguous U.S: the location of parks intended to be accessible for recreation. This dataset allows researchers to examine the number of outdoor recreation areas meant for public use around geographic units of interest (i.e., homes, neighborhoods, and transit routes). Other commonly-used metrics – like moderate/coarse resolution NDVI and tree canopy cover datasets – cannot identify whether the areas are managed for public recreational use. The PAD-US-AR^48^ is unique from these other metrics, as determined by the correlations presented above (Fig. 6).

The PAD-US-AR also differs in coverage from pre-existing park cover datasets. These differences were observed when tallying the geographic polygon units and calculating the total cover after dissolving all polygon units to account for some overlapping units. The reasons to utilize the PAD-US-AR dataset rather than these other options include the PAD-US-AR source data (PAD-US V2.1) being validated by the agencies managing the land, our systematic examination of what is accessible for recreation, and the clarity and transparency in its curation. The potential for park cover to not match park access for all residents in a county or tract remains high, as in any area-level exposure estimate^80–82^. Individual-level estimates should be calculated from the boundaries or centroids of park polygons along road or pedestrian networks when geolocated data for homes, schools, workplaces, or activity spaces are available.

The chances for residual confounding in area-level studies with the PAD-US-AR dataset exist if multivariate models do not control for sociodemographic characteristics of the areas encompassing parks. The PAD-US-AR has the most robust associations with home prices, shares of female residents, and shares of older presents. These should be statistically controlled in models using the PAD-US-AR as an independent variable or covariate. Other measures of socioeconomic status (i.e., median household income) might be insufficient to avoid residual confounding in ecological studies with PAD-US-AR data.

Since the PAD-US-AR was curated nationwide, it is most appropriate for use at larger geographic scales (i.e., regional and national). Studies focusing on smaller geographic contexts, such as within individual cities or states, should partner with local land management agencies and recreation departments to ensure PAD-US-AR data accurately represent all parks and protected areas managed for public outdoor recreation. Since ownership boundaries and land acquisitions can change annually, local land management agencies might also be able to identify new parks that aren’t present in the PAD-US-AR. Smaller-scale analyses may allow manual selection of land parcels with building footprints that occupy most of the area.

The PAD-US-AR may be best conceived as the minimum park coverage level. We excluded the approximately 35,000 areas covering over 42,000 km^2^ with unknown public access in the PAD-US. Some private parks, such as golf courses or community parks restricted to residents who pay homeowner association fees, can provide opportunities for outdoor recreation that activate the same health-promoting pathways as public parks. People living in the counties and tracts presented in the datasets may have more access to outdoor recreational opportunities than suggested by the PAD-US-AR.

As the nature-health literature expands, exposure estimates are expected to develop and be refined. The PAD-US-AR presents a significant advancement in this body of literature by offering researchers an assessment of where parks are available for outdoor recreation.

## Supplementary information

Supplementary Information

## Data Availability

R (4.1.2) was used to generate the PAD-US-AR^48^ dataset and results. QGIS (3.18.3) was used to create the maps. Scripts and source data to reproduce results are available on OSF (https://osf.io/pwdsg/).
