# Supplementary material for: The PAD-US-AR dataset: Measuring accessible and recreational parks in the contiguous United States
Source: Sci Data. 2022 Dec 16;9:773. doi: 10.1038/s41597-022-01857-7 (PMC9758140; doi:10.1038/s41597-022-01857-7)
Supplement: Supplementary file 1 — Supplementary Information [file 41597_2022_1857_MOESM1_ESM.docx]

**SUPPLEMENTAL RESOURCES**

**A United States dataset of accessible and recreational parks
with greenspace and sociodemographic comparisons**

Matthew H. E. M. Browning^1,2*^, Alessandro Rigolon^3^, Scott Ogletree^4^, Ruoyu Wang^5^, Jochem O. Klompmaker^6,7^, Chris Bailey^2^, Ryan Gagnon^1^, Peter James^6,8^

^1^ Department of Parks, Recreation and Tourism Management, Clemson University, Clemson, South Carolina 29634, USA

^2^ NatureQuant LLC, [www.naturequant.com](http://www.naturequant.com), Bend, OR, USA

^3^ Department of City and Metropolitan Planning, The University of Utah, 375 South 1530 East, Salt Lake City, Utah 84112, USA

^4^ OPENspace Research Centre, School of Architecture and Landscape Architecture, University of Edinburgh, 74 Lauriston Place, Edinburgh, UK EH3 9DF

^5^ Centre for Public Health, Block A, Royal Victoria Hospital, Queen's University Belfast, Belfast, Northern Ireland, United Kingdom

^6^ Department of Environmental Health, Harvard T. H. Chan School of Public Health, 655 Huntington Avenue, Boston, Massachusetts 02115, USA

^7^ Channing Division of Network Medicine, Department of Medicine, Brigham and Women’s Hospital, 181 Longwood Avenue, Boston, Massachusetts 02115, USA

^8^ Department of Population Medicine, Harvard Medical School and Harvard Pilgrim Health Care Institute, 401 Park Drive, Boston, Massachusetts 02215, USA

^*^ Corresponding author: [mhb2@clemson.edu](mailto:mhb2@clemson.edu)


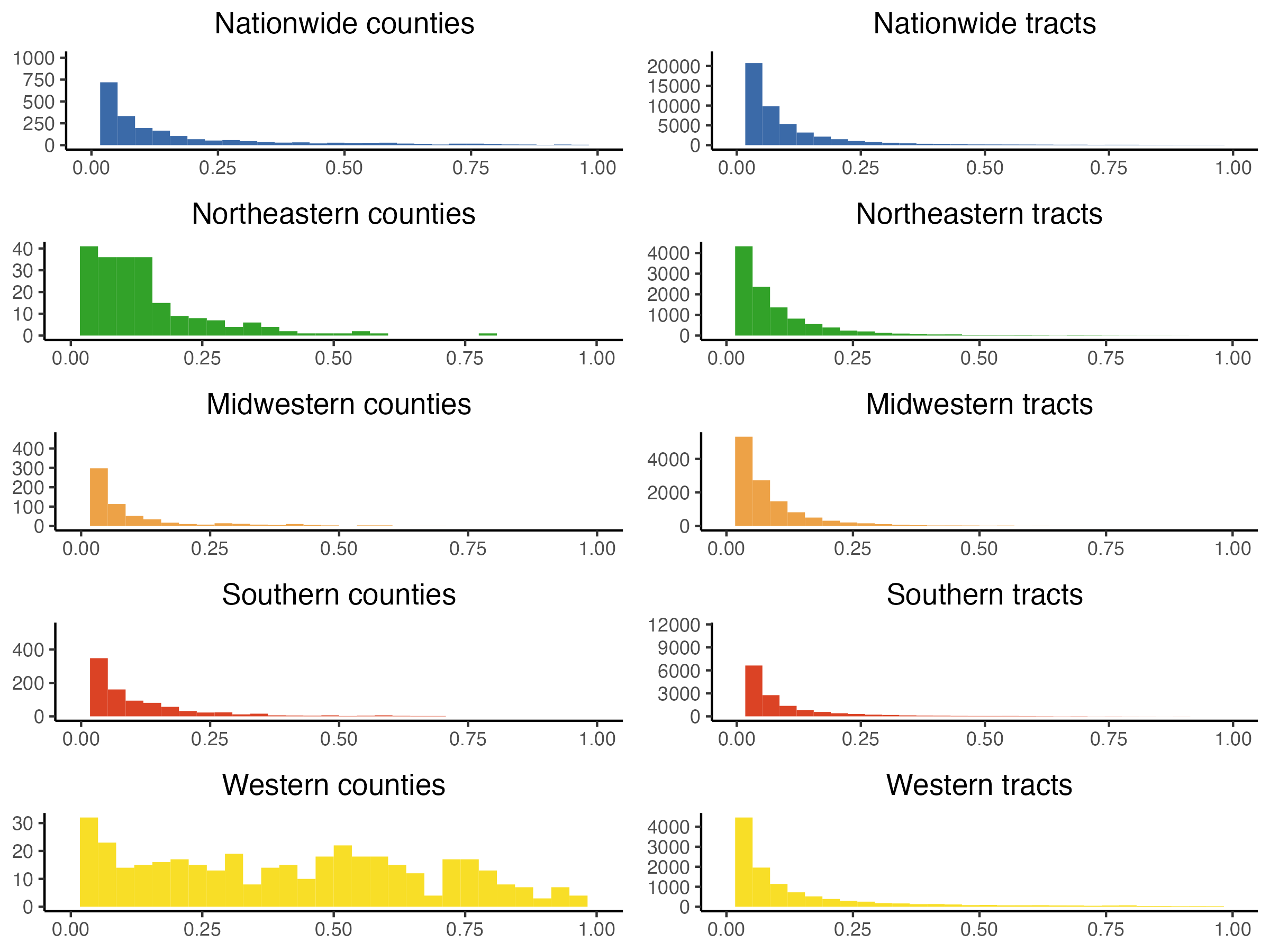


**Figure S1**. Histograms of county (left) and tract (right) level PAD-US-AR park cover estimates across the continental U.S.

**
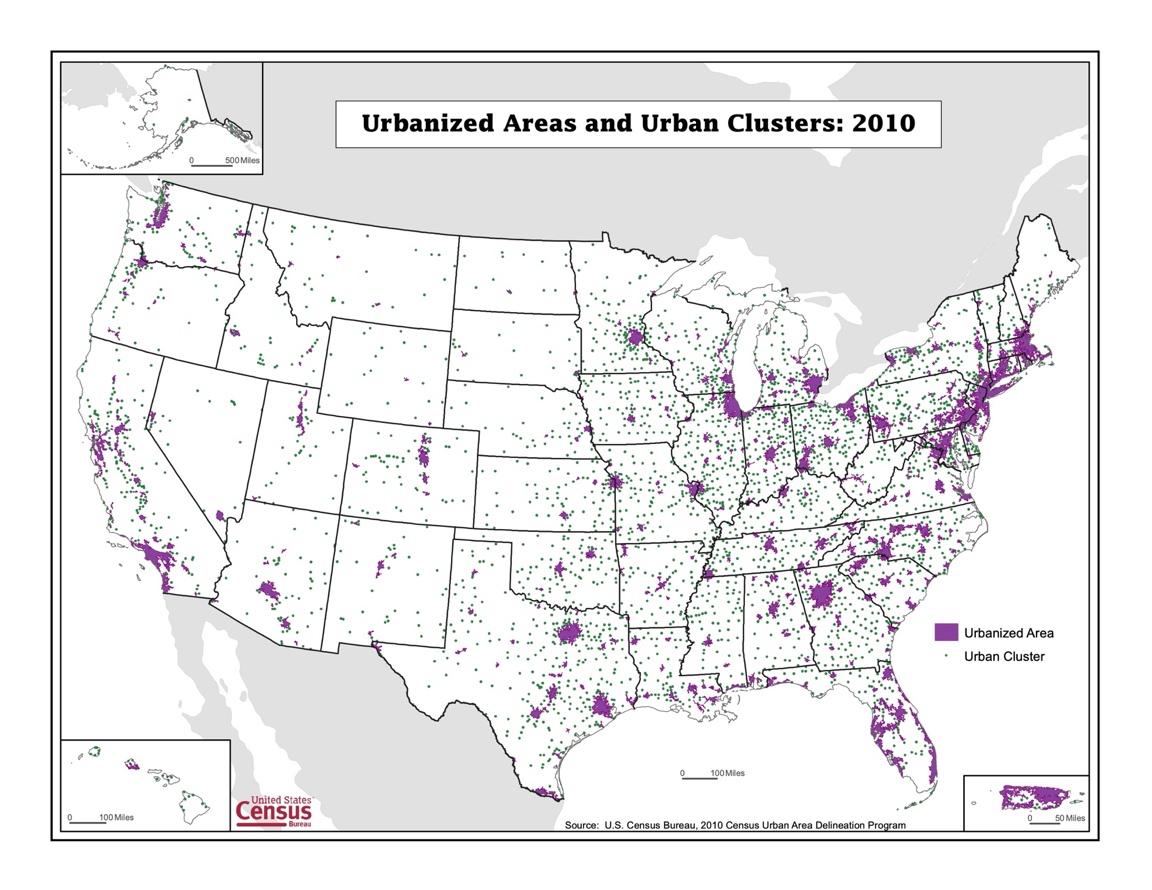

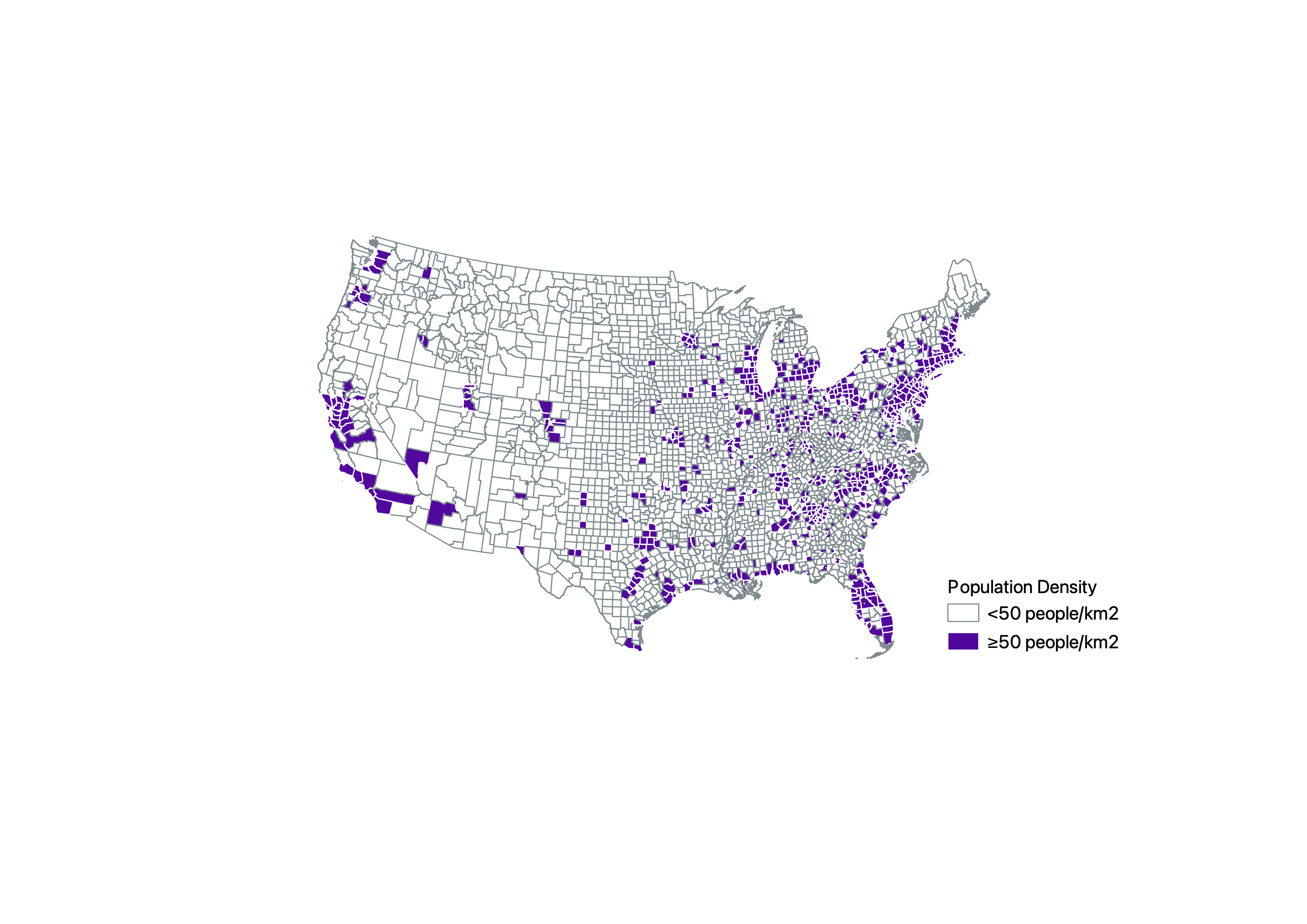
**

**Figure S2**. Comparisons between U.S. Census urbanized areas circa 2010 (N=497) and counties with population densities ≥50 people/km^2^. Census map retrieved from: https://www.census.gov/library/visualizations/2010/geo/ua2010_uas_and_ucs_map.html

**
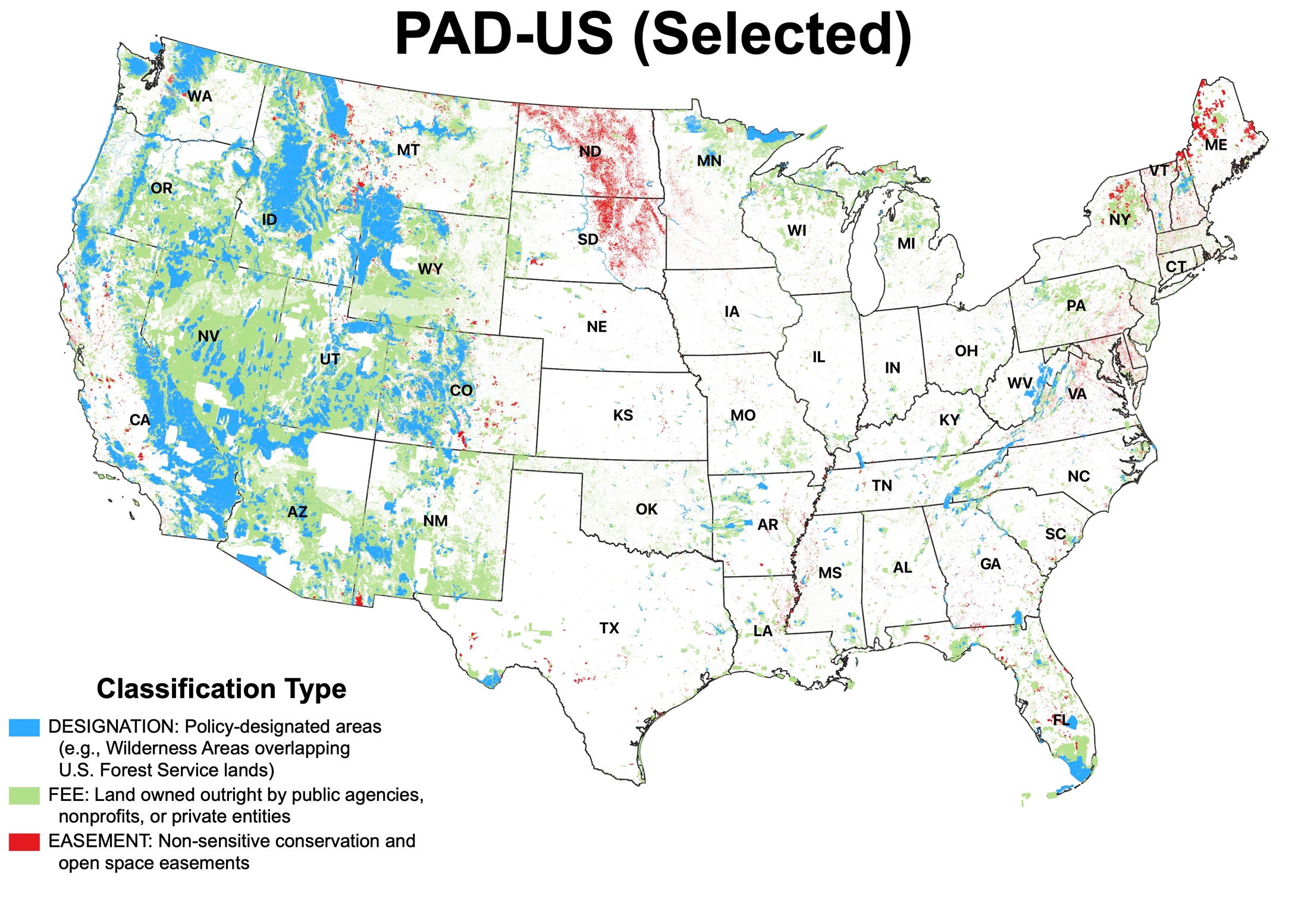

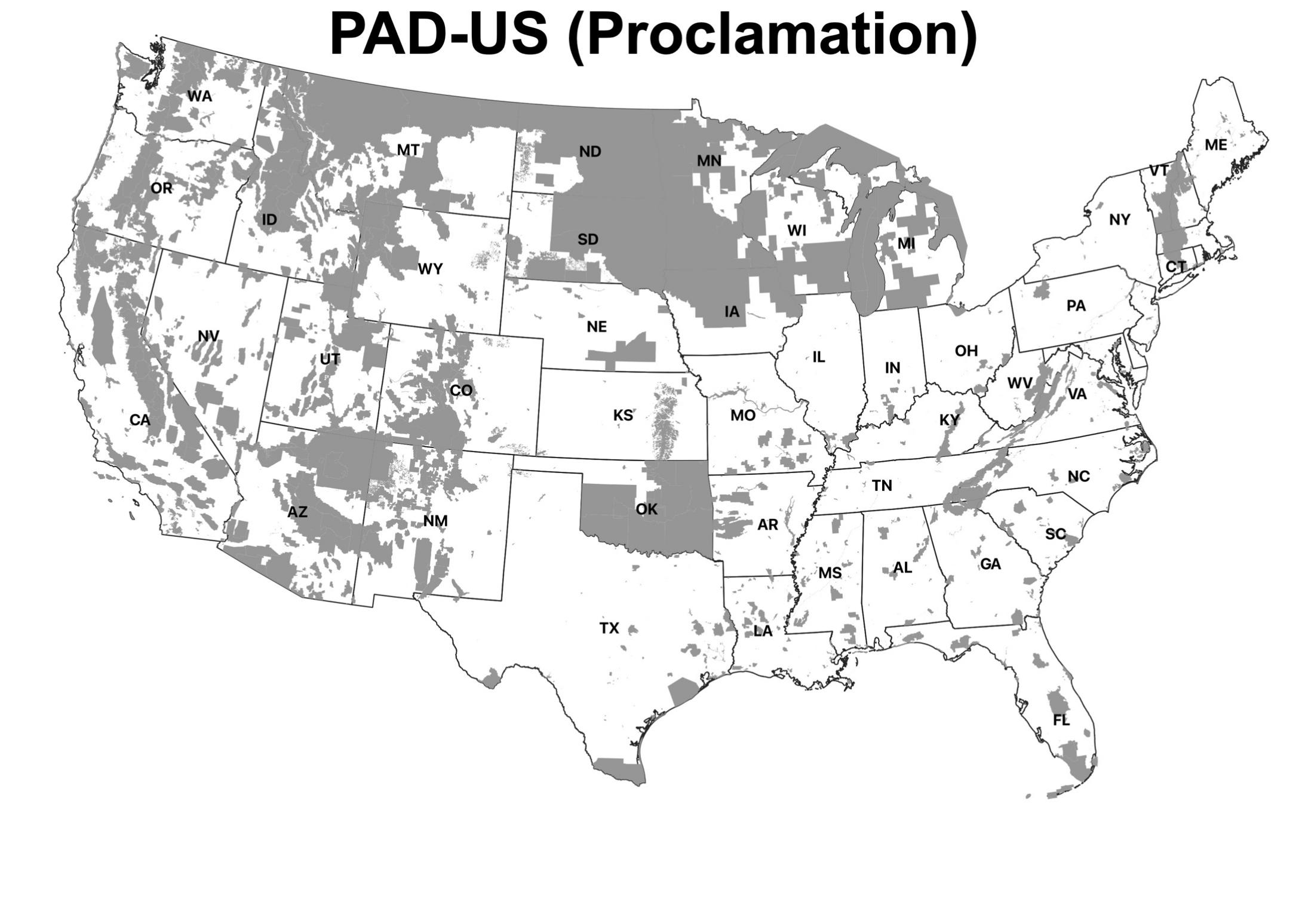

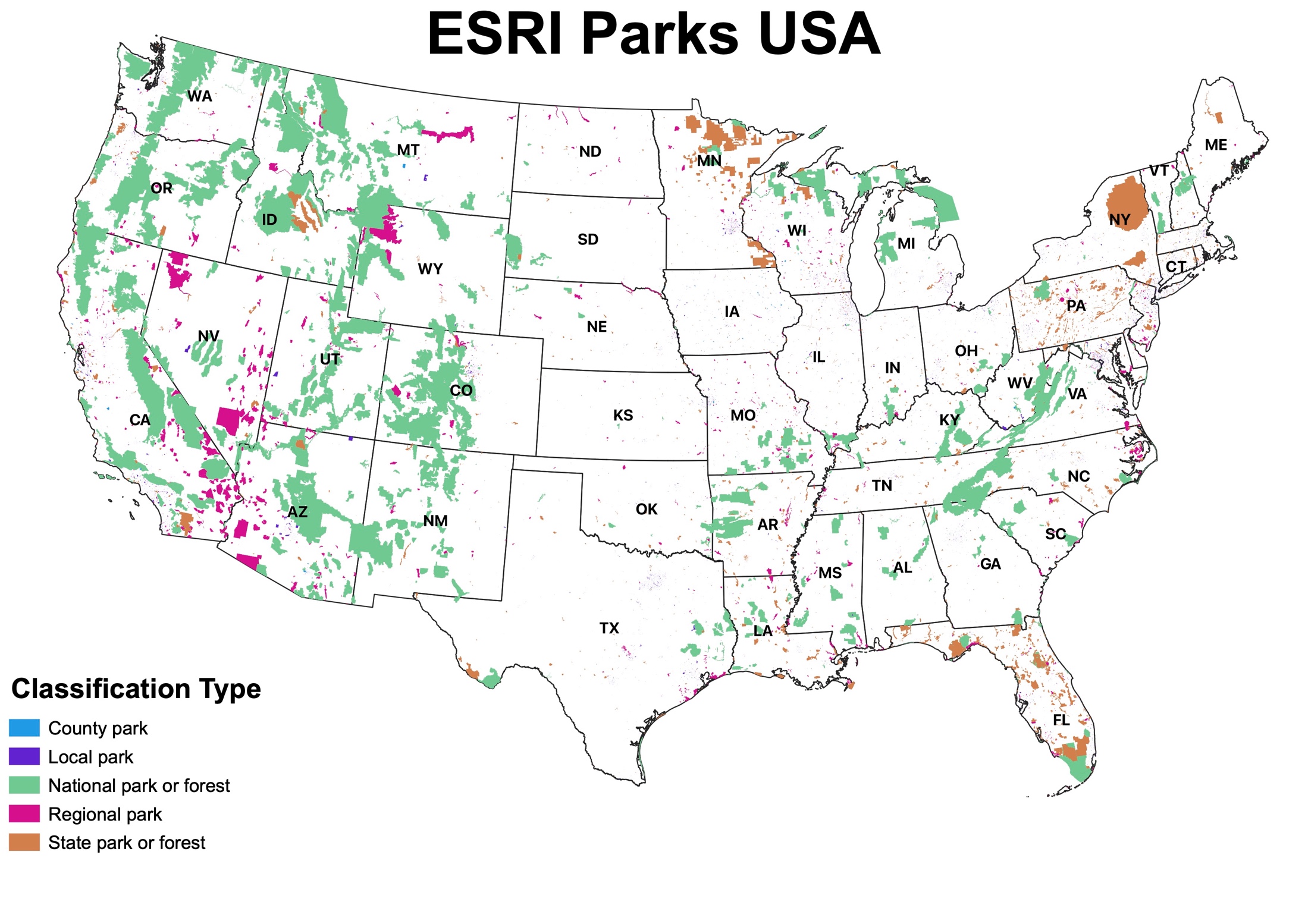

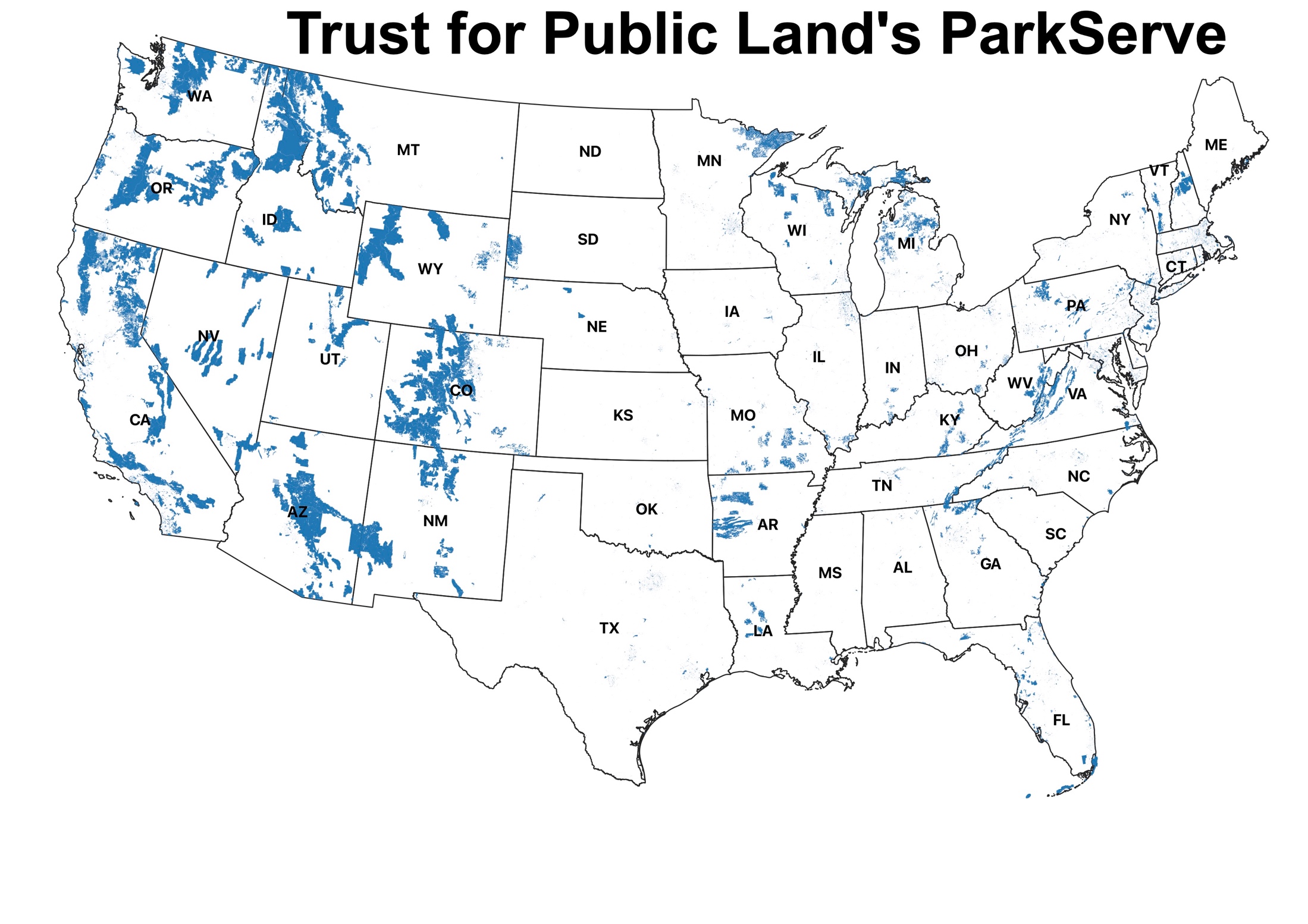

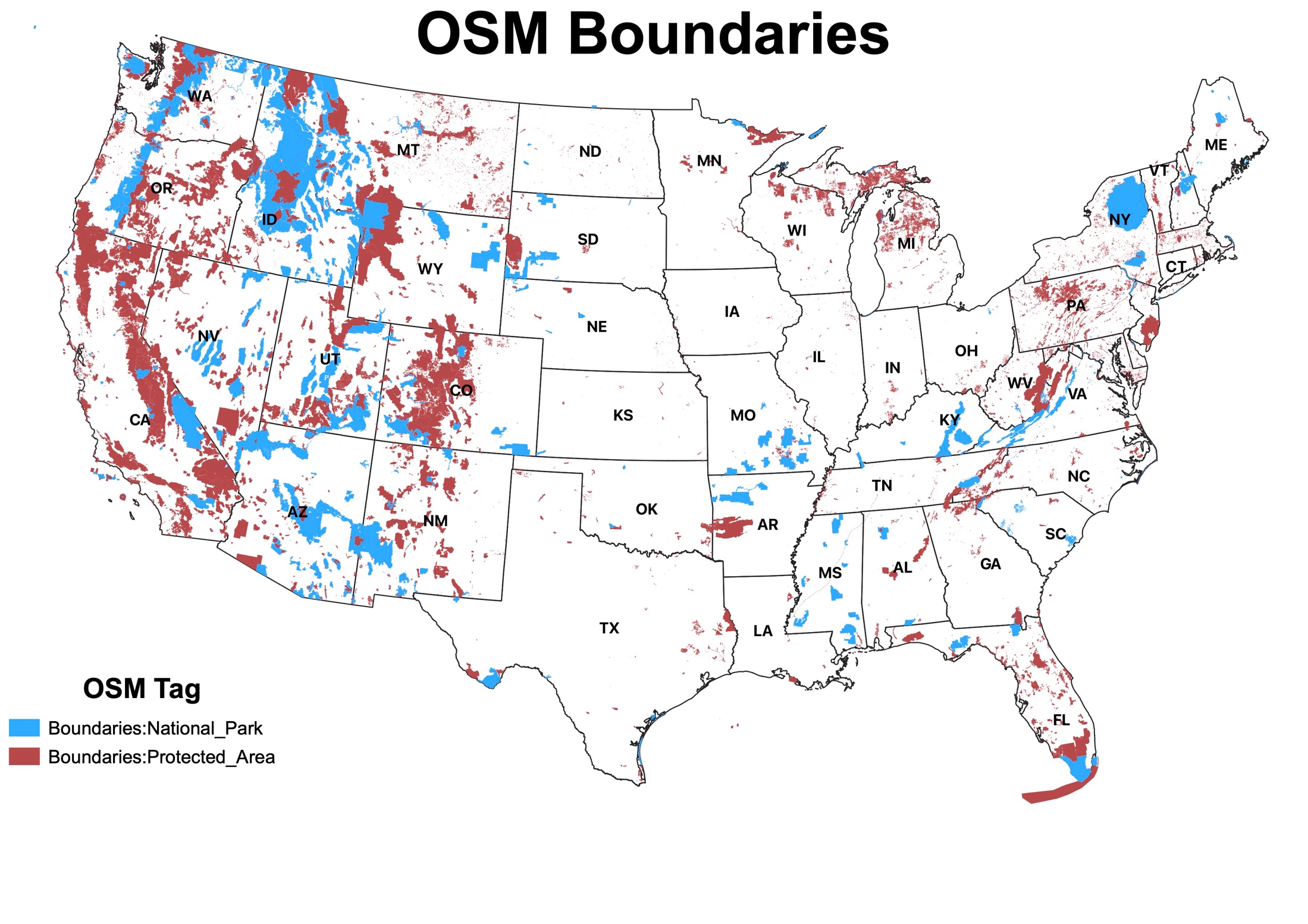

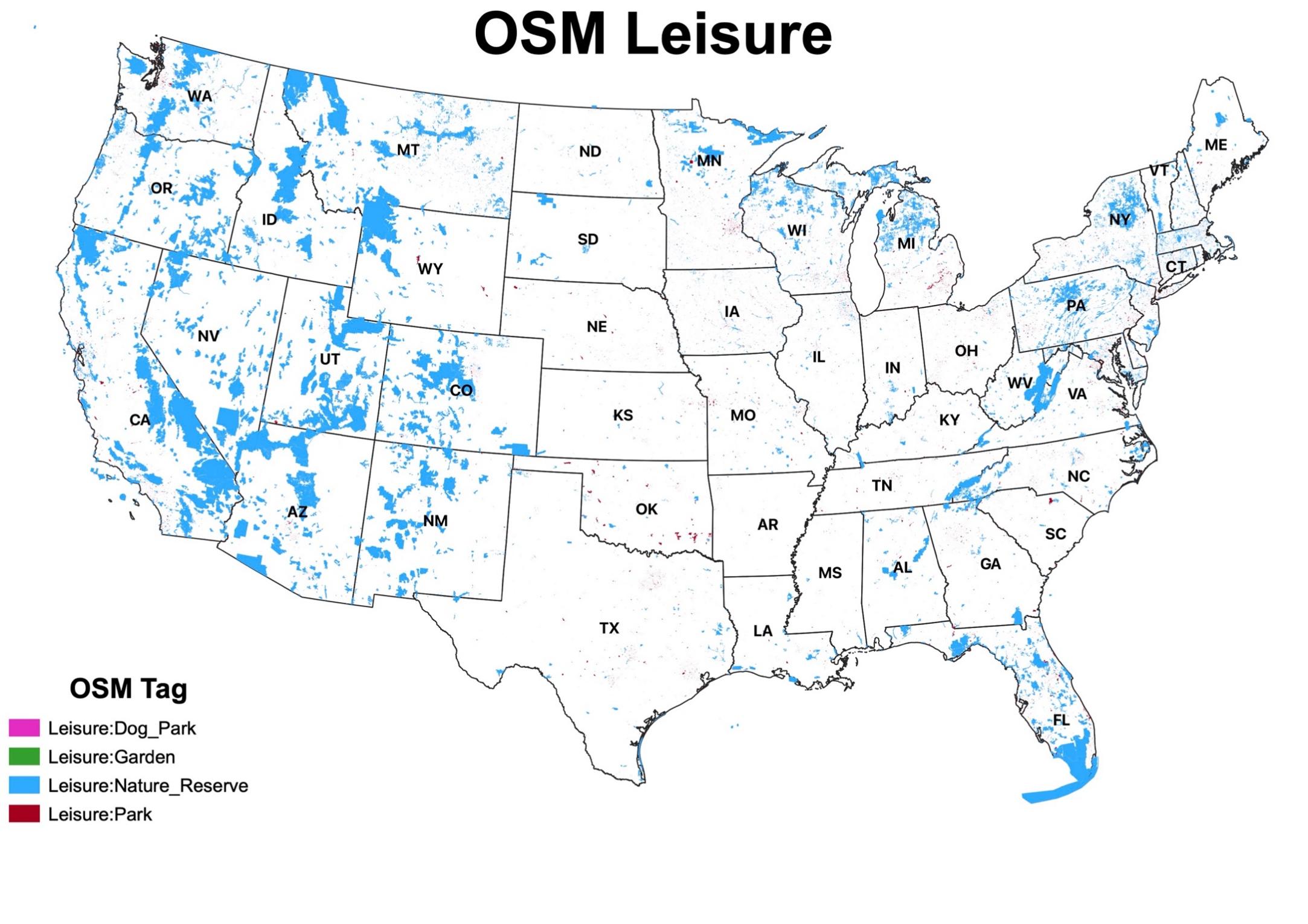
**

**Figure S3.** Preceding pages include nationwide comparisons between park cover datasets.

**
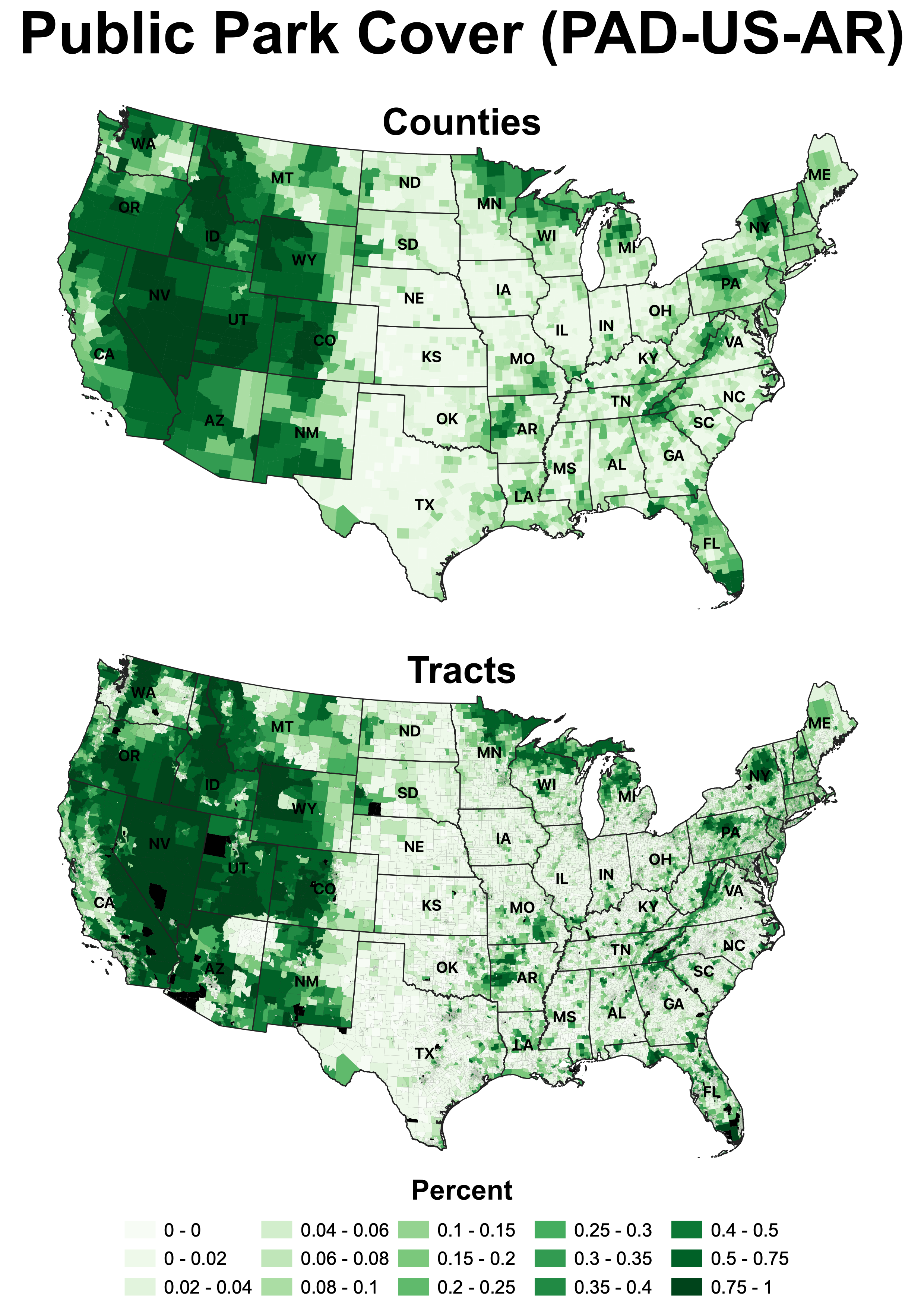
**

**
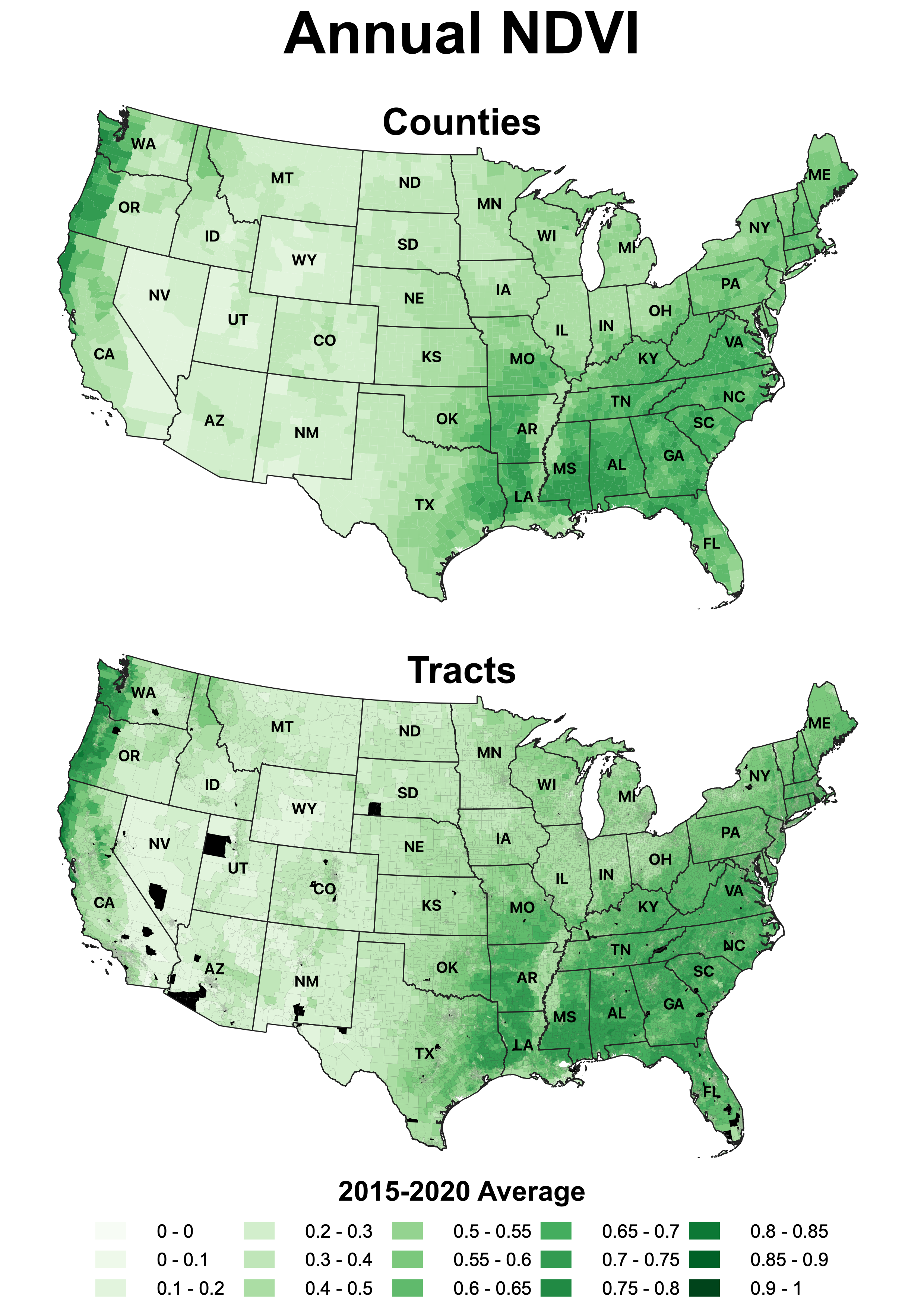

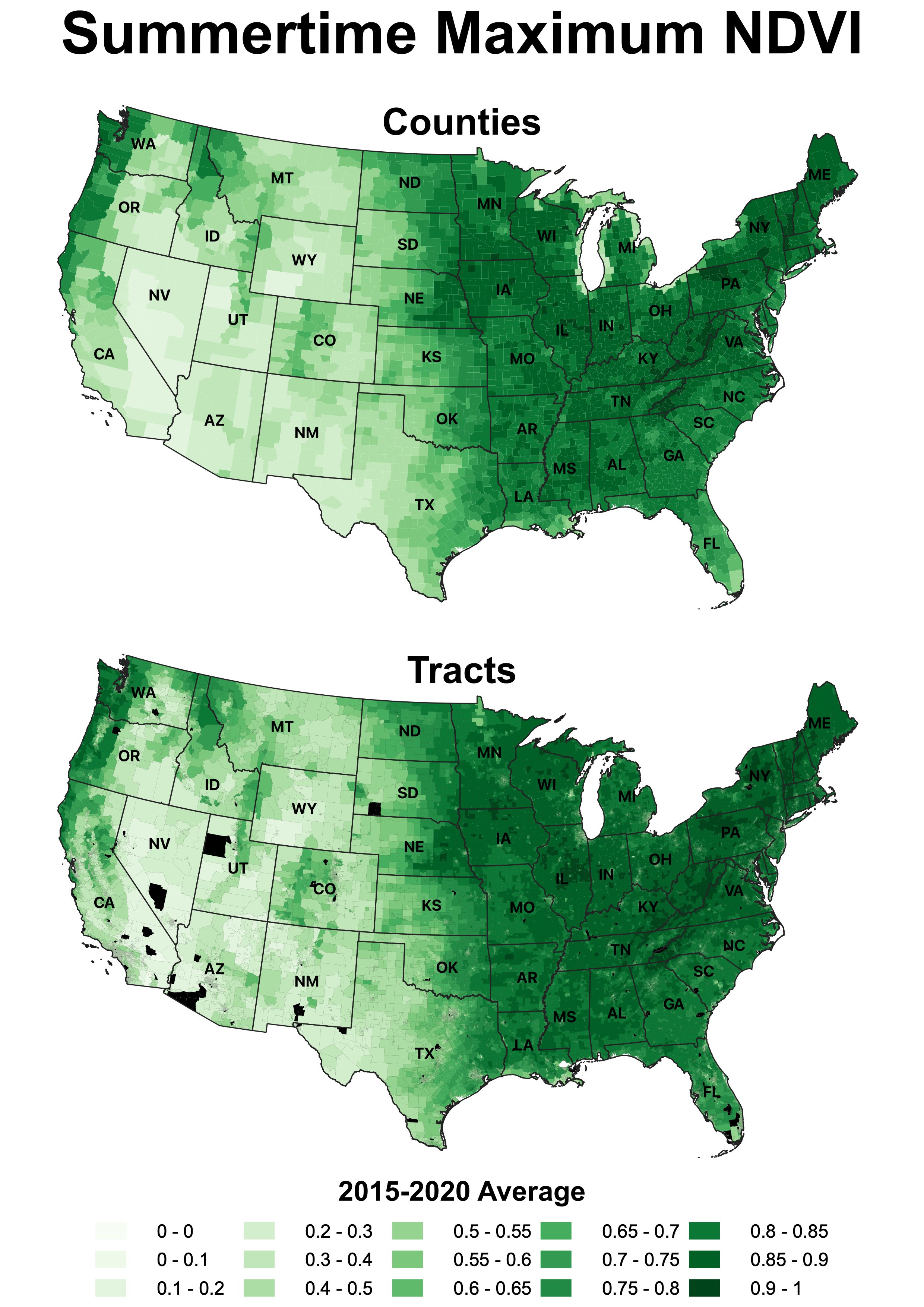

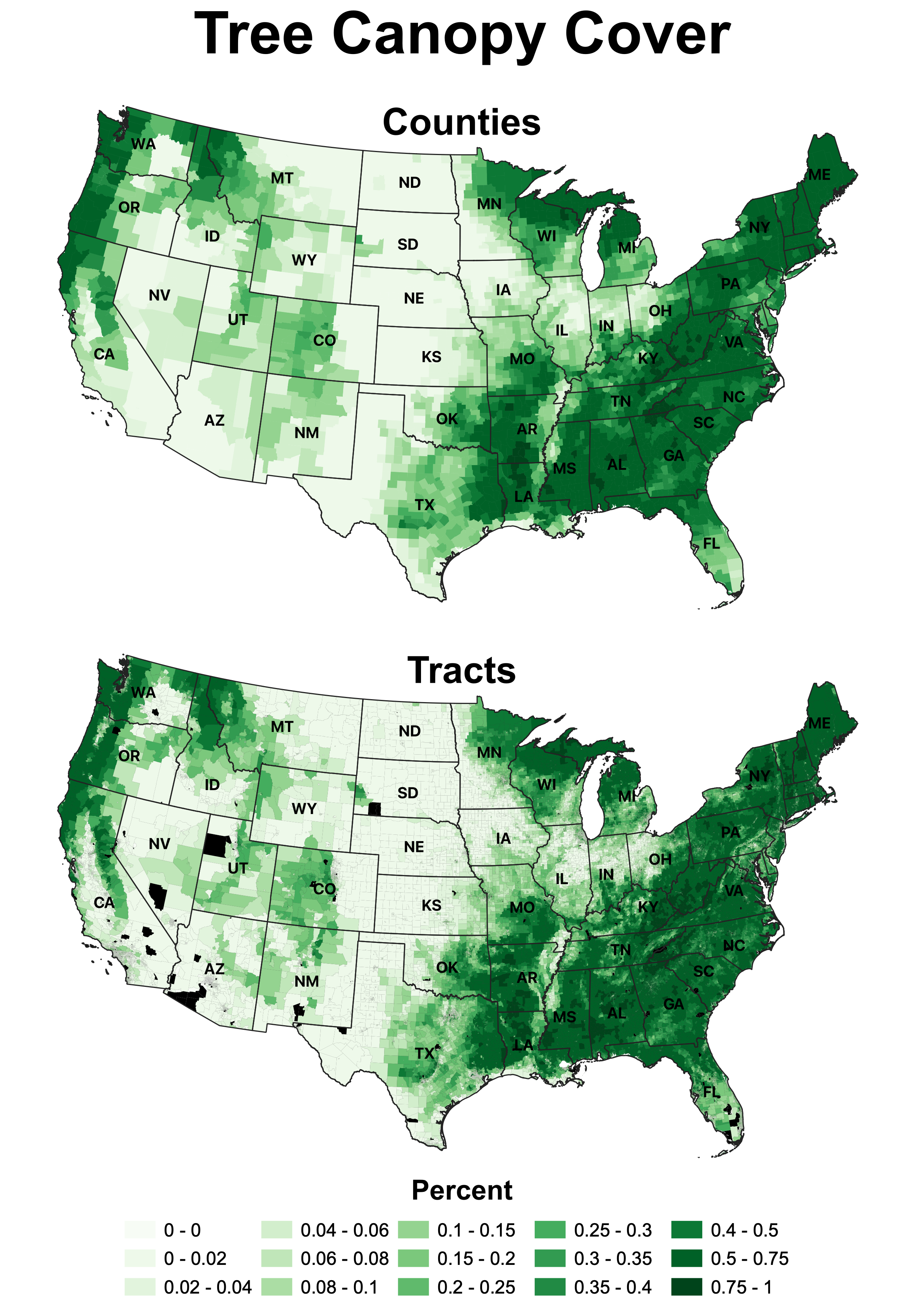
**

**Figure S4**. Maps of public park cover and other green space measures in counties and tracts across the U.S.

Note: Tracts with complete data shown (N=70,580). Missing tracts are presented in black.


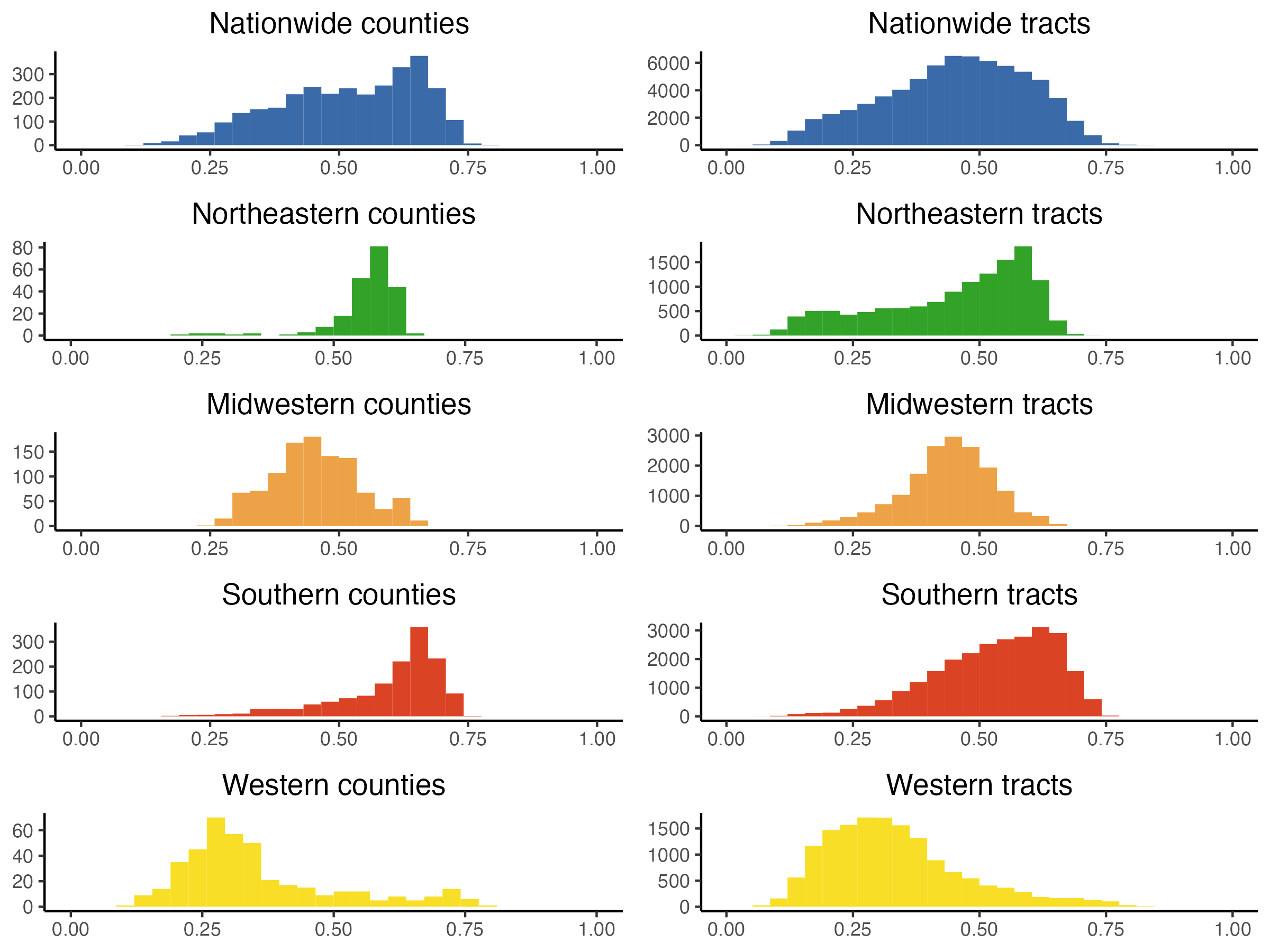


**Figure S5**. Histograms of county-level and tract-level **NDVI annual averages** across the continental U.S. and within census regions. Note: X and Y-axis ranges vary between plots.

**
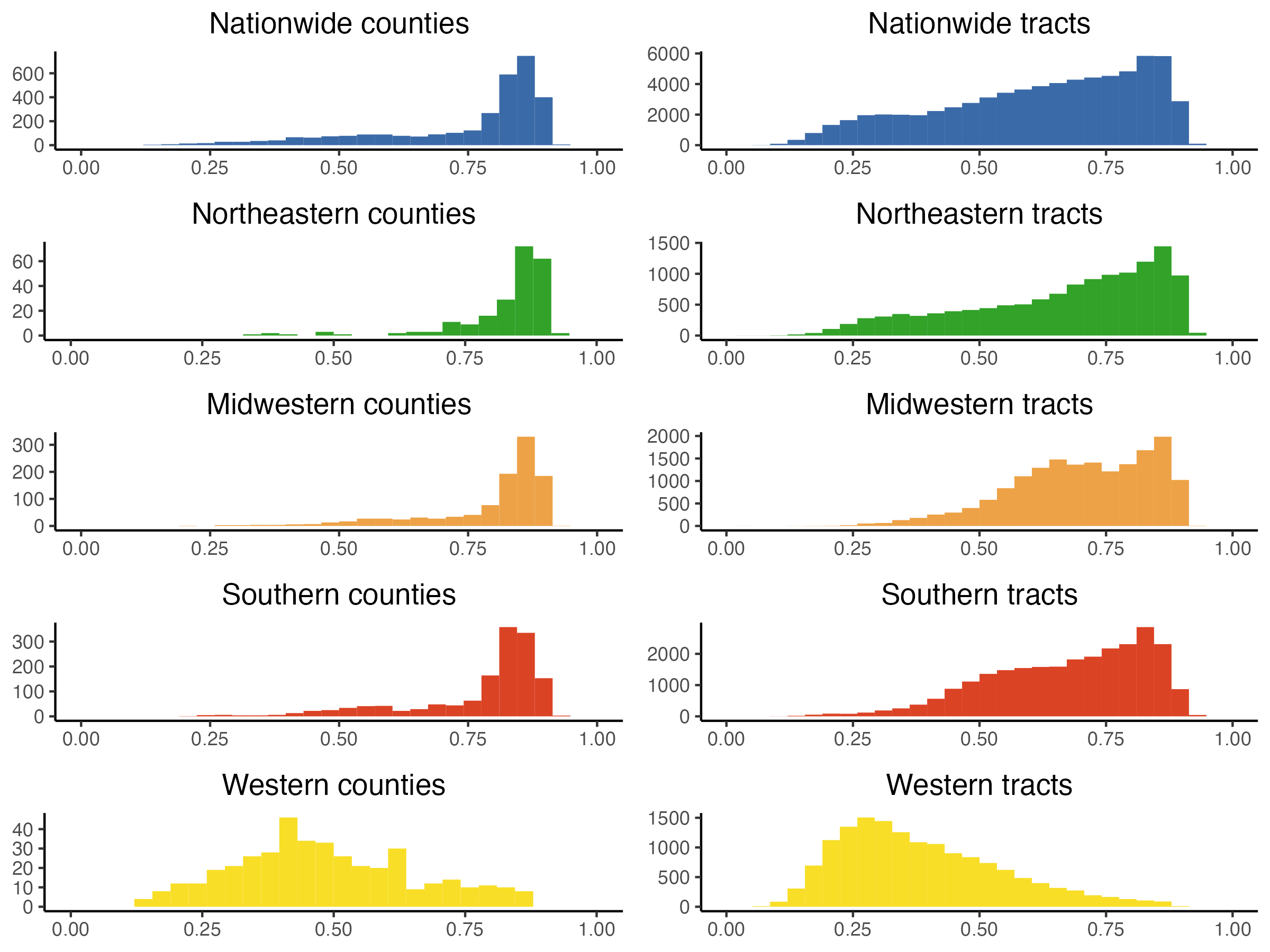
**

**Figure S6**. Histograms of county-level and tract-level **NDVI summertime maximums** across the continental U.S. and within census regions. Note: X and Y-axis ranges vary between plots.


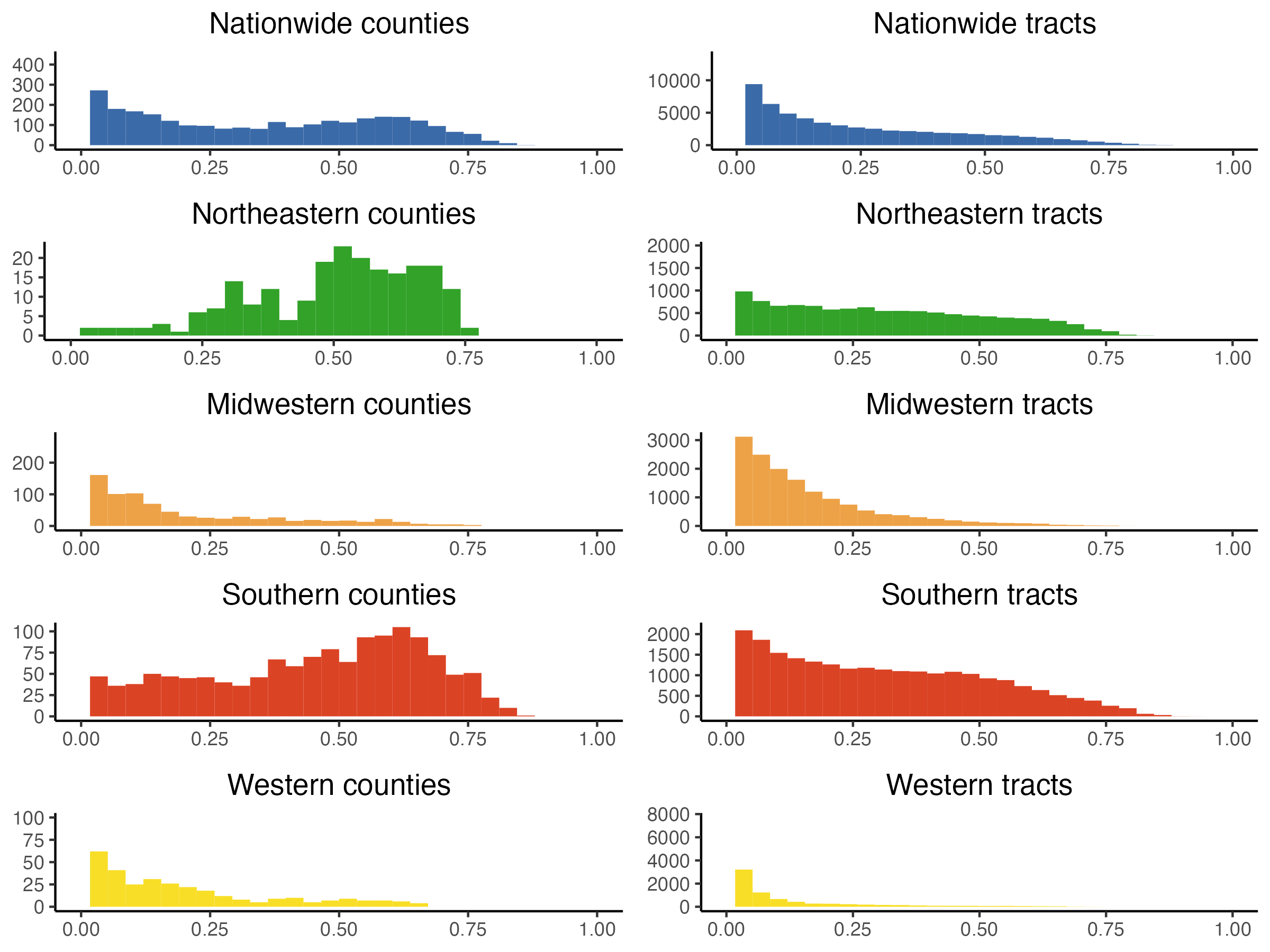


**Figure S7**. Histograms of county-level and tract-level **tree canopy cover** across the continental U.S. and within census regions. Note: X and Y-axis ranges vary between plots.

**Table S1.** Sociodemographic characteristics examined in this study.

| **Variable Name** | **Description** | **Source** |
| --- | --- | --- |
| Population density | Number of people per km^2^ | American Community Survey (ACS) 5-Year Data: 2015-2019 |
| Median home value | Median value (USD) of owner-occupied housing units | ACS 2015-2019 |
| % poverty | Ratio of households with incomes below the poverty level in the past 12 months to total number of households | ACS 2015-2019 |
| GINI index | Gini index of income inequality, ranging from 0 (perfect equality, where everyone receives an equal share) to 1 (perfect inequality, where only one recipient or group receives all the income) | ACS 2015-2019 |
| % high school degree | Ratio of high school diploma earners (or greater levels of educational achievement) among people 25 years or over to total population 25 years or over | ACS 2015-2019 |
| % college degree | Ratio of Bachelor’s degree holders (or greater levels of educational achievement) among people 25 years or over to total population 25 years or over | ACS 2015-2019 |
| % unemployed | Ratio of people aged 16 years or over in the labor force to total population aged 16 years or over | ACS 2015-2019 |
| % employed natural resources | Ratio of people aged 16 years or over in agriculture, forestry, fishing and hunting, and mining industries to total population aged 16 years or over | ACS 2015-2019 |
| % NH Black | Ratio of population who are not Hispanic or Latino and Black or African American to total population | ACS 2015-2019 |
| % NH Asian | Ratio of population who are not Hispanic or Latino and Asian to total population | ACS 2015-2019 |
| % Hispanic | Ratio of Hispanic or Latino Origin by Race to Total Population | ACS 2015-2019 |
| % 65+ years | Ratio of population who are 65 years or over to total population | ACS 2015-2019 |
| % Female | Ratio of population who are female to total population | ACS 2015-2019 |
| Total population | Population size (number of residents) | ACS 2015-2019 |

**Table S2.** Descriptive statistics for socio-demographic factors (**Nationwide**)

|  | **Counties**  (N=3108) | | | **Tracts**  (N=70580) | | |
| --- | --- | --- | --- | --- | --- | --- |
|  | *Med* | *IQR* | *Range* | *Med* | IQR | *Range* |
| Population density | 17.8 | 39.4 | 27954  (0.04-27954) | 876.5 | 1950.1 | 99783  (0.03-99783) |
| Median household income | 51658 | 15460 | 120795  (21504-142299) | 59780 | 37276 | 245872  (4129-250001) |
| Median home value | 127200 | 76300 | 1073400  (24400-1097800) | 192300 | 214300 | 1990002  (9999-2000001) |
| % poverty | 0.14 | 0.07 | 0.46 (0.02-0.48) | 0.11 | 0.12 | 0.8 (0-0.8) |
| Gini index | 0.44 | 0.05 | 0.39 (0.32-0.71) | 0.42 | 0.08 | 0.77 (0.05-0.82) |
| % high school degree | 0.88 | 0.08 | 0.72 (0.26-0.99) | 0.9 | 0.12 | 0.76 (0.24-1) |
| % college degree | 0.20 | 0.11 | 0.78 (0-0.78) | 0.26 | 0.26 | 0.97 (0-0.97) |
| % unemployed | 0.41 | 0.11 | 0.65 (0.2-0.85) | 0.36 | 0.12 | 0.96 (0.03-0.99) |
| % employed natural resources | 0.04 | 0.07 | 0.60 (0-0.60) | 0.01 | 0.02 | 0.67 (0-0.67) |
| % NH Black | 0.02 | 0.09 | 0.87 (0-0.87) | 0.04 | 0.14 | 1 (0-1) |
| % NH Asian | 0.01 | 0.01 | 0.36 (0-0.36) | 0.02 | 0.05 | 0.94 (0-0.94) |
| % Hispanic | 0.04 | 0.08 | 0.99 (0-0.99) | 0.08 | 0.18 | 1 (0-1) |
| % female | 0.50 | 0.02 | 0.3 (0.27-0.57) | 0.51 | 0.04 | 0.94 (0.01-0.95) |
| % 65+ years | 0.18 | 0.05 | 0.54 (0.03-0.57) | 0.16 | 0.09 | 0.92 (0-0.92) |
| Total population | 25946 | 56714 | 10081472  (98-10081570) | 4166 | 2618 | 72004  (34-72041) |

**Table S3.** Descriptive statistics for socio-demographic factors (**Northeast**)

|  | **Counties**  (N=217) | | | **Tracts**  (N=13022) | | |
| --- | --- | --- | --- | --- | --- | --- |
|  | *Med* | *IQR* | *Range* | *Med* | IQR | *Range* |
| Population density | 63.6 | 167.4 | 27953 (0.93-27954) | 1270.5 | 4064.6 | 84508  (0.07-84508) |
| Median household income | 60240 | 18518 | 76383 (39717-116100) | 69456 | 42570 | 239208 (10793-250001) |
| Median home value | 177100 | 118100 | 1008300  (76400-1084700) | 268000 | 282075 | 1990002  (9999-2000001) |
| % poverty | 0.12 | 0.04 | 0.24 (0.04-0.28) | 0.09 | 0.11 | 0.72 (0-0.72) |
| Gini index | 0.44 | 0.03 | 0.2 (0.39-0.6) | 0.42 | 0.08 | 0.52 (0.25-0.77) |
| % high school degree | 0.91 | 0.04 | 0.23 (0.73-0.96) | 0.92 | 0.1 | 0.68 (0.32-1) |
| % college degree | 0.28 | 0.15 | 0.53 (0.08-0.61) | 0.32 | 0.28 | 0.96 (0-0.97) |
| % unemployed | 0.38 | 0.07 | 0.58 (0.27-0.85) | 0.35 | 0.1 | 0.88 (0.05-0.93) |
| % employed natural resources | 0.02 | 0.02 | 0.12 (0-0.12) | 0 | 0.01 | 0.31 (0-0.31) |
| % NH Black | 0.03 | 0.05 | 0.41 (0-0.41) | 0.03 | 0.11 | 0.99 (0-0.99) |
| % NH Asian | 0.01 | 0.03 | 0.25 (0-0.25) | 0.02 | 0.06 | 0.92 (0-0.92) |
| % Hispanic | 0.03 | 0.06 | 0.55 (0.01-0.56) | 0.06 | 0.14 | 0.97 (0-0.97) |
| % female | 0.51 | 0.01 | 0.26 (0.27-0.53) | 0.51 | 0.04 | 0.79 (0.16-0.95) |
| % 65+ years | 0.19 | 0.04 | 0.19 (0.12-0.3) | 0.17 | 0.08 | 0.92 (0-0.92) |
| Total population | 102642 | 245889 | 2585459  (4515-2589974) | 3981 | 2424 | 28011  (98-28109) |

**Table S4.** Descriptive statistics for socio-demographic factors (**Midwest**)

|  | **Counties**  (N=1055) | | | **Tracts**  (N=16762) | | |
| --- | --- | --- | --- | --- | --- | --- |
|  | *Med* | *IQR* | *Range* | *Med* | IQR | *Range* |
| Population density | 12.74 | 28.0 | 2098.7  (0.19-2098.8) | 652.7 | 1501.2 | 99783.2  (0.14-99783.4) |
| Median household income | 54191 | 11082 | 82577 (24331-106908) | 57395 | 29431 | 245872  (4129-250001) |
| Median home value | 118400 | 54600 | 287200  (26000-313200) | 145750 | 107900 | 1538501  (9999-1548500) |
| % poverty | 0.12 | 0.05 | 0.46 (0.02-0.48) | 0.11 | 0.11 | 0.8 (0-0.8) |
| Gini index | 0.43 | 0.04 | 0.22 (0.33-0.56) | 0.41 | 0.07 | 0.71 (0.11-0.82) |
| % high school degree | 0.91 | 0.04 | 0.41 (0.57-0.98) | 0.92 | 0.08 | 0.72 (0.28-1) |
| % college degree | 0.2 | 0.08 | 0.51 (0.09-0.59) | 0.23 | 0.22 | 0.96 (0-0.96) |
| % unemployed | 0.37 | 0.08 | 0.4 (0.24-0.64) | 0.35 | 0.11 | 0.92 (0.08-0.99) |
| % employed natural resources | 0.05 | 0.08 | 0.51 (0-0.51) | 0.01 | 0.02 | 0.55 (0-0.55) |
| % NH Black | 0.01 | 0.02 | 0.46 (0-0.46) | 0.03 | 0.1 | 1 (0-1) |
| % NH Asian | 0.01 | 0.01 | 0.14 (0-0.14) | 0.01 | 0.03 | 0.86 (0-0.86) |
| % Hispanic | 0.03 | 0.03 | 0.61 (0-0.61) | 0.04 | 0.06 | 0.99 (0-0.99) |
| % female | 0.5 | 0.01 | 0.19 (0.37-0.55) | 0.51 | 0.04 | 0.67 (0.03-0.7) |
| % 65+ years | 0.19 | 0.05 | 0.29 (0.07-0.36) | 0.16 | 0.08 | 0.57 (0-0.57) |
| Total population | 19941 | 36794.5 | 5197880  (395-5198275) | 3717 | 2317 | 46382  (100-46482) |

**Table S5.** Descriptive statistics for socio-demographic factors (**South**)

|  | **Counties**  (N=1422) | | | **Tracts**  (N=25601) | | |
| --- | --- | --- | --- | --- | --- | --- |
|  | *Med* | *IQR* | *Range* | *Med* | IQR | *Range* |
| Population density | 21.8 | 44.1 | 4383.9  (0.04-4384) | 575.1 | 1353.0 | 30886.5  (0.1-30887) |
| Median household income | 46432 | 14309 | 120795 (21504-142299) | 53158 | 32623 | 241095 (8906-250001) |
| Median home value | 116400 | 70300 | 764900  (24400-789300) | 156800 | 139300 | 1990002  (9999-2000001) |
| % poverty | 0.17 | 0.08 | 0.4 (0.03-0.43) | 0.13 | 0.13 | 0.79 (0-0.79) |
| Gini index | 0.46 | 0.04 | 0.39 (0.32-0.71) | 0.43 | 0.08 | 0.73 (0.05-0.79) |
| % high school degree | 0.84 | 0.08 | 0.72 (0.26-0.99) | 0.88 | 0.13 | 0.75 (0.25-1) |
| % college degree | 0.17 | 0.1 | 0.78 (0-0.78) | 0.23 | 0.25 | 0.95 (0-0.95) |
| % unemployed | 0.45 | 0.1 | 0.58 (0.2-0.78) | 0.38 | 0.14 | 0.96 (0.03-0.99) |
| % employed natural resources | 0.03 | 0.05 | 0.51 (0-0.51) | 0.01 | 0.03 | 0.51 (0-0.51) |
| % NH Black | 0.09 | 0.24 | 0.87 (0-0.87) | 0.1 | 0.25 | 1 (0-1) |
| % NH Asian | 0.01 | 0.01 | 0.2 (0-0.2) | 0.01 | 0.03 | 0.76 (0-0.76) |
| % Hispanic | 0.05 | 0.09 | 0.99 (0-0.99) | 0.07 | 0.17 | 1 (0-1) |
| % female | 0.51 | 0.02 | 0.24 (0.33-0.57) | 0.51 | 0.04 | 0.79 (0.01-0.8) |
| % 65+ years | 0.18 | 0.05 | 0.54 (0.03-0.57) | 0.16 | 0.09 | 0.89 (0.01-0.9) |
| Total population | 26241.5 | 50747.3 | 4646532  (98-4646630) | 4335 | 2901 | 72004  (37-72041) |

**Table S6.** Descriptive statistics for socio-demographic factors (**West**)

|  | **Counties**  (N=414) | | | **Tracts**  (N=15195) | | |
| --- | --- | --- | --- | --- | --- | --- |
|  | *Med* | *IQR* | *Range* | *Med* | IQR | *Range* |
| Population density | 4.6 | 18.1 | 7186  (0.08-7186) | 1676.9 | 2659.4 | 50191.2  (0.03-50191.2) |
| Median household income | 53973 | 17080 | 99257  (24798-124055) | 67759 | 42480 | 242540 (7461-250001) |
| Median home value | 205000 | 129525 | 1037900  (59900-1097800) | 355800 | 328400 | 1990002  (9999-2000001) |
| % poverty | 0.13 | 0.06 | 0.31 (0.03-0.35) | 0.1 | 0.11 | 0.8 (0-0.8) |
| Gini index | 0.44 | 0.05 | 0.29 (0.32-0.61) | 0.41 | 0.08 | 0.48 (0.23-0.72) |
| % high school degree | 0.90 | 0.06 | 0.39 (0.6-0.99) | 0.9 | 0.15 | 0.76 (0.24-1) |
| % college degree | 0.23 | 0.13 | 0.6 (0.08-0.67) | 0.28 | 0.29 | 0.96 (0-0.96) |
| % unemployed | 0.41 | 0.11 | 0.49 (0.2-0.69) | 0.36 | 0.11 | 0.92 (0.04-0.96) |
| % employed natural resources | 0.07 | 0.12 | 0.59 (0-0.6) | 0.01 | 0.02 | 0.67 (0-0.67) |
| % NH Black | 0.01 | 0.01 | 0.14 (0-0.14) | 0.02 | 0.05 | 0.85 (0-0.85) |
| % NH Asian | 0.01 | 0.01 | 0.36 (0-0.36) | 0.04 | 0.1 | 0.94 (0-0.94) |
| % Hispanic | 0.12 | 0.21 | 0.84 (0-0.84) | 0.21 | 0.35 | 1 (0-1) |
| % female | 0.5 | 0.02 | 0.22 (0.34-0.56) | 0.5 | 0.04 | 0.63 (0.05-0.67) |
| % 65+ years | 0.19 | 0.09 | 0.32 (0.07-0.39) | 0.14 | 0.09 | 0.91 (0-0.91) |
| Total population | 23648.5 | 82103.75 | 10081129  (441-10081570) | 4566 | 2540 | 31098  (63-31161) |

**
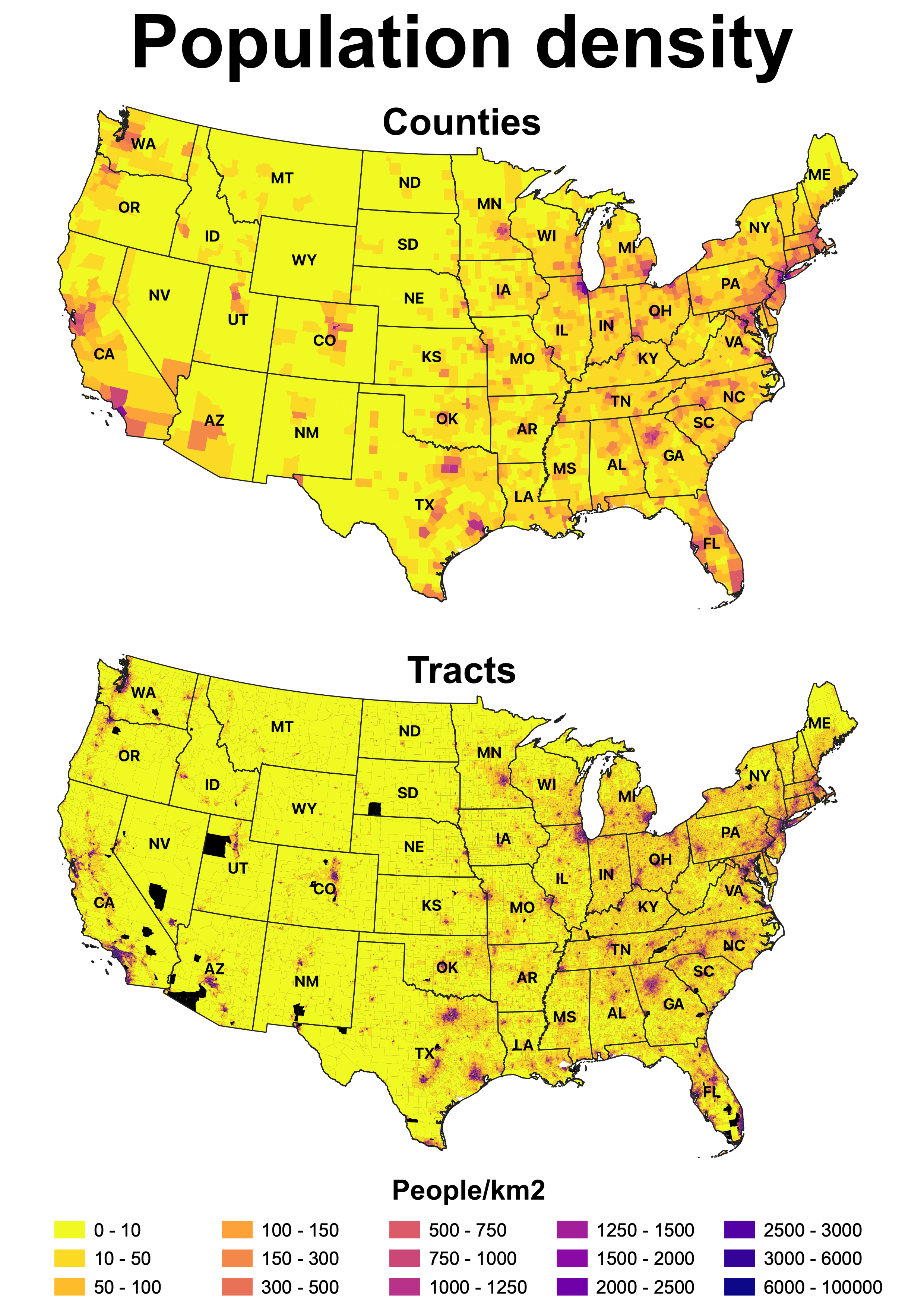
**


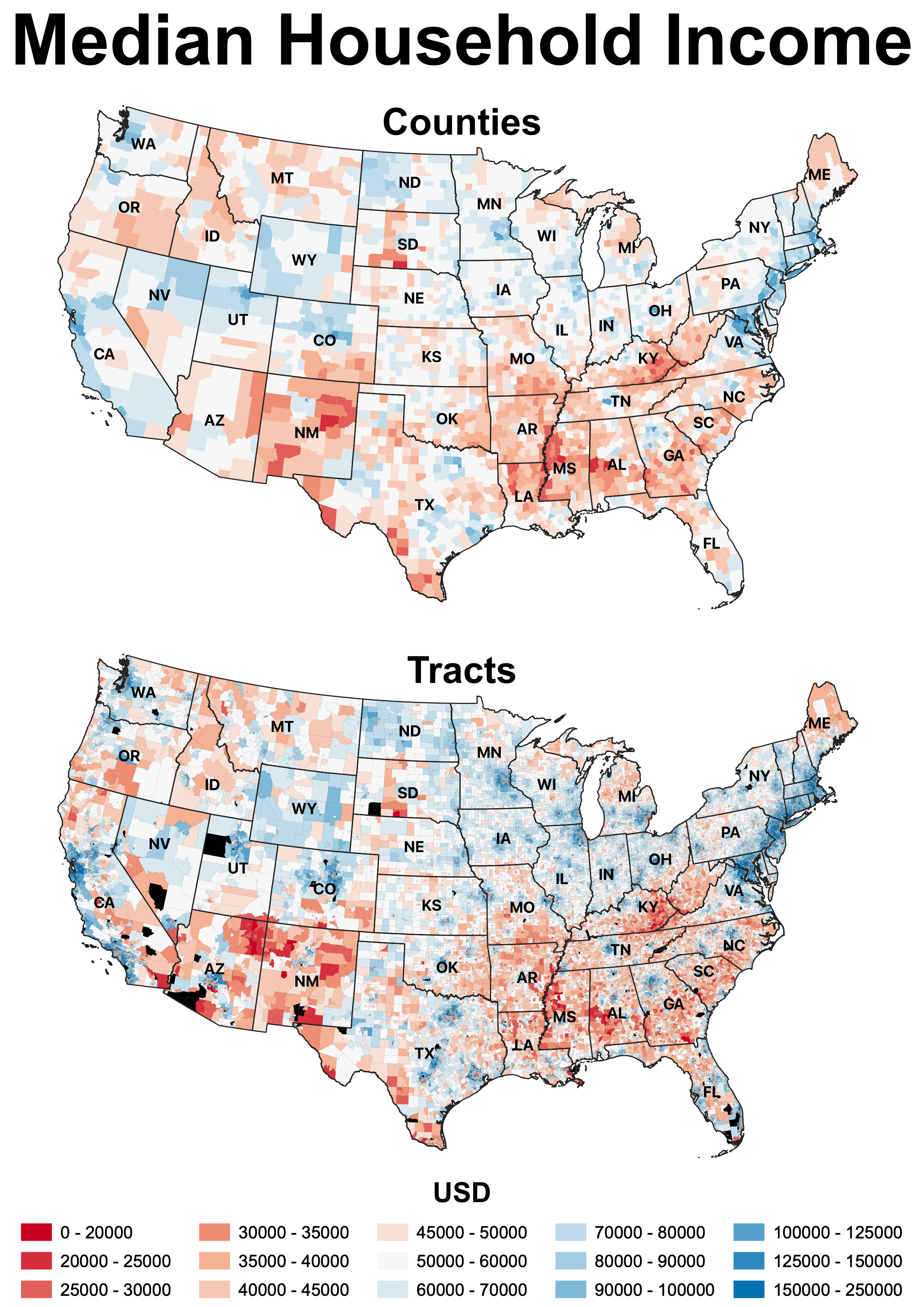


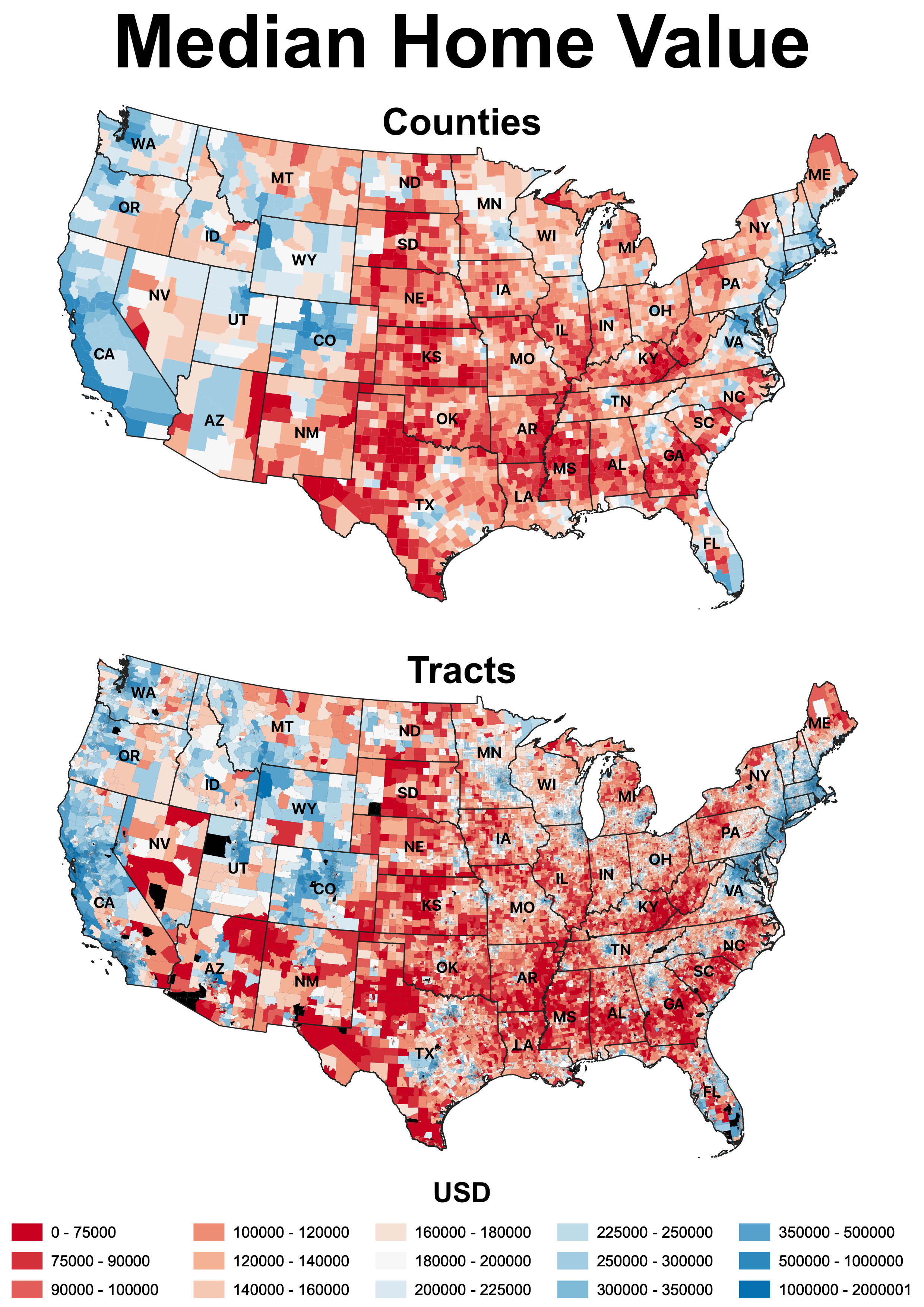


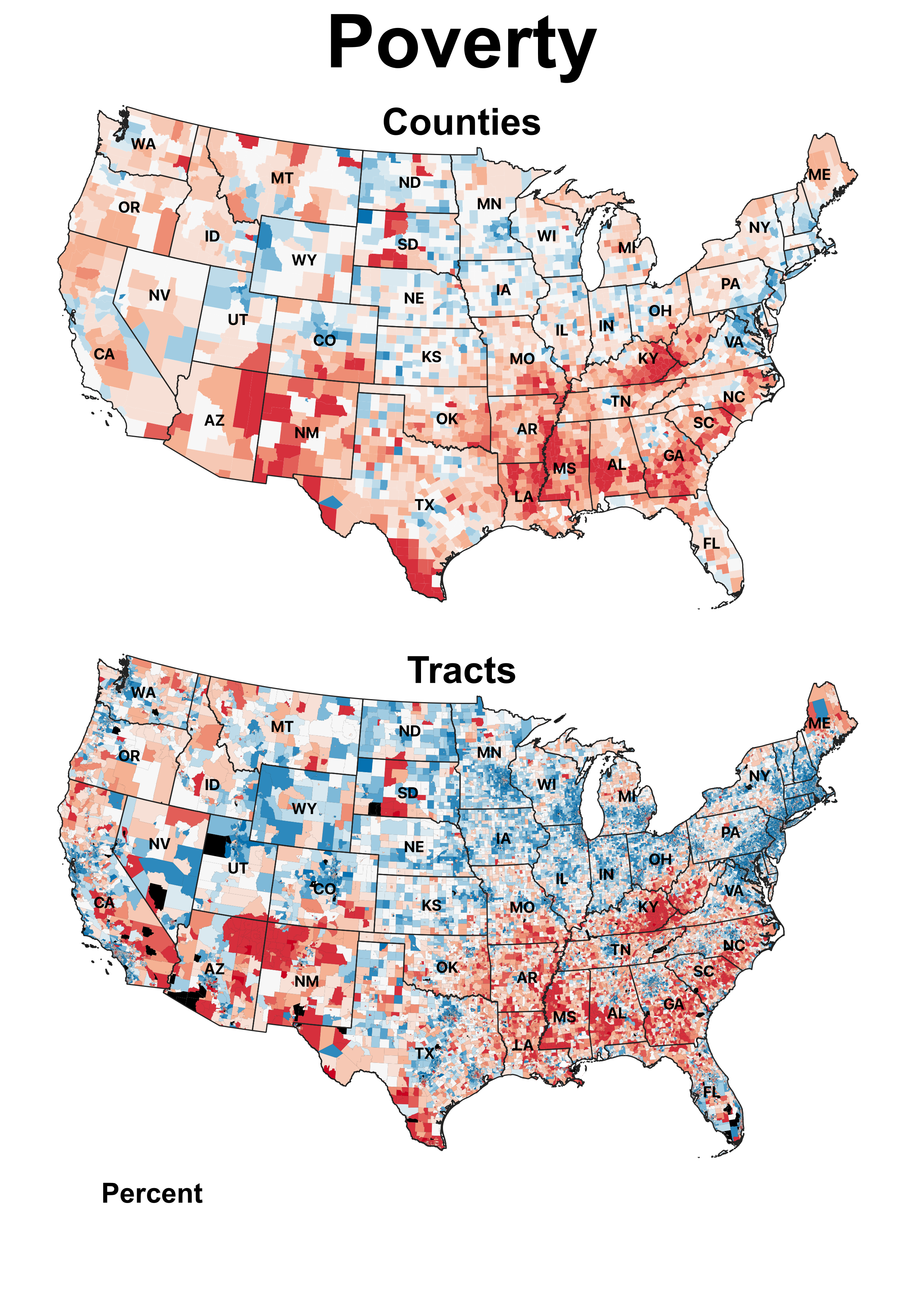


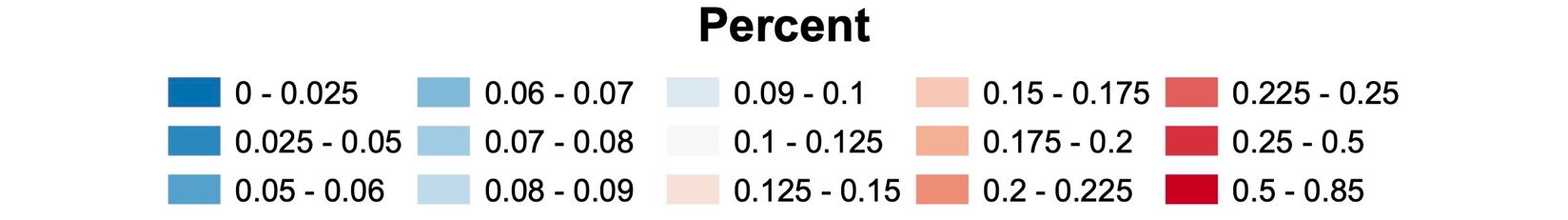


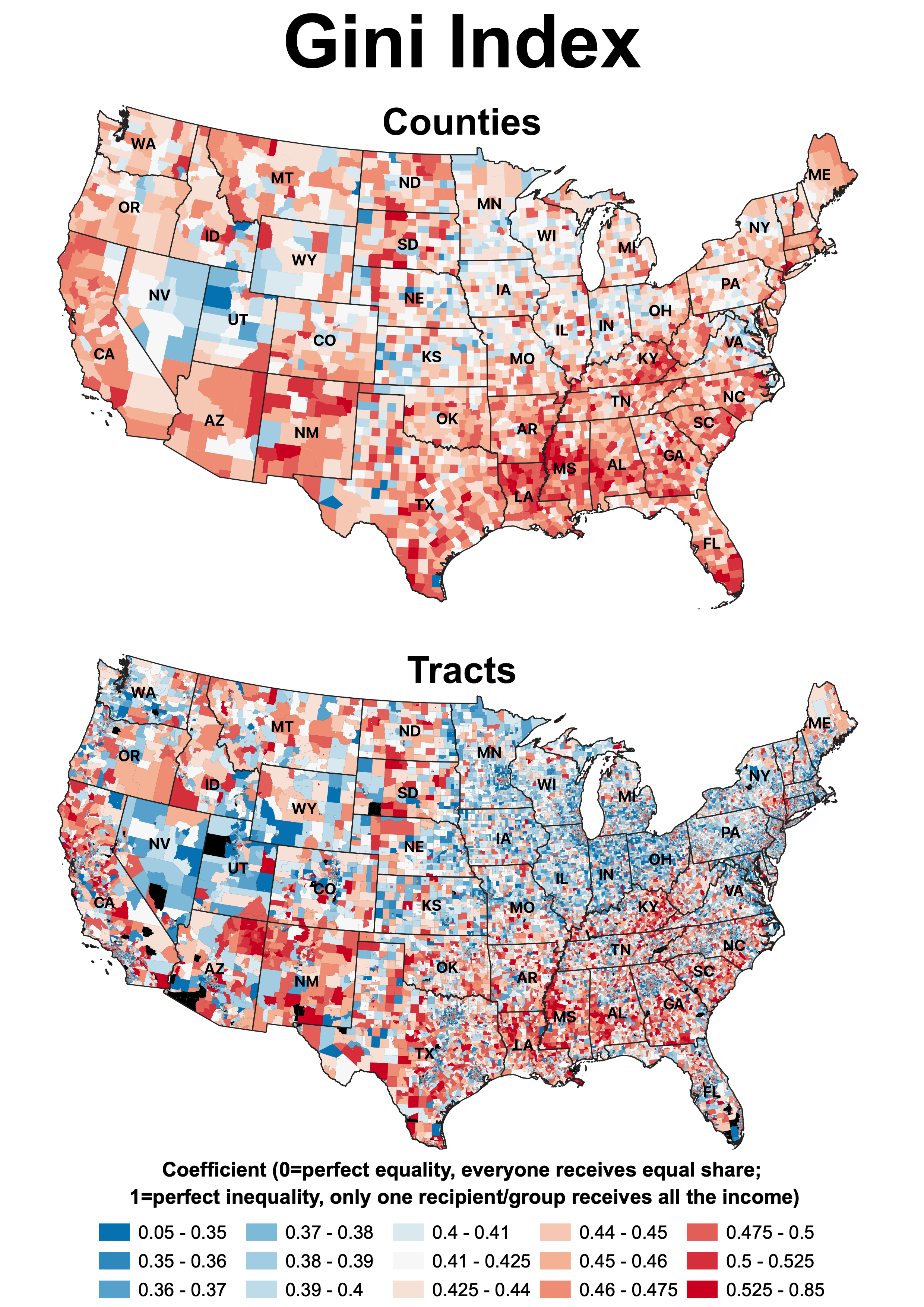


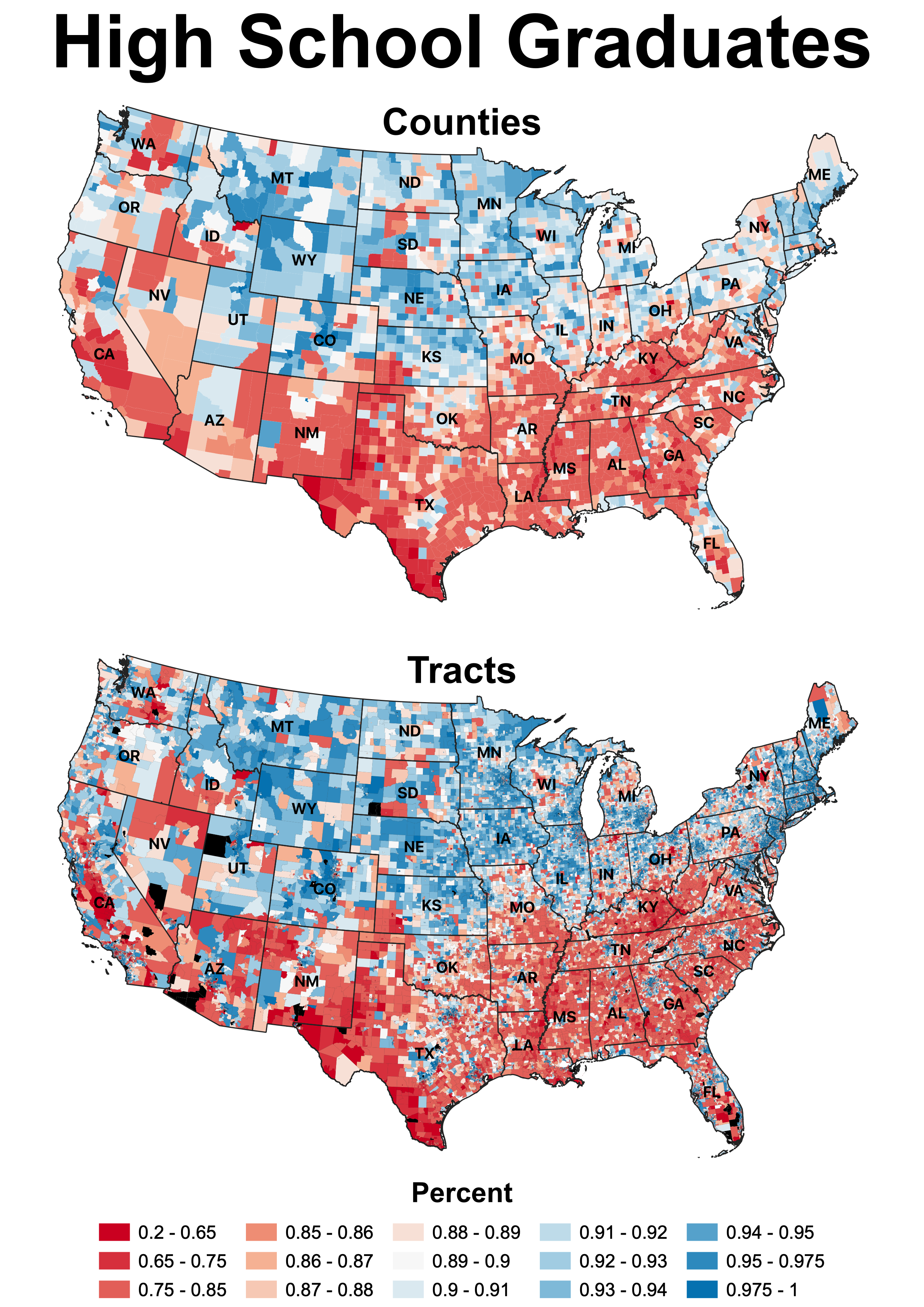


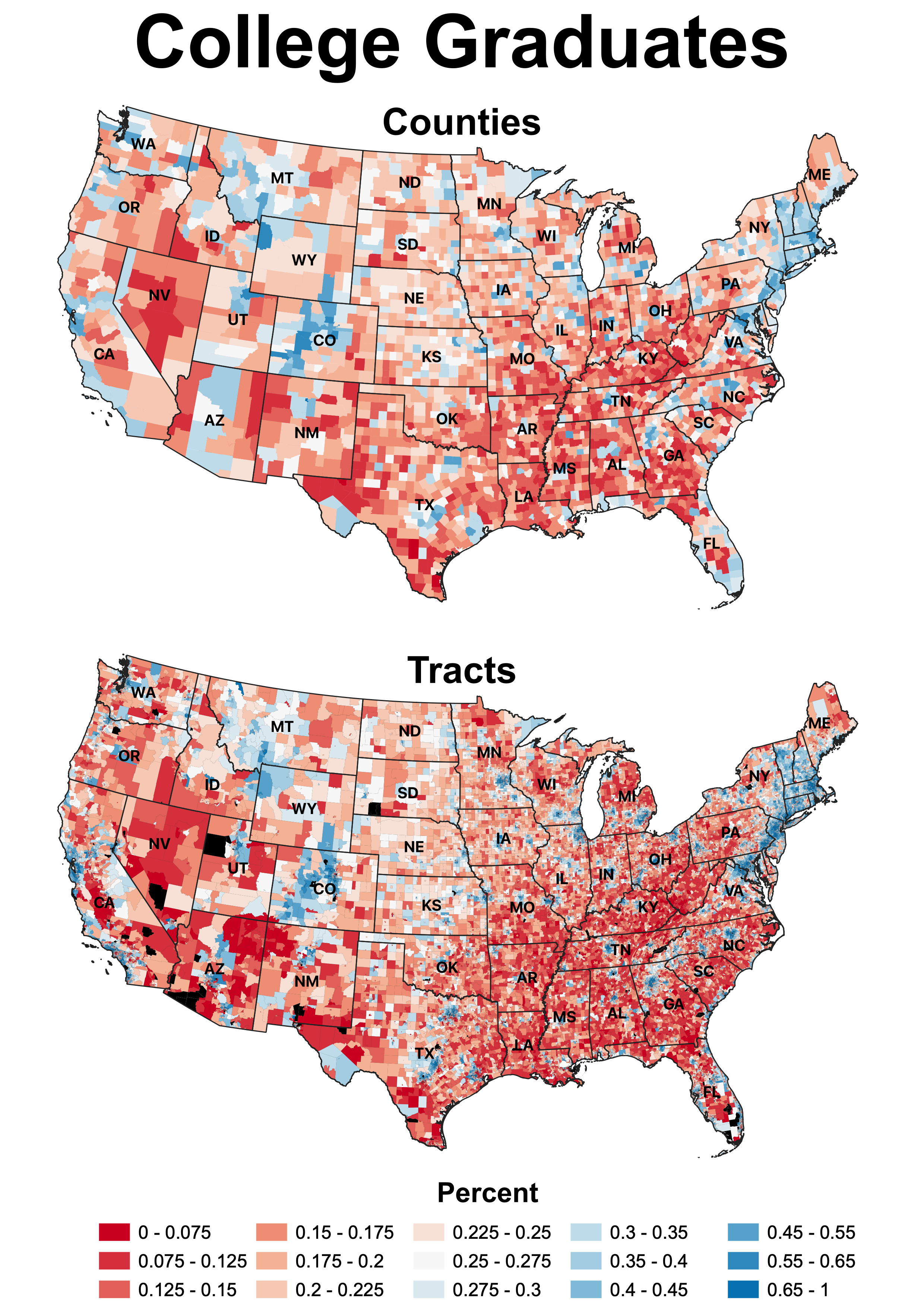

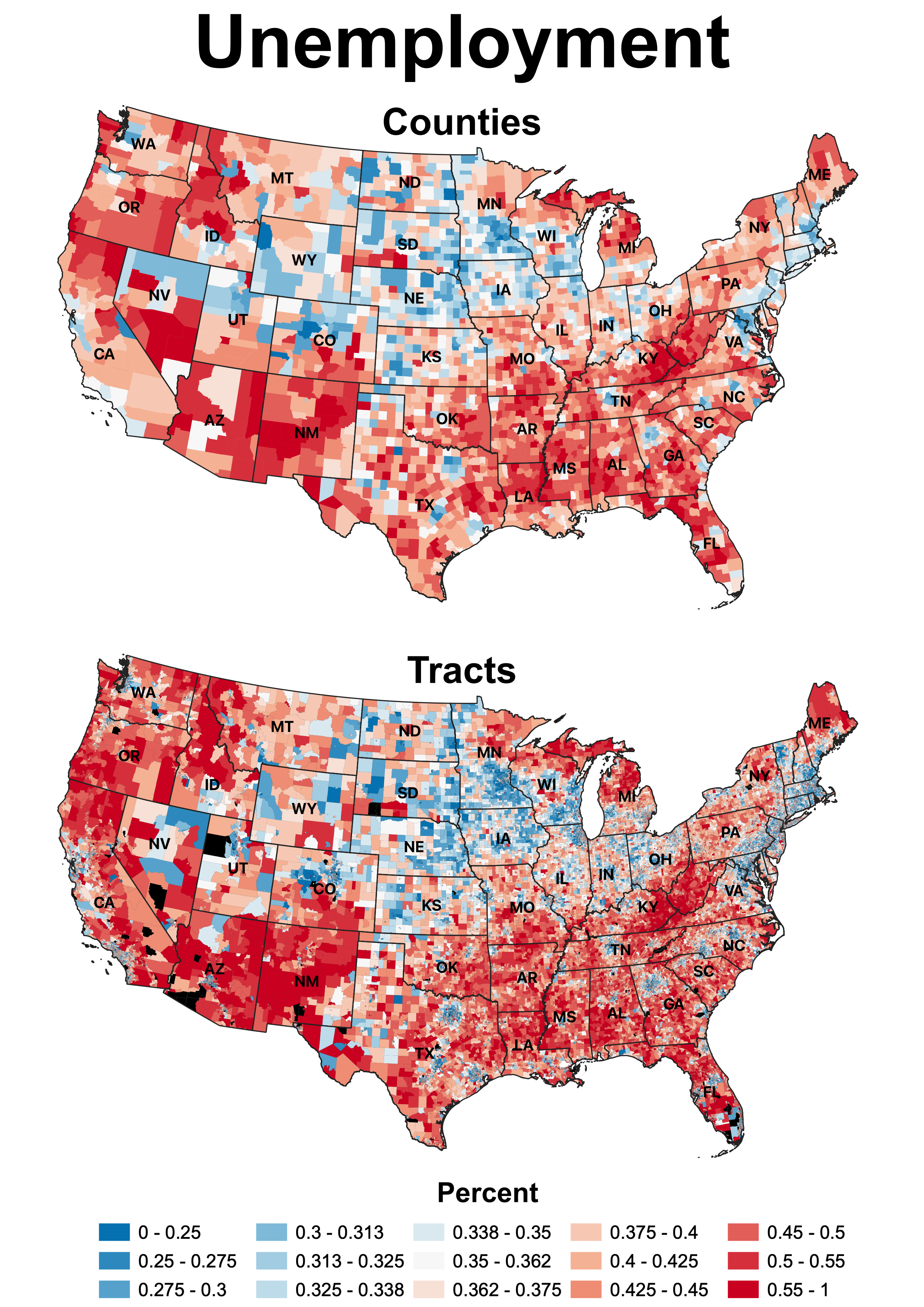

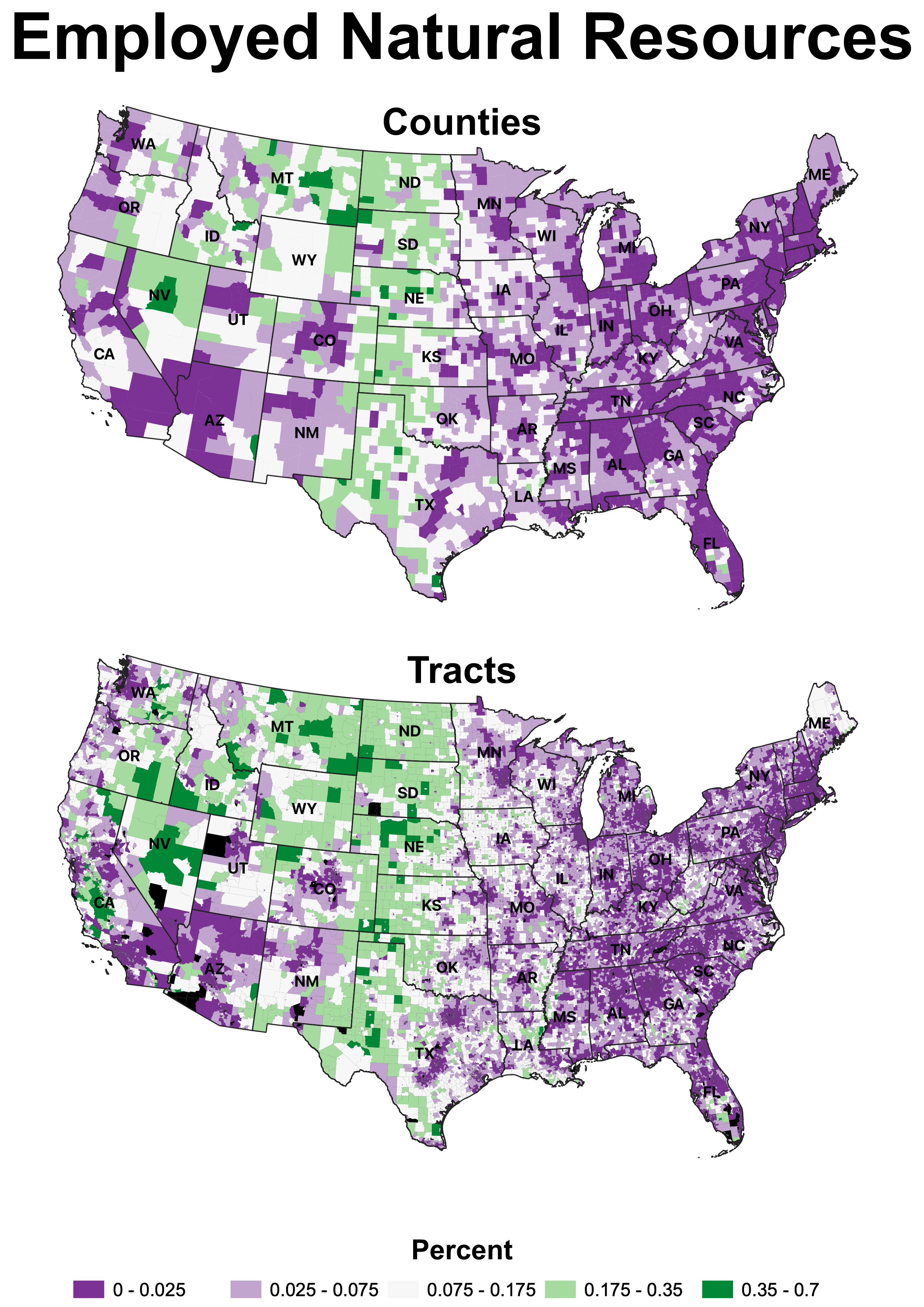

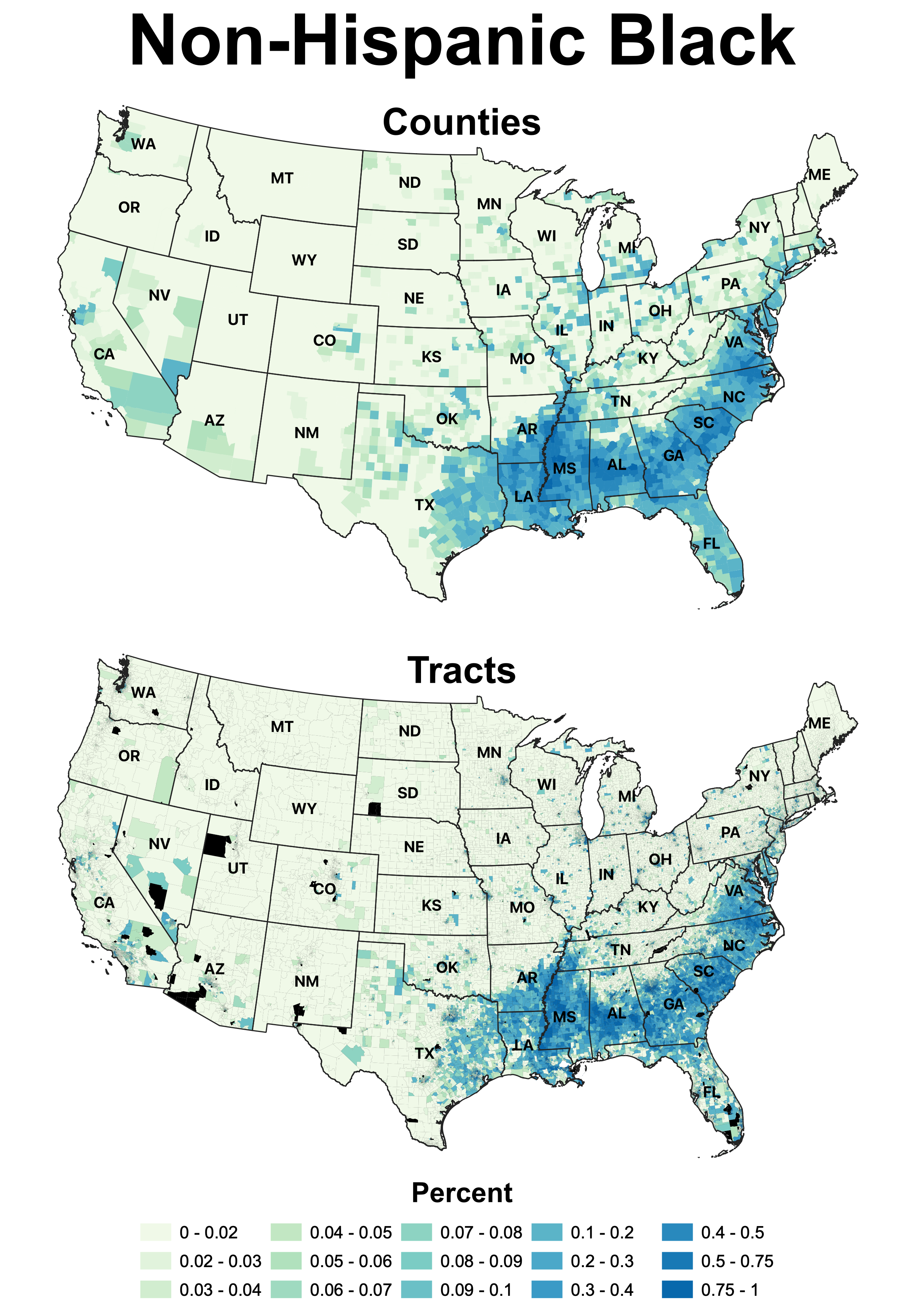

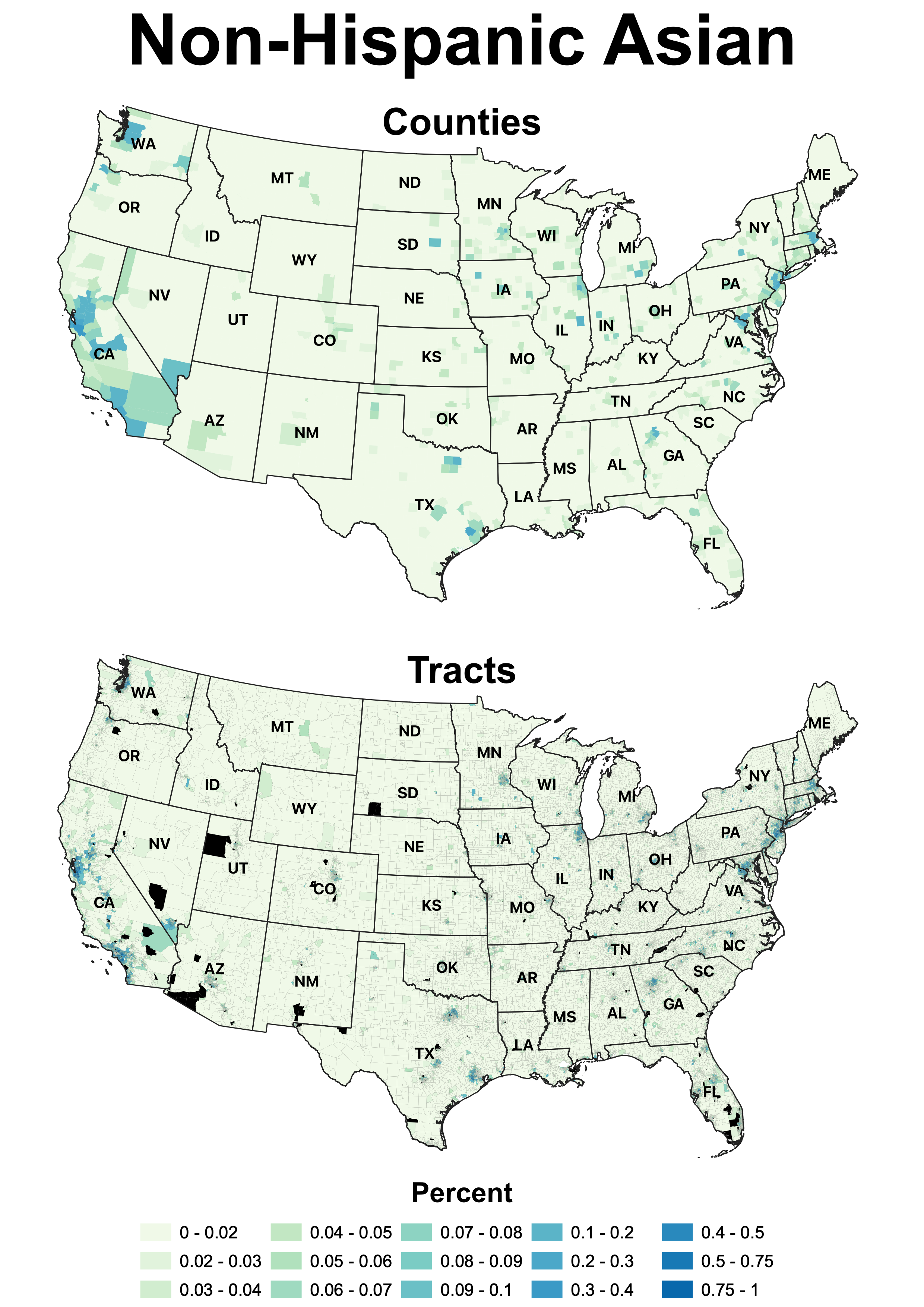

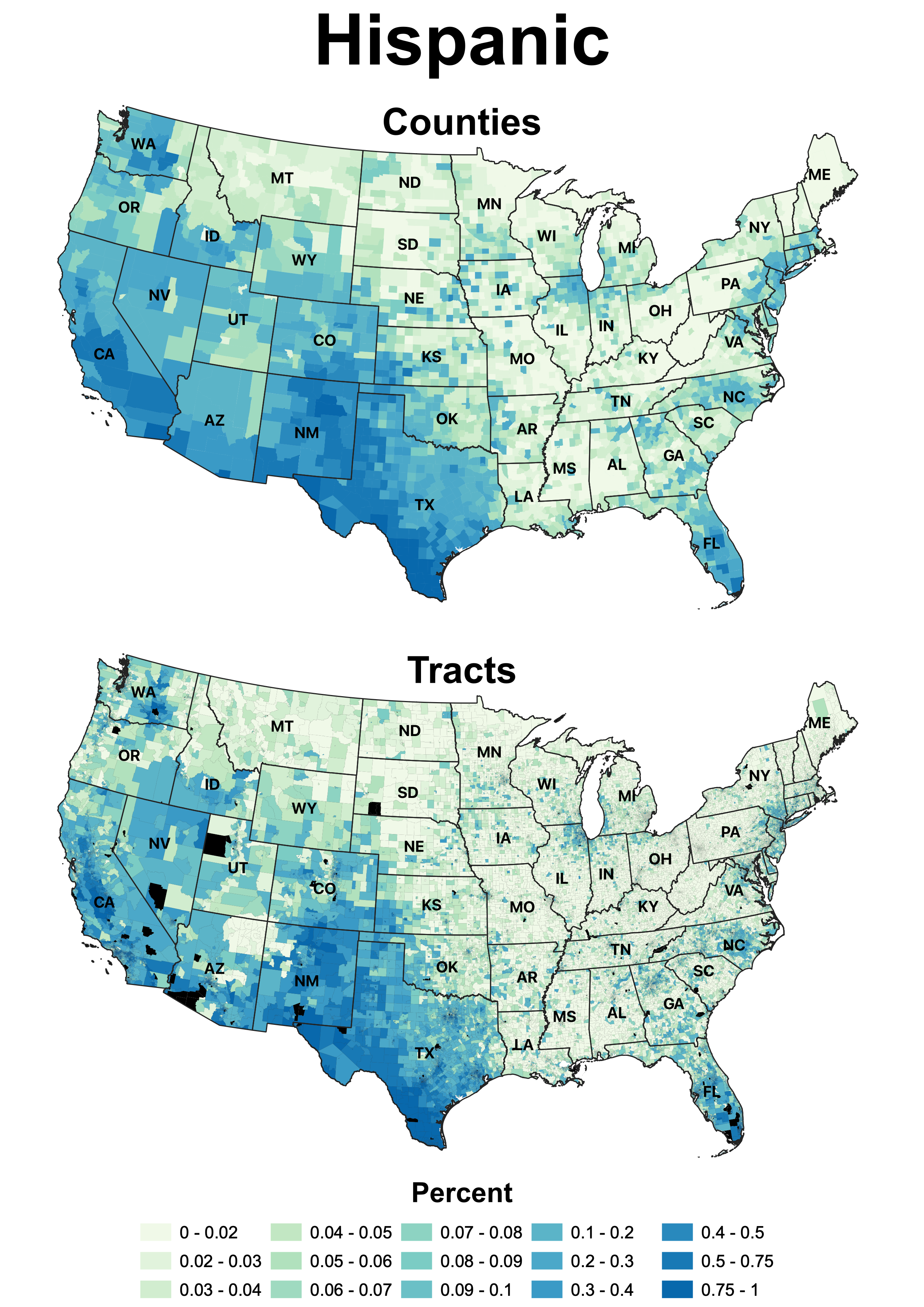


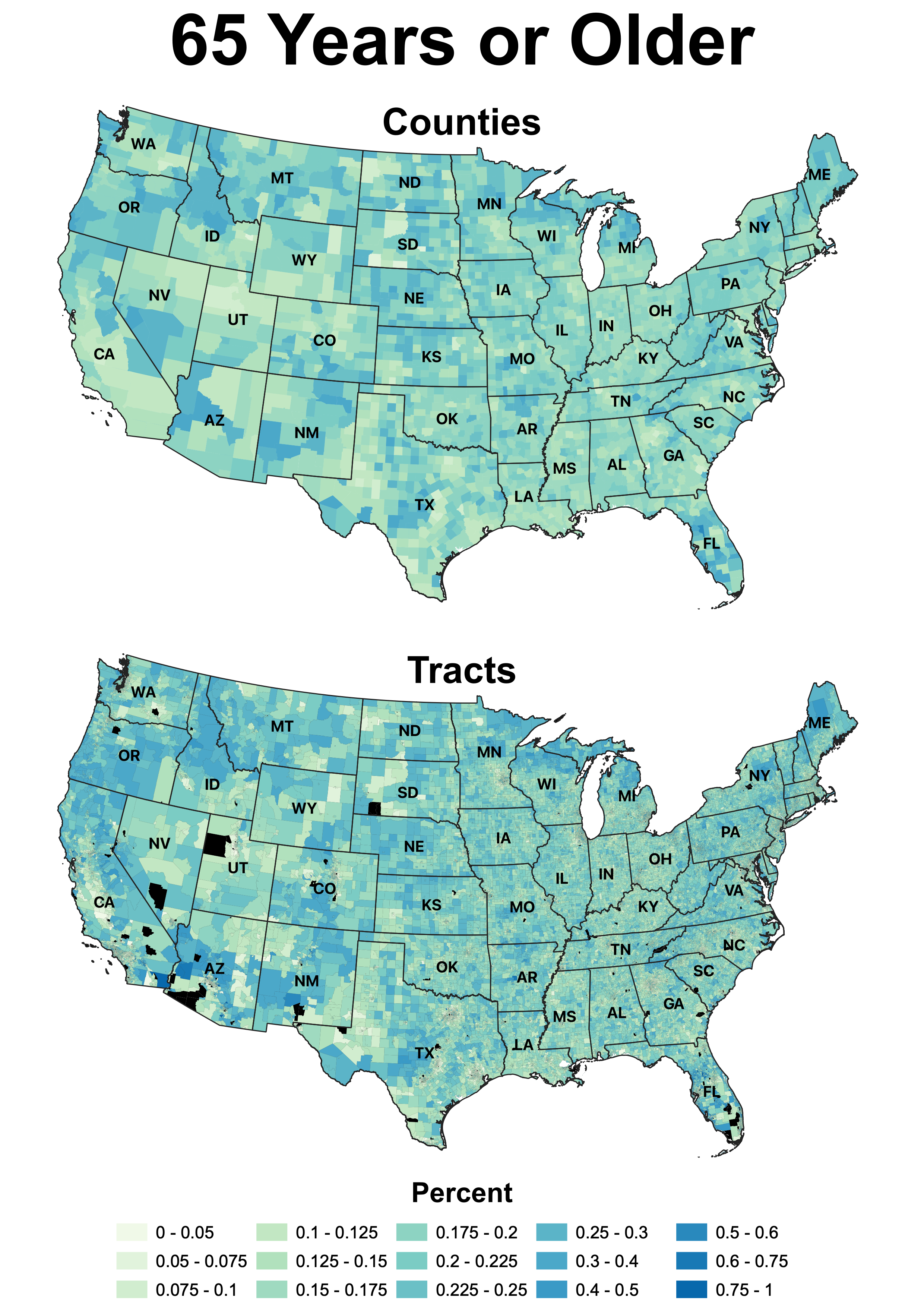

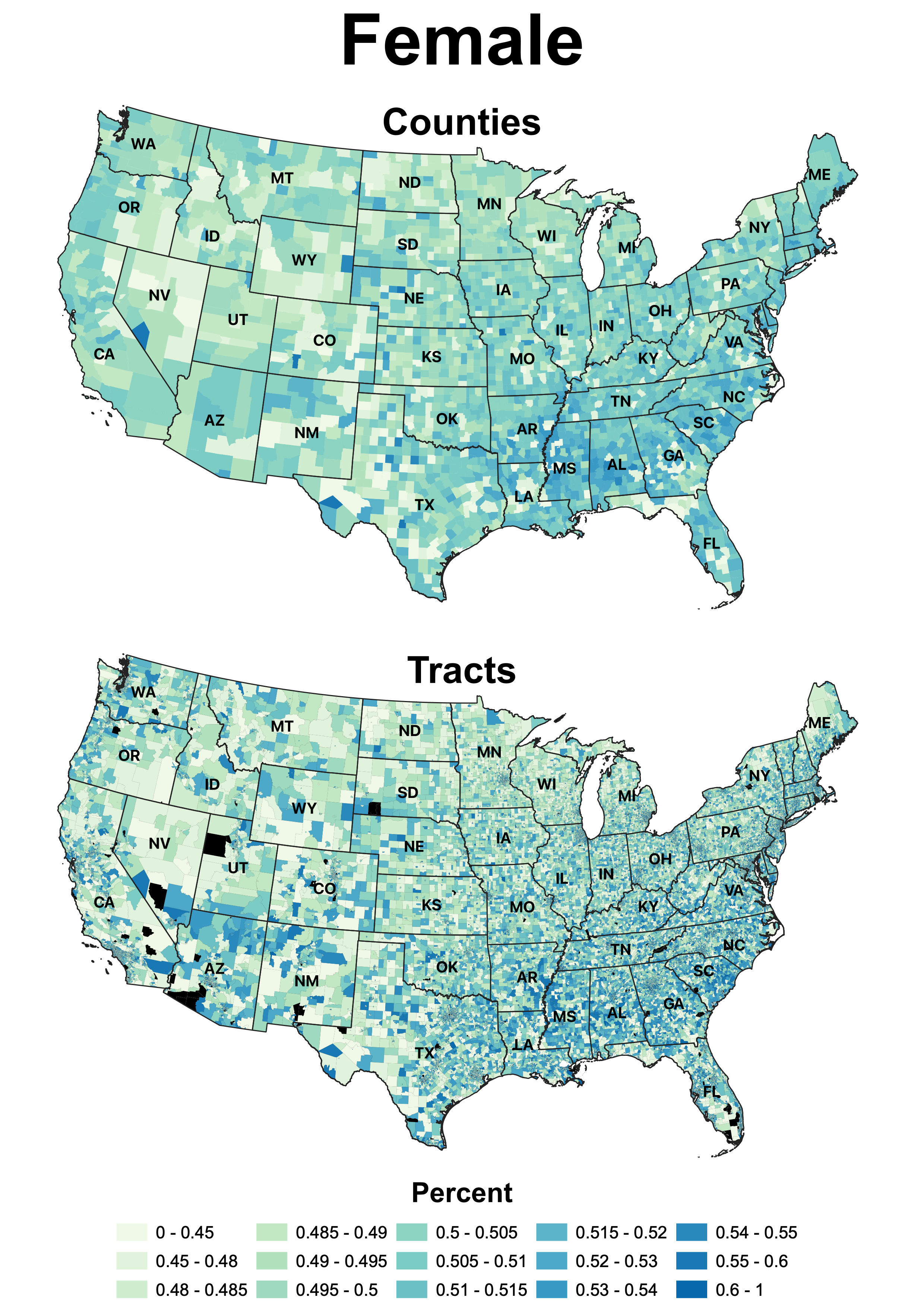

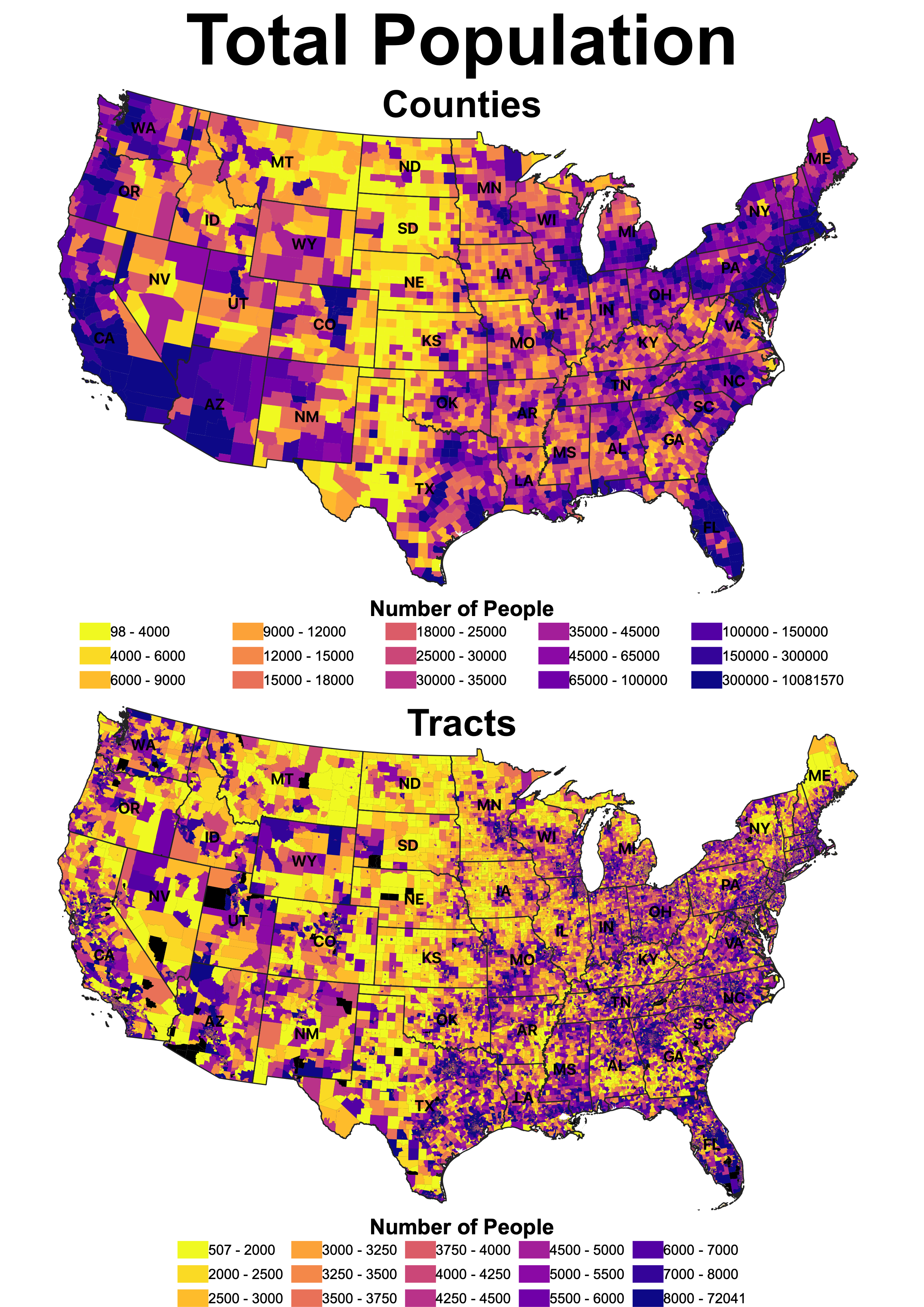


**Figure S8.** Preceding pages include maps of sociodemographic characteristics considered when validating the new public park cover dataset (PAD-US-AR). Note: Tracts with complete data shown (N=70,580). Missing tracts are presented in black.

**Table S7.** Results of generalized linear mixed models (GLMMs) with gamma distributions and U.S. state random effects regressing sociodemographic characteristics on park cover.

|  | **Nationwide** | | | | **Northeast** | | | | **Midwest** | | | | **South** | | | | **West** | | | |
| --- | --- | --- | --- | --- | --- | --- | --- | --- | --- | --- | --- | --- | --- | --- | --- | --- | --- | --- | --- | --- |
|  | **Counties** | | **Tracts** | | **Counties** | | **Tracts** | | **Counties** | | **Tracts** | | **Counties** | | **Tracts** | | **Counties** | | **Tracts** | |
| *Predictors* | *B* | *p* | *B* | *p* | *B* | *p* | *B* | *p* | *B* | *p* | *B* | *p* | *B* | *p* | *B* | *p* | *B* | *p* | *B* | *p* |
| Population density | -0.02 | 0.188 | -0.02 | **<0.001** | 0.08 | 0.402 | 0.08 | **<0.001** | 0.05 | 0.32 | -0.04 | **<0.001** | -0.04 | 0.212 | -0.07 | **<0.001** | -0.08 | 0.119 | -0.11 | **<0.001** |
| Median home value | 0.16 | **<0.001** | 0.05 | **<0.001** | 0.05 | 0.707 | 0.01 | 0.602 | 0.39 | **<0.001** | 0.03 | **0.015** | 0.25 | **<0.001** | 0.06 | **<0.001** | 0.23 | **0.023** | 0.03 | **0.011** |
| % poverty | 0.16 | **<0.001** | 0 | 0.604 | 0.16 | 0.207 | -0.04 | **0.016** | 0.27 | **<0.001** | 0.04 | **0.004** | 0.18 | **0.001** | 0.02 | 0.069 | 0.18 | **0.015** | -0.04 | **0.003** |
| Gini index | -0.09 | **<0.001** | -0.02 | **0.001** | -0.33 | **0.008** | -0.01 | 0.234 | -0.1 | **0.004** | -0.04 | **<0.001** | -0.08 | **0.013** | -0.02 | **0.037** | -0.15 | **0.009** | 0.01 | 0.475 |
| % high school degree | -0.05 | 0.055 | 0.03 | **<0.001** | -0.27 | **0.028** | 0.07 | **<0.001** | -0.03 | 0.537 | 0.03 | **0.04** | -0.04 | 0.43 | -0.03 | **0.004** | -0.12 | 0.165 | -0.04 | 0.051 |
| % college degree | 0.14 | **<0.001** | 0.06 | **<0.001** | 0.32 | 0.078 | 0.04 | **0.006** | 0.05 | 0.35 | 0.11 | **<0.001** | -0.06 | 0.333 | 0.02 | **0.125** | 0.23 | **0.012** | 0.02 | 0.227 |
| % unemployed | 0.04 | **0.165** | 0.09 | **<0.001** | 0.1 | **0.558** | 0.11 | **<0.001** | 0.31 | **<0.001** | 0.08 | **<0.001** | 0.06 | 0.217 | 0.04 | **<0.001** | -0.17 | 0.078 | 0.09 | **<0.001** |
| % employed natural resources | -0.07 | **<0.001** | 0.04 | **<0.001** | -0.23 | **0.006** | 0.04 | **<0.001** | -0.05 | 0.196 | -0.08 | **<0.001** | -0.08 | **0.012** | -0.02 | **0.001** | -0.05 | 0.394 | 0.1 | **<0.001** |
| % NH Black | -0.12 | **<0.001** | -0.01 | **0.004** | 0.05 | 0.634 | -0.02 | 0.144 | -0.02 | 0.571 | 0.02 | 0.117 | -0.27 | **<0.001** | -0.07 | **<0.001** | -0.04 | 0.468 | -0.04 | **<0.001** |
| % NH Asian | -0.14 | **<0.001** | -0.03 | **<0.001** | 0.07 | 0.498 | -0.05 | **<0.001** | -0.03 | 0.426 | 0.04 | **<0.001** | -0.01 | 0.863 | 0.02 | **0.001** | -0.29 | **<0.001** | -0.06 | **<0.001** |
| % Hispanic | -0.01 | 0.637 | -0.06 | **<0.001** | 0 | 0.994 | 0.02 | 0.229 | 0.04 | 0.305 | 0.05 | **<0.001** | -0.04 | 0.418 | -0.06 | **<0.001** | -0.08 | 0.321 | -0.2 | **<0.001** |
| % 65+ years | 0.18 | **<0.001** | 0.06 | **<0.001** | 0.38 | **0.001** | 0.07 | **<0.001** | 0.3 | **<0.001** | 0.09 | **<0.001** | 0.19 | **<0.001** | 0.03 | **0.014** | 0.37 | **<0.001** | 0.06 | **<0.001** |
| % female | -0.07 | **<0.001** | -0.05 | **<0.001** | -0.18 | 0.113 | -0.05 | **<0.001** | -0.08 | **0.007** | -0.04 | **<0.001** | -0.04 | 0.201 | -0.04 | **<0.001** | -0.17 | **0.001** | -0.09 | **<0.001** |
| Total population | 0.06 | **<0.001** | -0.01 | **0.027** | -0.13 | 0.241 | 0.02 | 0.07 | -0.01 | 0.779 | -0.03 | **<0.001** | 0.15 | **<0.001** | -0.01 | 0.059 | 0.09 | 0.066 | -0.03 | **<0.001** |
| **Random Effects** |  |  |  |  |  |  |  |  |  |  |  |  |  |  |  |  |  |  |  |  |
| σ^2^ | 0.01 | | 0.01 | | 0.01 | | 0.01 | | 0.01 | | 0.01 | | 0.01 | | 0.01 | | 0.05 | | 0.02 | |
| τ_00_ | 0.02 _State_ | | 0.00 _State_ | | 0.00 _State_ | | 0.00 _State_ | | 0.00 _State_ | | 0.00 _State_ | | 0.00 _State_ | | 0.00 _State_ | | 0.02 _State_ | | 0.00 _State_ | |
| ICC | 0.6 | | 0.16 | | 0.07 | | 0.06 | | 0.07 | | 0.04 | | 0.09 | | 0.1 | | 0.27 | | 0.03 | |
| N | 49 _State_ | | 49 _State_ | | 9 _State_ | | 9 _State_ | | 12 _State_ | | 12 _State_ | | 17 _State_ | | 17 _State_ | | 11 _State_ | | 11 _State_ | |
| Observations | 3107 | | 70580 | | 217 | | 13022 | | 1055 | | 16762 | | 1421 | | 25601 | | 414 | | 15195 | |
| Marginal R^2^ / Conditional R^2^ | 0.064 / 0.63 | | 0.038 / 0.19 | | 0.24 / 0.29 | | 0.036 / 0.091 | | 0.26 / 0.31 | | 0.038 / 0.078 | | 0.15 / 0.23 | | 0.024 / 0.12 | | 0.16 / 0.38 | | 0.15 / 0.18 | |

Notes: B=standardized betas, σ2 = mean random effect variance, τ_00_ *=* random intercept variance, ICC = intraclass correlation coefficient

**Table S8.** Multicollinearity evaluation of GLMMs with gamma distributions and U.S. state random effects regressing socio-demographics on park cover at the nationwide scale in main model and alternative model with median household income replacing other socioeconomic variables.

|  | **Variance Inflation Factor (VIF) scores** | | | |
| --- | --- | --- | --- | --- |
|  | Main Model | | Alternative Model | |
|  | *Counties* | *Tracts* | *Counties* | *Tracts* |
| Population density | 1.4 | 1.3 | 1.2 | 1.2 |
| Median household income | - | - | 1.5 | 1.4 |
| Median home value | 3.1 | 2.1 | - | - |
| % poverty | 3.4 | 3.4 | - | - |
| Gini index | 1.8 | 1.8 | - | - |
| % high school degree | 3.0 | 4.1 | - | - |
| % college degree | 4.0 | 3.5 | - | - |
| % unemployed | 4.2 | 2.7 | - | - |
| % employed natural resources | 1.3 | 1.2 | - | - |
| % NH Black | 1.2 | 1.5 | 1.2 | 1.3 |
| % NH Asian | 2.0 | 1.3 | 1.7 | 1.2 |
| % Hispanic | 1.8 | 2.4 | 1.2 | 1.4 |
| % 65+ years | 2.6 | 2.7 | 1.5 | 1.3 |
| % female | 1.7 | 1.2 | 1.0 | 1.1 |
| Total population | 1.4 | 1.1 | 1.4 | 1.1 |

**Table S9.** Results of generalized linear mixed models (GLMMs) with gamma distributions and U.S. state random effects regressing sociodemographic characteristics on park cover substituting median household income for other socio-economic indicators.

|  | **Nationwide** | | | |  | **Northeast** | | | |  | **Midwest** | | | |  | **South** | | | |  | **West** | | | |
| --- | --- | --- | --- | --- | --- | --- | --- | --- | --- | --- | --- | --- | --- | --- | --- | --- | --- | --- | --- | --- | --- | --- | --- | --- |
|  | **Counties** | | **Tracts** | |  | **Counties** | | **Tracts** | |  | **Counties** | | **Tracts** | |  | **Counties** | | **Tracts** | |  | **Counties** | | **Tracts** | |
| *Predictors* | *B* | *p* | *B* | *p* |  | *B* | *p* | *B* | *p* |  | *B* | *p* | *B* | *p* |  | *B* | *p* | *B* | *p* |  | *B* | *p* | *B* | *p* |
| Population density | 0.01 | 0.423 | -0.01 | **0.028** |  | 0.08 | 0.337 | 0.07 | **<0.001** | | 0.04 | 0.454 | -0.01 | 0.453 |  | 0 | 0.89 | -0.07 | **<0.001** | | -0.05 | 0.333 | -0.15 | **<0.001** |
| Median household income | -0.01 | 0.564 | 0.04 | **<0.001** | | -0.01 | 0.951 | 0.06 | **<0.001** | | -0.13 | **<0.001** | 0.07 | **<0.001** | | -0.1 | **0.004** | 0.02 | **0.018** |  | 0.16 | **0.01** | -0.01 | 0.239 |
| % NH Black | -0.12 | **<0.001** | -0.01 | **0.001** |  | 0.05 | 0.578 | -0.02 | **0.041** |  | -0.03 | 0.516 | 0.04 | **<0.001** | | -0.28 | **<0.001** | -0.06 | **<0.001** | | -0.09 | 0.116 | -0.06 | **<0.001** |
| % NH Asian | -0.04 | **0.039** | -0.03 | **<0.001** | | 0.13 | 0.196 | -0.05 | **<0.001** | | 0.07 | **0.042** | 0.07 | **<0.001** | | 0.06 | 0.067 | 0.03 | **<0.001** | | -0.18 | **0.021** | -0.06 | **<0.001** |
| % Hispanic | -0.03 | 0.197 | -0.08 | **<0.001** | | 0.13 | 0.149 | -0.02 | **0.05** |  | -0.03 | 0.424 | 0.02 | **0.011** |  | -0.04 | 0.244 | -0.04 | **<0.001** | | -0.07 | 0.302 | -0.16 | **<0.001** |
| % 65+ years | 0.15 | **<0.001** | 0.12 | **<0.001** | | 0.35 | **<0.001** | 0.14 | **<0.001** | | 0.21 | **<0.001** | 0.12 | **<0.001** | | 0.19 | **<0.001** | 0.05 | **<0.001** | | 0.21 | **<0.001** | 0.14 | **<0.001** |
| % female | -0.06 | **<0.001** | -0.07 | **<0.001** | | -0.28 | **<0.001** | -0.06 | **<0.001** | | -0.12 | **<0.001** | -0.04 | **<0.001** | | -0.06 | **0.016** | -0.04 | **<0.001** | | -0.1 | **0.037** | -0.12 | **<0.001** |
| Total population | 0.06 | **<0.001** | -0.01 | **0.001** |  | -0.11 | 0.349 | 0.02 | 0.061 |  | 0.02 | 0.631 | -0.02 | **0.016** |  | 0.13 | **<0.001** | -0.02 | **0.006** |  | 0.11 | **0.04** | -0.03 | **<0.001** |
| **Random Effects** |  |  |  |  |  |  |  |  |  |  |  |  |  |  |  |  |  |  |  |  |  |  |  |  |
| σ^2^ | 0.02 | | 0.01 | |  | 0.01 | | 0.01 | |  | 0.01 | | 0.01 | |  | 0.01 | | 0.01 | |  | 0.06 | | 0.02 | |
| τ_00_ | 0.02 _State_ | | 0.00 _State_ | |  | 0.00 _State_ | | 0.00 _State_ | |  | 0.00 _State_ | | 0.00 _State_ | |  | 0.00 _State_ | | 0.00 _State_ | |  | 0.01 _State_ | | 0.00 _State_ | |
| ICC | 0.61 | | 0.17 | |  | 0.05 | | 0.05 | |  | 0.16 | | 0.04 | |  | 0.13 | | 0.11 | |  | 0.21 | | 0.03 | |
| N | 49 _State_ | | 49 _State_ | |  | 9 _State_ | | 9 _State_ | |  | 12 _State_ | | 12 _State_ | |  | 17 _State_ | | 17 _State_ | |  | 11 _State_ | | 11 _State_ | |
| Observations | 3108 | | 70580 | |  | 217 | | 13022 | |  | 1055 | | 16762 | |  | 1422 | | 25601 | |  | 414 | | 15195 | |
| Marginal R^2^ / Conditional R^2^ | 0.038 / 0.63 | | 0.033 / 0.19 | |  | 0.17 / 0.21 | | 0.029 / 0.075 | |  | 0.077 / 0.23 | | 0.020 / 0.058 | |  | 0.12 / 0.24 | | 0.020 / 0.13 | |  | 0.10 / 0.29 | | 0.14 / 0.17 | |

Notes: B=standardized betas, σ2 = mean random effect variance, τ_00_ *=* random intercept variance, ICC = intraclass correlation coefficient

**Table S10.** Results of GLMMs with gamma distributions and U.S. state random effects or LM^a^ regressing sociodemographic characteristics on park cover in more urban areas.

|  | **Nationwide** | | | | **Northeast** | | | | **Midwest** | | | | **South** | | | | **West** | | | |
| --- | --- | --- | --- | --- | --- | --- | --- | --- | --- | --- | --- | --- | --- | --- | --- | --- | --- | --- | --- | --- |
|  | **Counties** | | **Tracts** | | **Counties** | | **Tracts** | | **Counties** | | **Tracts** | | **Counties** | | **Tracts** | | **Counties** | | **Tracts** | |
| *Predictors* | *B* | *p* | *B* | *p* | *B* | *p* | *B* | *p* | *B* | *p* | *B* | *p* | *B* | *p* | *B* | *p* | *B* | *p* | *B* | *p* |
| Population density | -0.05 | 0.153 | 0.02 | **0.006** | 0.29 | **0.024** | 0.08 | **<0.001** | 0.29 | 0.094 | -0.05 | **<0.001** | -0.14 | **0.024** | -0.05 | **<0.001** | -0.26 | 0.106 | -0.08 | **<0.001** |
| Median home value | 0.15 | **0.028** | 0.05 | **<0.001** | -0.02 | 0.891 | -0.01 | 0.78 | 0.55 | **0.008** | -0.08 | **<0.001** | 0.42 | **<0.001** | 0.05 | **0.003** | -0.14 | 0.685 | 0.02 | 0.253 |
| % poverty | 0.13 | 0.057 | 0.01 | 0.178 | -0.08 | 0.675 | 0.01 | 0.526 | 0.23 | 0.276 | -0.02 | 0.445 | 0.21 | 0.057 | 0.01 | 0.559 | -0.25 | 0.461 | 0 | 0.871 |
| Gini index | -0.09 | 0.096 | -0.01 | 0.087 | -0.5 | **0.002** | 0.02 | 0.241 | 0.05 | 0.794 | 0.01 | 0.426 | 0.03 | 0.723 | -0.01 | 0.663 | 0.31 | 0.269 | -0.03 | **0.011** |
| % high school degree | -0.01 | 0.841 | 0.04 | **<0.001** | -0.23 | 0.31 | 0.11 | **<0.001** | 0.2 | 0.159 | 0.02 | 0.439 | 0.04 | 0.627 | -0.04 | **0.042** | 0.38 | 0.531 | -0.08 | **0.002** |
| % college degree | 0.16 | **0.033** | 0.15 | **<0.001** | 0.52 | **0.018** | 0.19 | **<0.001** | -0.27 | 0.301 | 0.18 | **<0.001** | -0.22 | 0.077 | 0.17 | **<0.001** | 0.08 | 0.819 | 0.18 | **<0.001** |
| % unemployed | 0.08 | 0.249 | 0 | 0.91 | 0.49 | **0.003** | 0.07 | **<0.001** | 0.31 | 0.103 | -0.02 | 0.222 | -0.04 | 0.765 | -0.05 | **0.001** | 0.03 | 0.92 | -0.04 | **0.019** |
| % employed natural resources | -0.07 | **0.028** | -0.02 | **0.004** | -0.33 | **0.002** | -0.02 | 0.068 | -0.09 | 0.346 | 0 | 0.943 | -0.1 | **0.029** | -0.04 | **<0.001** | -0.06 | 0.832 | -0.02 | 0.076 |
| % NH Black | -0.06 | 0.145 | 0.05 | **<0.001** | 0.2 | 0.11 | 0.06 | **<0.001** | -0.17 | 0.227 | 0.05 | **0.005** | -0.07 | 0.207 | 0.11 | **<0.001** | -0.06 | 0.696 | -0.04 | **<0.001** |
| % NH Asian | -0.11 | **0.012** | 0.02 | **0.002** | -0.18 | 0.144 | -0.01 | 0.689 | 0.06 | 0.639 | 0.07 | **<0.001** | 0.03 | 0.598 | 0.04 | **<0.001** | -0.17 | 0.469 | 0 | 0.926 |
| % Hispanic | 0.08 | 0.161 | 0.04 | **<0.001** | 0.15 | 0.37 | 0.17 | **<0.001** | -0.07 | 0.507 | -0.01 | 0.718 | 0.09 | 0.218 | 0.08 | **<0.001** | 0.14 | 0.683 | -0.12 | **<0.001** |
| % 65+ years | 0.07 | 0.283 | 0.03 | **<0.001** | 0 | 0.976 | 0.07 | **<0.001** | -0.04 | 0.772 | 0 | 0.799 | 0.24 | **0.032** | 0.02 | 0.174 | -0.17 | 0.48 | 0.03 | 0.111 |
| % female | -0.05 | 0.215 | 0 | 0.854 | -0.27 | **0.014** | 0.02 | 0.123 | 0.05 | 0.663 | 0.02 | 0.154 | -0.02 | 0.732 | -0.02 | **0.04** | -0.31 | **0.017** | -0.01 | 0.416 |
| Total population | 0.19 | **<0.001** | 0.04 | **<0.001** | -0.09 | 0.486 | 0.07 | **<0.001** | -0.02 | 0.882 | 0.04 | **0.005** | 0.22 | **<0.001** | 0 | 0.873 | 0.26 | 0.089 | 0.02 | **0.018** |
| **Random Effects** |  |  |  |  |  |  |  |  |  |  |  |  |  |  |  |  |  |  |  |  |
| σ^2^ | 0.01 | | 0 | | 0 | | 0 | | NA | | 0 | | 0 | | 0 | | 0.03 | | 0 | |
| τ_00_ | 0.01 _State_ | | 0.00 _State_ | | 0.00 _State_ | | 0.00 _State_ | | NA | | 0.00 _State_ | | 0.00 _State_ | | 0.00 _State_ | | 0.01 _State_ | | 0.00 _State_ | |
| ICC | 0.64 | | 0.11 | | 0.16 | | 0.01 | | NA | | 0.06 | | 0.15 | | 0.2 | | 0.24 | | 0.11 | |
| N | 46 _State_ | | 49 _State_ | | 9 _State_ | | 9 _State_ | | NA | | 12 _State_ | | 17 _State_ | | 17 _State_ | | 9 _State_ | | 11 _State_ | |
| Observations | 743 | | 32929 | | 121 | | 7242 | | 160 | | 6657 | | 386 | | 9314 | | 58 | | 9716 | |
| Marginal R^2^ / Conditional R^2^ | 0.053 / 0.657 | | 0.038 / 0.142 | | 0.302 / 0.413 | | 0.072 / 0.082 | | 0.186 / 0.108 | | 0.037 / 0.093 | | 0.173 / 0.297 | | 0.033 / 0.225 | | 0.328 / 0.491 | | 0.072 / 0.178 | |

Notes: ^a^ the small number of urban counties in several midwestern states required standard linear regression rather than mixed-effect models; urban areas were identified as counties with ≥50 people/km^2^ and as tracts with ≥1,000 people/km^2^; GLMMs=generalized linear mixed models, LM=standard linear model; B=standardized betas, σ2 = mean random effect variance, τ_00_ *=* random intercept variance, ICC = intraclass correlation coefficient

**
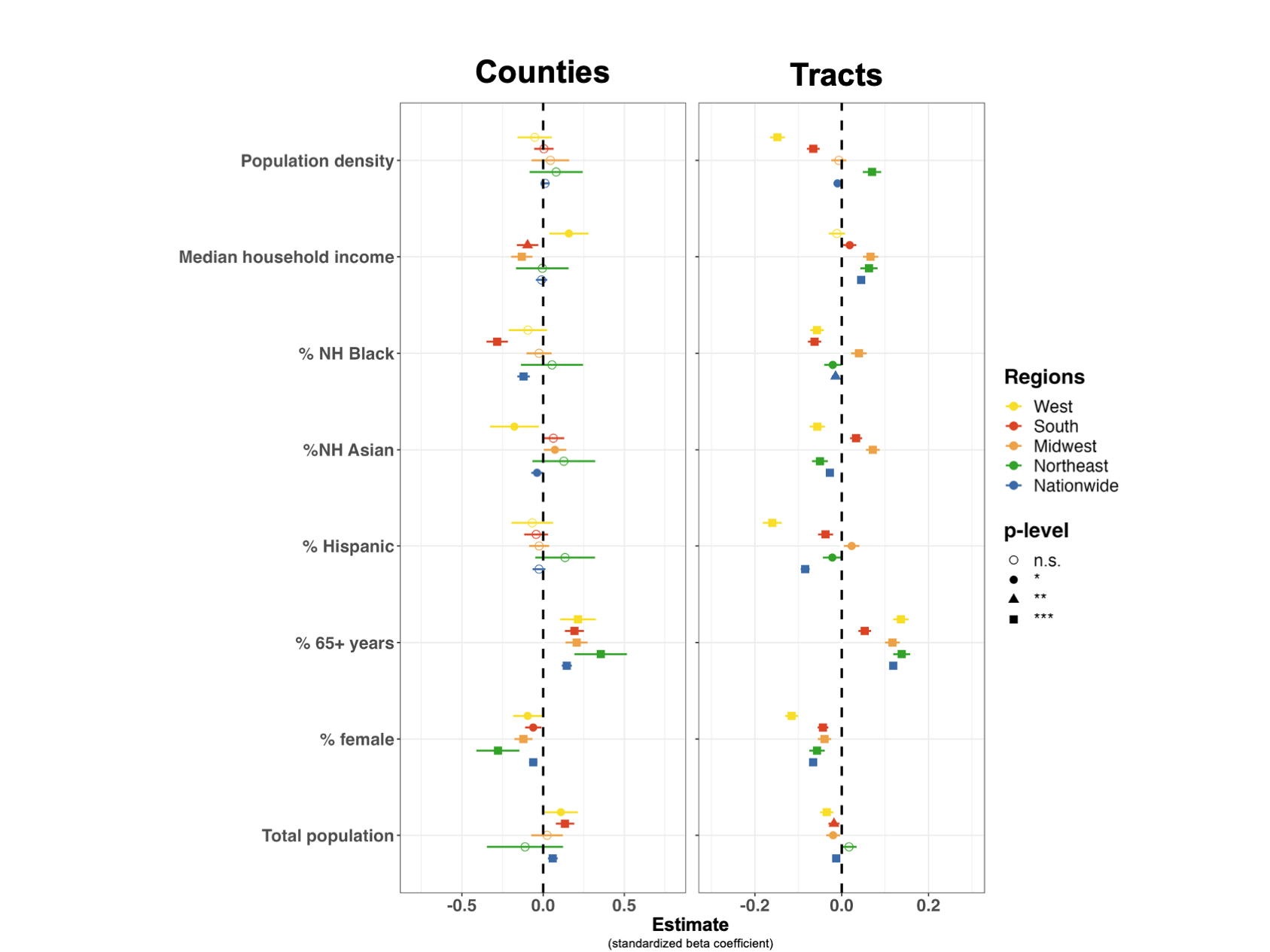
Figure S9.** Regressing sociodemographic characteristics on the PAD-US-AR park cover dataset within counties and tracts. Notes: GLMMs with gamma distributions and U.S. state random effects, except for Midwestern counties which report standard linear regression results. Standardized betas and 95% confidence intervals are shown. Differing symbols represent statistical significance (p-value): empty circle is shown for *p* >.05, filled-in circle for *p* < .05; triangle for *p* < .01; square for *p* < .001. X-axes are on different scales.
